# Population Mobility Trends, Deprivation Index and the Spatio-Temporal Spread of Coronavirus Disease 2019 in Ireland

**DOI:** 10.3390/ijerph18126285

**Published:** 2021-06-10

**Authors:** Jamie M. Madden, Simon More, Conor Teljeur, Justin Gleeson, Cathal Walsh, Guy McGrath

**Affiliations:** 1 Centre for Veterinary Epidemiology and Risk Analysis (CVERA), School of Veterinary Medicine, University College Dublin, D04 W6F6 Dublin, Ireland; simon.more@ucd.ie (S.M.); guy.mcgrath@ucd.ie (G.M.); 2 Health Technology Assessment Directorate, Health Information and Quality Authority, D07 E98Y Dublin, Ireland; cteljeur@hiqa.ie; 3 National Institute for Regional and Spatial Analysis, National University of Ireland Maynooth, W23 F2H6 Kildare, Ireland; justin.gleeson@mu.ie; 4 Health Research Institute and MACSI, University of Limerick, V94 T9PX Limerick, Ireland; Cathal.Walsh@ul.ie

**Keywords:** COVID-19, spatio-temporal, spatial statistics, standardised incidence ratio, disease mapping

## Abstract

Like most countries worldwide, the coronavirus disease (COVID-19) has adversely affected Ireland. The aim of this study was to (i) investigate the spatio-temporal trend of COVID-19 incidence; (ii) describe mobility trends as measured by aggregated mobile phone records; and (iii) investigate the association between deprivation index, population density and COVID-19 cases while accounting for spatial and temporal correlation. Standardised incidence ratios of cases were calculated and mapped at a high spatial resolution (electoral division level) over time. Trends in the percentage change in mobility compared to a pre-COVID-19 period were plotted to investigate the impact of lockdown restrictions. We implemented a hierarchical Bayesian spatio-temporal model (Besag, York and Mollié (BYM)), commonly used for disease mapping, to investigate the association between covariates and the number of cases. There have been three distinct “waves” of COVID-19 cases in Ireland to date. Lockdown restrictions led to a substantial reduction in human movement, particularly during the 1st and 3rd wave. Despite adjustment for population density (incidence ratio (IR) = 1.985 (1.915–2.058)) and the average number of persons per room (IR = 10.411 (5.264–22.533)), we found an association between deprivation index and COVID-19 incidence (IR = 1.210 (CI: 1.077–1.357) for the most deprived quintile compared to the least deprived). There is a large range of spatial heterogeneity in COVID-19 cases in Ireland. The methods presented can be used to explore locally intensive surveillance with the possibility of localised lockdown measures to curb the transmission of infection, while keeping other, low-incidence areas open. Our results suggest that prioritising densely populated deprived areas (that are at increased risk of comorbidities) during vaccination rollout may capture people that are at risk of infection and, potentially, also those at increased risk of hospitalisation.

## 1. Introduction

Coronavirus disease 2019 (COVID-19, due to infection by severe acute respiratory syndrome coronavirus 2 (SARS-CoV-2)), which was first detected in Wuhan, China, in late 2019 has rapidly evolved from an epidemic to a pandemic within an extremely short timeframe [1]. The highly infectious disease has, as of 7 April 2021, resulted in an estimated 132 million cases and 2,875,514 deaths globally [2]. Transmission of infection mainly occurs via the respiratory route (respiratory droplets, aerosols) during close contact with an infected person, but can also occur during direct contact with contaminated surfaces. The virus can remain in aerosols for up to three hours, on cardboard up to 24 h and on plastics and stainless-steel surfaces for up to three days, with the primary method of transmission through physical contact or close proximity [3,4]. The World Health Organisation (WHO) recommends case isolation, contact tracing of individuals and quarantine, physical distancing and hygiene measures (WHO) [5]. Governments have highlighted the importance of social distancing, proximity and geographical space, at times coupled with country-wide lockdowns and restricted travel movements [6].

As human movement within and between regions is the key driver of SARS-CoV-2 infection, monitoring this is vitally important in understanding how and where the infection is spreading. Until recent years, accurately measuring and monitoring movement at a population level would have been a challenging exercise but now mobile phone geolocation data offer a reliable approach to quantify human movement which can also provide real-time information [7,8]. These data have a number of uses for government bodies, especially during a lockdown phase, including the identification of “hotspot” areas (where people are congregating), determining whether people are complying with lockdown measures, and as an epidemiological tool where these data have the potential to be used as an explanatory variable to model COVID-19 cases in space and time.

Another potentially important spatial consideration for COVID-19 is that socioeconomically deprived areas often display a higher prevalence of pre-existing health conditions and lower access to health care services. In terms of COVID-19, this may accentuate problems for deprived communities as they may be disproportionately vulnerable to infectious diseases [9]. For a national health system, and policy decision making during a pandemic, it is critically important to be aware of such disparities, if they exist. Unsurprisingly, early indications suggest that areas with the greatest socioeconomic deprivation are associated with a higher incidence of COVID-19 cases [10,11] but further investigation is warranted.

In terms of analytical approaches to track the infection, determining *R*_0_ (the reproduction number, i.e., the number of secondary infections caused by each case) through deterministic infectious disease models is usually the initial response by a country for disease surveillance at a population level [1,12,13]. This is crucial, as it provides public decision makers and authorities with evidence on whether national (or regional) control measures are effective or not. Perhaps more importantly, *R*_0_ coupled with the overall burden of infection (the number of infected people measured using the case incidence rate), providing insights into the force of infection, provides estimates on the capacity of a health system to cope with the pandemic (e.g., number of ICU beds) [14]. These models, however, primarily only include a time element and do not consider the space domain or geographical location. In addition to determining *R*_0_, disease mapping and spatial modelling are equally important epidemiological tools to monitor progression of infection across a country. In particular, advanced spatio-temporal statistical models which account for spatial and temporal autocorrelation have proven to be a fruitful exercise for modelling other similar infectious diseases including hand, foot and mouth disease [15], dengue [16] and influenza [17]. To date, however, spatio-temporal modelling of COVID-19 has been limited, as the focus has been at a country level instead of localised surveillance.

Thus, the aims of this study were to (i) map standardised incidence ratios (SIRs) of COVID-19 cases over time, (ii) present and quantify mobility trends of the Irish population during the pandemic from aggregated mobile phone data, and (iii) perform a spatio-temporal random-effects analysis, using official national COVID-19 administrative data, to examine the associations between region-specific deprivation index scores and phone metrics, and the number of confirmed cases of SARS-CoV-2 per electoral division (ED) in Ireland. This has the potential to be used by policy makers for local planning and disease surveillance.

## 2. Materials and Methods

### 2.1. Study Design and COVID-19 Data

This study utilises national Irish COVID-19 health administrative data that have been collated by several national organisations including the Department of Health, Health Services Executive (HSE) (Health Protection Surveillance Centre (HPSC), Health Intelligence Unit (HIU)), where the Central Statistics Office (CSO) has acted as data controller. Within Ireland, the first positive COVID-19 case was identified in February 2020, although the source infection for this person was never determined [18]. The initial response to the pandemic involved specialised centres being set up nationally to facilitate testing of symptomatic or suspect cases, with the first social distancing measures and movement restrictions coming into effect on March 14th followed by stricter lockdown measures on 28 March. Movement restrictions were introduced including a national stay at home order requiring people stay within 5 km of their home (except for those deemed as essential workers, e.g., medical workers). At a later stage of the pandemic when movement restrictions were lifted, localised lockdowns were introduced as discussed in the results section below.

Once an individual was confirmed with infection, the patient’s details were recorded within the administrative database. Data extracted for this study included case age, sex, and date of COVID-19 confirmation. Each case was assigned to an ED using the patient’s address at date of confirmation. EDs (n = 3409, total aggregated land mass of approximately 70,000 km^2^) are the smallest legally defined administrative areas in Ireland for which population data are available. Based on the latest CSO census (2016), we included a deprivation variable, population density measure and the average number of persons per habitable room. Deprivation indices are composite indices that combine several measures of socioeconomic status or material deprivation and here, we included the Trinity Deprivation Index [19]. This deprivation index was calculated from a principal components analysis (PCA), a common dimension reduction technique, where four separate indicators were included “unemployment”, “low social class”, “local authority rented housing” and “no car”.

Our study period included the interval from the beginning of the outbreak (1 March 2020) through to 22 February 2021. As the daily number of cases were low when examined across EDs, we aggregated cases to a weekly count per ED and merged the first two weeks of the outbreak where cases were sporadic and specialised COVID-19 administrative databases were still being created.

### 2.2. Mobile Phone Data

We obtained anonymised and aggregated ED level, data protection compliant data from one of the three main national mobile carriers in Ireland (own approximately a one-third of the total market share nationally) and extrapolated this to the full population based on the latest census data. The data included a population-level mobility metric that was calculated from the aggregation of records of mobile phone activities, formally known as the “stay-at-home index” (SHI) which was provided for each ED during the study period. SHI is an estimate of the percentage of the population travelling further than 10 km from their estimated home location. A breakdown by different age groups (<20, 20–64 (in 5-year age-bands), >64) was provided and the rolling seven-day average was extracted for each week examined. To quantify how the COVID-19 pandemic and lockdown measures impacted mobility, we calculated the percentage change of SHI with pre-COVID-19 data which we had access to (January–February 2020). Separately, a contact matrix of all ED-to-ED movements was available, which represented a count of the number of people who spent 30 min or more in one ED and then 30 min or more in another ED in the same day (i.e., journeys). For anonymity, if a cell contained five or less movements, the cell was populated with a zero. For this study, we randomly assigned a number from zero to five.

### 2.3. Statistical Analysis

#### 2.3.1. Standardised Incidence Ratio (SIR)

Crude age and sex SIRs, standardised by all cases over the study period using age and sex ED distributions from the 2016 census, were calculated and mapped by week. Defined as the ratio of observed to expected number of counts, SIRs are a crude but very useful measure of disease risk within an area. The number of expected cases of COVID-19, *E_i_* for ED *i*, represents the total number of cases that one would expect to find in that area, if it behaved like the overall standard population which can be estimated using indirect standardisation $E_{i}=\sum_{i=1:3409}r_{i}p_{i}$, where *r_i_* is the age-sex rate (COVID-19 cases/population) over the pandemic for the population, and *p_i_* is the population for ED *i*. With small populations or where the expected counts are very low, SIRs values can give misleading results [20]. Additionally, this approach does not account for spatial correlation [21]. One crude method for examining clustering is through measures of spatial autocorrelation, such as Moran’s I statistic and before we implemented a spatial model, this was calculated for deprivation index score. This statistic is similar to the common R squared value where a value of 1 indicates perfect positive correlation and −1 indicates perfect negative correlation.

#### 2.3.2. Spatio-Temporal Models

To model the counts of COVID-19 cases per ED over time, we ran several hierarchical Bayesian spatio-temporal models which can account for spatial and temporal correlations where local disease risk is modelled using a set of spatial random effects, which borrow information from neighbouring areas to get more reliable region-specific estimates, while adjustment for potential covariates can also be incorporated that results in the smoothing or shrinking of extreme values based on small sample sizes. Cases, *Y_it_* in ED *i*, for week *t*, were modelled using a negative binomial distribution in a Besag, York and Mollié (BYM) model [22]:

$$Y_{it}\;\sim \;NegBin\left(E_{i}\theta_{it}\right)$$

Spatio-temporal model:

$$\log\left(\theta_{it}\right)=\alpha +\beta_{i\prime s}+f\left(week\right)+(u_{i}+\upsilon_{i})+(\gamma_{t}+\phi_{t})+\log\left(E_{i}\right)$$

where *E_i_* can be included as an offset term in a model for standardisation purposes, and *θ_it_* which is the relative risk (RR) in ED *i* for week *t,* α denotes the intercept or overall risk in the country, *β_i,s_* the ED-specific covariates/regression parameters associated with SHI/deprivation index, *f(week)* is a smooth function of time (cubic regression spline), $u_{i}$ is a spatial correlated random effect specific to region *i* to model spatial dependence between the relative risks (RRs), and ν_i_ is an unstructured exchangeable component that models uncorrelated noise which follows a normal distribution, defined as $N\left(0,\;\sigma_{v}^{2}\right)$. $\gamma_{t}$ is smoother (rw2, random walk 2nd order) for year and φ_t_ is an extra independent and identically distributed noise term. Together, these two random-effect terms, γ_t_ and φ_t_ define the temporal term [20,23,24,25]. The BYM model assumes the spatial random effect, *u_i_*, follows a Conditional Autoregressive (CAR) distribution:

$$u_{i}|u_{-i}\;\sim \;N\left(\sum_{j=1}^{N}c_{ij}u_{j},\;\sigma^{2}\right)$$

where *c_i_*_j_ is an adjacency matrix, with *c_ij_* = 1 if area *i* and *j* are neighbours and 0 otherwise (queen’s adjacency). This is a conditional distribution, where the $u_{i}|u_{-i}$ $u_{i}|u_{-i}$ notation indicates that we are modelling the distribution of the random-effect *u_i_* given all the other *u_i_*s, except *u_i_* itself. Hence, the conditional mean of the random effect for ED *i*, *u_i_* given all the other random effects, is the average of the neighbouring ED random effects. In this BYM model, smoothed SIRs can be considered the posterior mean of the RR [26].

#### 2.3.3. Model Fitting

In a traditional maximum likelihood estimation (MLE) framework, convergence issues can often be a problem when attempting to solve spatial models. For this reason, Bayesian approaches were implemented to fit models. As there were a large number of EDs with a high number of time-points, computation time was an important consideration and for this reason, models were fitted using the Integrated nested Laplace approximation (INLA) approach of Rue et al. [27]. Vague prior densities were assigned to the fixed parameters: normal distribution with mean 0 and precision  τ=0.001. As $\tau =1/\sigma^{2}$, this translates to σ=31.6, so the fixed parameter priors are of the form $\beta \;\sim \;N\left(0,\;31.6^{2}\right)$. Similarly, for the variance parameters or hyperparameters; $\tau_{u}$, $\tau_{v}$, $\tau_{\gamma }$ and $\tau_{\phi }$ we chose vague priors which all followed: ~LogGamma(1, 0.00005). Regression estimates are presented as the means and 95% credible intervals. All analysis and mapping were conducted using R (R Core Team, 2020). INLA models were fitted using the *R-INLA* package [28].

#### 2.3.4. Model Selection

Alternative models were compared to choose the one best describing the data. This process involved fitting different distributions to our count outcome (Poisson, negative binomial) and formally comparing models using the deviance information criterion (DIC) and widely applicable information criterion (WAIC), where lower values indicate better fit [21,24]. Additionally, leave-one-out cross-validation predictive checks were also implemented where two quantities, conditional predictive ordinate (CPO) and probability integral transform (PIT), were used for evaluating the goodness of fit for these Bayesian models [29]. Uniformity of PIT values indicate that the predictive distributions match the observations, suggesting a well-fitted model. The product of all the CPO values can be considered a pseudo marginal likelihood, which gives a cross-validatory summary measure of fit. The log pseudo marginal likelihood (LPML) is the log of this measure and is often used as an alternative measure for DIC. Unlike DIC, however, high LPML values suggest the model is better supported by the data [30].

## 3. Results

**Figure 1:**
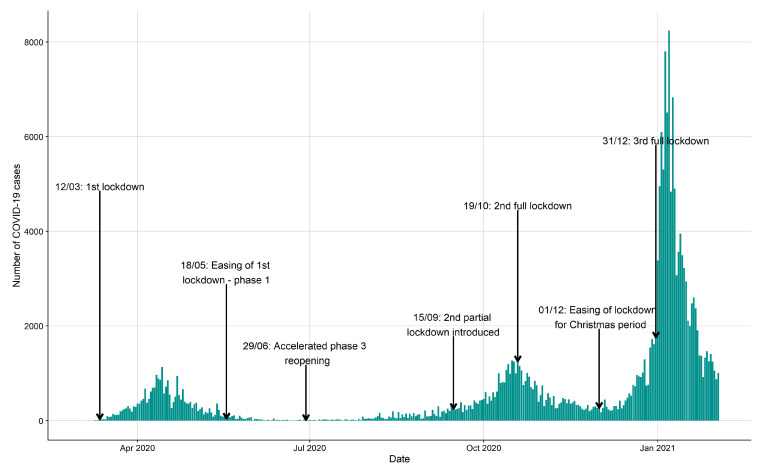
Distribution of all COVID-19 cases over time from the start of the pandemic in Ireland along with key lockdown dates. Three distinct waves are evident.

As of 22 February 2021, there were 216,274 number of COVID-19 cases in Ireland. Of these, 214,021 cases (98.9%) had addresses that were able to be successfully geocoded and assigned to an ED. The distribution of the overall incidence of new cases in Ireland over time is presented in Figure 1 along with some key lockdown dates introduced during the pandemic in Ireland. It is evident that there have been three “waves” of COVID-19 cases and a breakdown of the main characteristics of these patients by each wave is presented in Table 1. Wave 3, the most recent, has been the most severe to date with the majority of all new cases during the pandemic occurring during this period (63%). During wave 1, the largest proportion of cases were in the 40−59 year age band (34.3 %), but during wave 2 and 3, this shifted to the 20−39 year age band (37.5 % and 37.9 %, respectively). Wave 1 had the highest number of COVID cases over 80 year (14.4% of all cases), while wave 2 and 3 had substantially lower numbers of the total cases in those periods (only representing 3.3% and 4.7% of all cases in each wave, respectively). The sex distribution between waves has been relatively equal, although there have been more females than males in each wave.

**Figure 2:**
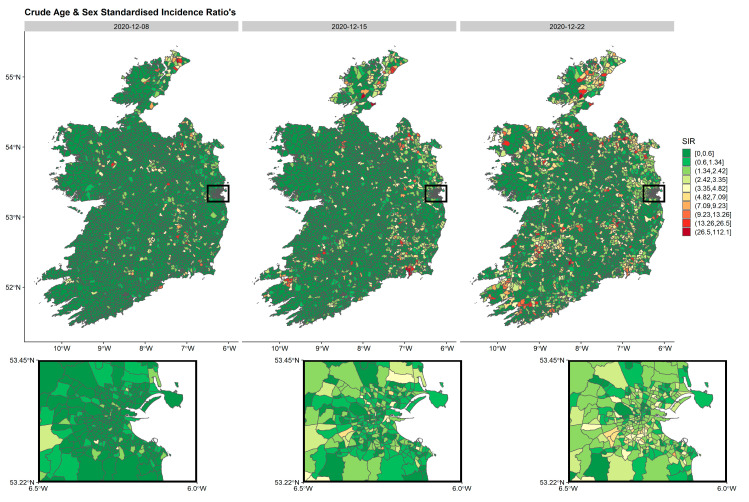
Age- and sex-standardised incidence ratios’ (SIRs) by week per ED in Ireland during the first three weeks of wave 3 (8 December 2020–22 December 2020). The inset represents the greater Dublin area (capital of Ireland), where EDs are generally smaller in geographical size due to larger populations. The maps are standardised to the whole pandemic.

**Figure 3:**
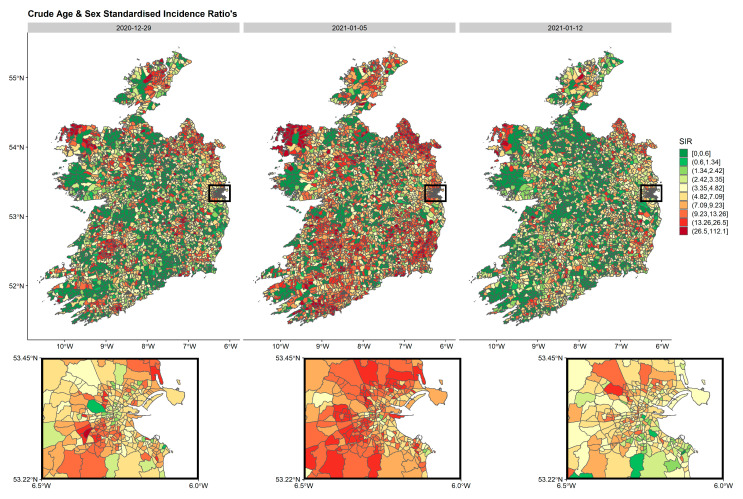
Age- and sex-standardised incidence ratios’ (SIRs) by week per ED in Ireland during the second three weeks of wave 3 (29 December 2020–12 January 2021). The inset represents the greater Dublin area (capital of Ireland), where EDs are generally smaller in geographical size due to larger populations. The maps are standardised to the whole pandemic.

**Figure 4:**
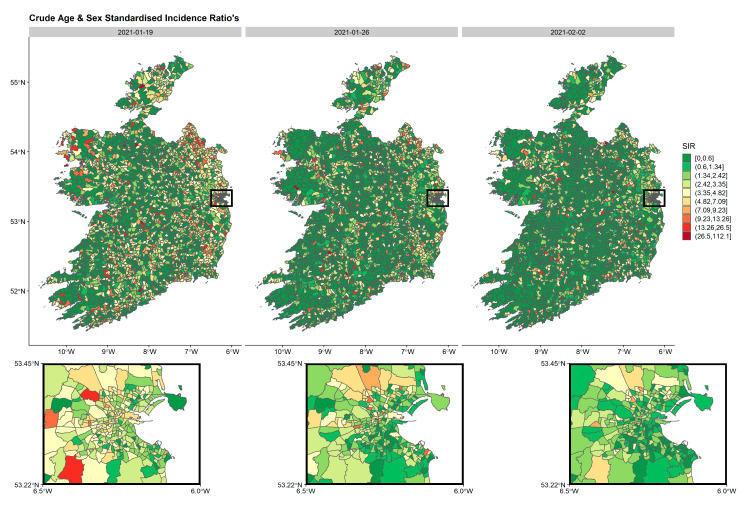
Age- and sex-standardised incidence ratio’s (SIRs) by week per ED in Ireland during the third three weeks of wave 3 (19 January 2021–2 February 2021). The inset represents the greater Dublin area (capital of Ireland), where EDs are generally smaller in geographical size due to larger populations. The maps are standardised to the whole pandemic.

The spatial distribution of crude age and sex SIRs, standardised by all cases over the study period using age and sex ED distributions from the 2016 census were calculated and mapped by week. For practical reasons, only weeks during wave 3 (Figure 2, Figure 3 and Figure 4) are presented in the manuscript although all weeks are included in the supplementary material (SM1). These spatio-temporal SIR plots highlight the explosion of cases observed during wave 3. It is clear, that high-incidence EDs are clustered together with some areas having very low incidence rates.

**Figure 5:**
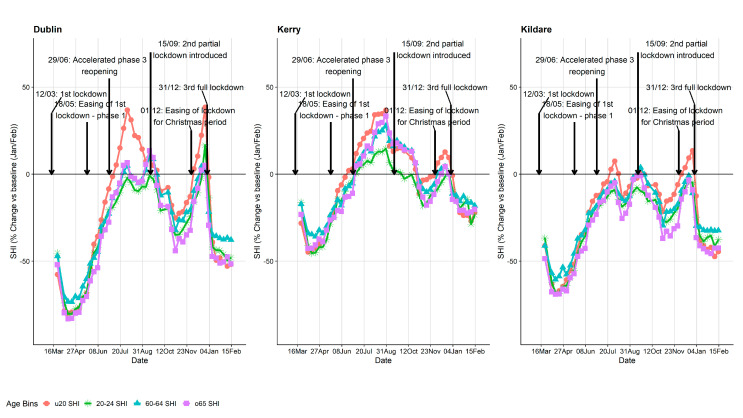
Mobility plot—percentage change in SHI compared to their baseline figures (January/February 2020) for counties Dublin, Kerry and Kildare by the two youngest and oldest age groups (for clarity). Plots of all 26 Irish counties are included in the supplementary material.

**Figure 6:**
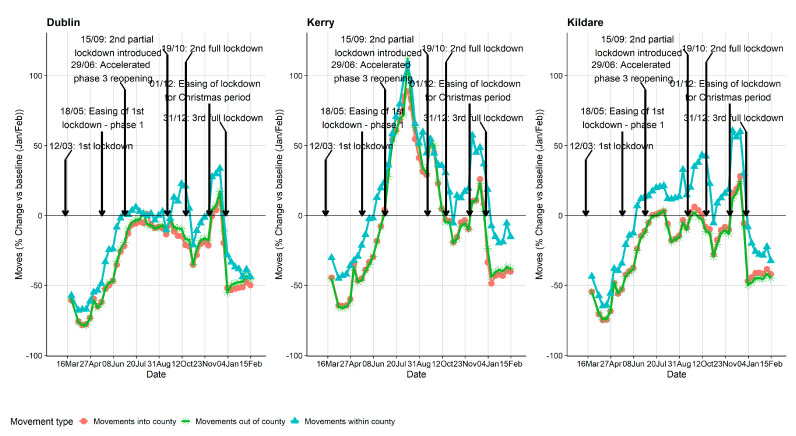
Mobility plot—percentage change in the number of journeys into, out of and within counties Dublin, Kerry and Kildare compared to their baseline figures (January/February 2020). Plots of all 26 Irish counties are included in the supplementary material.

Mobility trend plots are presented in Figure 5 and Figure 6. Although we have ED-level data, for the purpose of visualisations, we believe it was more informative to present county level data (larger spatial resolution). Again, for practical reasons, only a subset of counties are presented in the manuscript (see supplementary material, SM2 and SM3, for all county plots). The three counties presented were chosen intentionally as they represent quite different counties in terms of the pandemic and geographical setting. Dublin is the capital city of Ireland and has the highest population density, Kerry (south-west of Ireland) is more rural and is a very popular tourist destination particularly during the summer months, and Kildare (immediately to the southwest of Dublin) was chosen as it was a county that went into a localised lockdown at the very early stages of wave 2 before the wave 2 country-wide lockdown came into effect. The SHI metric by age groups is presented in Figure 5 where a similar pattern is broadly seen for all counties: a sharp reduction during wave 1 followed by a large increase during the summer months with another reduction during wave 2 and 3. A maximum reduction of up to 83% was observed during wave 1 in Dublin for those aged >64 y. For Kildare, a reduction in movement is observed during August which coincides with their localised lockdown before the country-wide restrictions were introduced. Similarly, the percentage change in the number of journeys is presented in Figure 6. The trends are similar to the SHI plots except for county Kerry, which had a large increase in the number of journeys into, out of, and within the county during the summer months compared to baseline. Importantly, however, the number of COVID-19 cases in Kerry during May, June, July and August was only 42. The overall correlation between the number of COVID-19 cases and percentage change of SHI was −0.135.

Negative binomial models were a better fit to the data compared to one that assumed a Poisson distribution (results not shown). Different spatio-temporal models assuming a negative binomial distribution were fit to the data and their associated measures of fit (DIC, WAIC, LPML) are compared in Table 2. Unsurprisingly, based on the metrics in Table 2, the best fit to the data was a model that included all variables. However, it is evident that both SHI and deprivation score only moderately improve model fit. The posterior means and 95% credible intervals (CIs) for the parameters from three models that all include cubic time spline terms and: deprivation quintiles (Model 1); deprivation quintiles, population density, persons per room (Model 2); full model including the addition of SHI (Model 3), are presented in Table 3. Without adjustment for population density and the average number of persons per room, there was a strong association between the most deprived quintile compared to the least deprived (incidence ratio (IR) = 3.819 (3.406–4.281), Model 1). Despite further adjustment for these variables, we still found an association between the most deprived quintile compared to the least and COVID-19 incidence (IR = 1.210 (CI: 1.077–1.357), Model 2). Unsurprisingly, population density (IR = 1.985 (1.915–2.058)) and persons per room (IR = 10.411 (5.264–22.533)) were also associated with increased COVID-19 incidence in this model (Model 2). An increase in mobility, as measured as the percentage change in SHI was negatively associated with incidence (IR = 0.879 (0.875–0.883), Model 3) although we urge caution when interpreting this finding as examined in the Discussion below, where we advocate for Model 2 as our final model (model without mobility metrics). Similar results were found when we lagged SHI by a week to allow for the delay between date of infection and detection. The Moran’s I value for deprivation score was 0.47 indicating a positive correlation with deprived regions tend to be closer together and further from less deprived regions.

**Figure 7:**
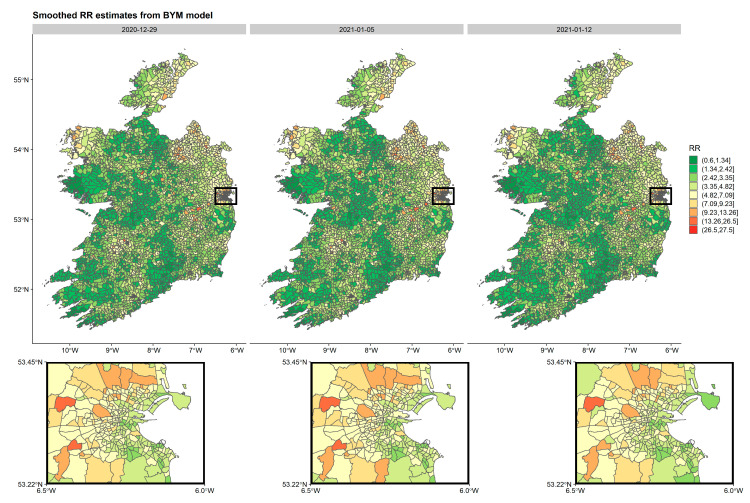
Smoothed relative risks (RR) from spatio-temporal BYM Model 2 by week per ED in Ireland during the second three weeks of wave 3 (29 December 2020–12 January 2021). The inset represents the greater Dublin area (capital city of Ireland), where EDs are generally smaller in geographical size due to larger populations.

In Model 2, the posterior mean of $\sigma_{u_{i}}$ is 0.861, meaning that the majority of the spatially correlated random-effects $u_{i}$ lie between −1.099 and 2.821 (0.861 ± 1.96), while the additional noise term $\sigma_{v_{i}}$ is only 0.466, suggesting that it is contributing much less to the model than $\sigma_{u_{i}}$. The resulting RRs from this BYM model for COVID-19 incidence were mapped for Ireland (see Figure 7 for three separate weeks, see Supplementary Materials (SM4) for all weeks). These are equivalent to the crude SIR values but have been smoothed to account for spatio-temporal correlations and deprivation score with the aim of eliminating random noise. These maps strongly correlate with the crude SIR maps but note, the smoothed estimates have resulted in shrunken estimates compared to the SIR estimates.

## 4. Discussion

In this study, we have mapped SIRs of COVID-19 cases over time and presented mobility trends of the Irish population during the pandemic to date, based on aggregated phone activity. We also implemented hierarchical Bayesian spatio-temporal regression models using random effects to explore the association between deprivation index, population density measures, mobility, and COVID-19 incidence over time at a small spatial resolution. So far, the pandemic has been characterised by three distinct waves. We found that lockdown restrictions led to a substantial reduction in human movement as measured by mobility metrics. We found an association between both densely populated and deprived areas, and COVID-19 incidence.

It is unsurprising that wave 3 has been the most severe wave to date which occurred during December 2020 and January 2021. Unfortunately, this period coincided with several key factors that facilitated the rapid spread of infection. As the country was returning to lower incidence rates from wave 2, but still with a not insignificant burden of infection (5 day average rolling case numbers of 275 on 2nd December [31]), the decision was made to begin the easing of lockdown restrictions. At the beginning of December, the opening of all retail stores, restaurants, bars and cafés was permitted. Importantly, this also coincided with the Christmas holiday period, where traditionally the number of social gatherings of friends and family increases greatly. In addition, as people travelled home from within Ireland from abroad, for the holiday period, it facilitated mixing of individuals from different generations. Particularly, it meant that older age groups who would usually be less risk averse in terms of social distancing, etc., would be meeting younger age groups where incidence was highest. Similar experiences have been observed in USA, Israel, China and elsewhere, which demonstrated that festive holidays, which tend to draw individuals from distant places into close contact for prolonged periods, are associated with a spike in COVID-19 incidence [32,33,34]. This naturally increases the chance of introduction of new strains, e.g., B.1.1.7 [35].

As social mixing and mobility are the key drivers for the transmission of infection, it is important to attempt to monitor them during a pandemic [4]. Mobile phone records are recognised as the gold standard for measuring human movement [8,36] and the pandemic has seen a surge in interest in them, primarily by state agencies, to monitor physical distancing and to determine if the public are adhering to movement restrictions [37]. Overall, we found large reductions in the mobility metrics which coincided with state lockdown measures. However, despite mobile phone records being the gold standard for measuring mobility, for anonymity reasons, we are restricted to using aggregated data at ED or county level. Despite ED-level data being at a very high spatial resolution, it can mask localised gathering where mixing could occur, e.g., attendance at a large funeral or a local sports event. One of the critical aspects to remember when monitoring mobility movement in an infectious disease setting is that it is not possible to untangle how human behaviour has changed over time. For example, when movement returned to normal levels during the summer months after wave 1, a sudden surge in cases in Ireland was not observed, suggesting that mobility, as measured by aggregated phone records, is not on the causal pathway. A good example of this can be seen with the large increase in movements associated with County Kerry during the summer months representing a “staycation” effect whereby the public were holidaying within Ireland instead of traveling abroad, but this did not result in an increase in COVID-19 cases in the local area (or in Ireland overall). We hypothesis that the primary reason for this is that human behaviour had changed whereby the actions of individuals were more conscientious with respect to public health guidelines (e.g., wearing face masks, hand washing and covering sneezes and coughs). Gatalo et al. [38] have also recognised this limitation where they suggest that mobile phone mobility data only captured a small component of the behaviours associated with social distancing that reduced transmission of SARS-CoV-2 in the early stages of the pandemic in the USA. The absence of a strong correlation between mobility and case growth after the initial phase of the pandemic (when a longer study period was analysed) suggests that other individual level factors, such as wearing a mask or maintaining distance even when encountering individuals, are likely to be more important than mobility alone. They also suggest that overdispersion identified in the distribution of transmissions suggests that a small number of individuals are likely to account for a large proportion of transmission. Separately, despite being recognised as the gold standard for measuring human movement, the accuracy of mobile phone records themselves has been raised and variation in usage has been seen across subpopulations, leading to concerns about coverage bias, particularly in older age groups [39]. Furthermore, this may lead to ascertainment bias where certain individuals or groups may be excluded from the data. Meng has previously highlighted that “big data” does not necessarily equate to a representative sample of the population [40]. We do not have access to the precise demographic breakdown of the mobile carriers’ users and hence, we cannot determine how representative the sample is of the whole population in this study. With all this in mind, we advise caution when interpreting the findings from our regression models that includes mobility metrics. It is precisely for these concerns that the final model for which we advocate is one without mobility metrics. Nonetheless, mobility data remain a critical tool for state and public agencies during a pandemic to monitor adherence to lockdown restrictions. In addition, the data can play an important role in infectious disease models (for scenario modelling for R_0_, the reproduction number), where the data can be used to get a proxy for social mixing [36].

Our finding that higher deprivation index was associated with a higher incidence of COVID-19 is in line with emerging research from other spatial regression studies. Whittle and Diaz-Artiles [11], who conducted a similar analysis (also using a BYM INLA model), found that socioeconomic factors helped to explain disparities in incidence levels between neighbourhoods in New York City. They found that densely populated, low-income, and predominantly black neighbourhoods were strongly associated with COVID-19 test positivity. Likewise, in Brazil, Campos de Lima et al. [10] also implemented spatial regression models using INLA and found that COVID-19 infections were spatially distributed, forming clusters and hotspots. Less developed areas with lower socioeconomic status were associated with increased risk of infection. In our analysis, we used a deprivation score [19] derived through PCA that is comprised of four separate socioeconomic markers (“unemployment”, “low social class”, “local authority rented housing” and “no car”). We believe this is a more robust measure of socioeconomic status rather than just focusing on mean/median salary in an area. A large proportion of Ireland is rural, and salary, which is often used as a proxy for socioeconomic status, may biases heavily against rural areas (where the cost of living may be very different, so a lower salary does not necessarily imply lower socioeconomic status). The interesting aspect of our findings is that we still observed an association with deprivation index despite additional adjustment for the number of persons per room and population density. By its nature, an infectious disease requires close contact between hosts for transmission and we have shown that despite adjustment for this, deprived areas are still at an increased risk of incidence. People living in cramped conditions cannot effectively self-isolate and may not have the means to do anything about it, suggesting that a vaccination programme should consider prioritising these individuals.

As previously alluded to, there has been limited application of spatio-temporal statistical models on COVID-19 data. This is despite a pressing need for them for to inform local response strategies as recognised by WHO [5]. Visualising the spread of infection in a country is of critical importance for prioritizing policy and rapid decision making. Disease mapping is most useful towards the extremes of a wave (e.g., at the beginning of a wave or towards the end when cases begin to fall) when cases are not widespread geographically. In Ireland, this was evident in the summer months in counties Laois, Offaly and Kildare, when incidence was low nationally, but cases began to climb in these counties. The government, with the aid of mapping tools along with infectious disease models, decided to introduce localised lockdown measures for these counties while keeping the rest of the country open. Advanced statistical spatio-temporal methods have progressed significantly in the last 15 years particularly with the seminal paper by Rue et al. [27] who developed the INLA approach. Similar to our approach, these models have been used successfully in other countries to monitor the spread of SAR-CoV-2 infection at a spatio-temporal level [10,11,41].

Our current study has several strengths. To the best of our knowledge, this is the first spatial study to explore the association between deprivation index and incidence of COVID-19 while adjusting for population density and the average number of persons per room. This is the first study that we are aware of to explore the spatio-temporal spread of COVID-19 in Ireland where we had close to full geocoding of all confirmed cases. Access to high-quality, reliable, anonymised phone data from one of the largest phone carriers in Ireland is a big strength of this work which has resulted in accurate human movement metrics. Within the Irish setting, identification of socioeconomic status as a risk marker is a novel and valuable finding. Identifying vulnerable groups ahead of time may help in preparedness and response policies if future waves materialise. This study also has several limitations. Given that the pandemic is ongoing, we rely on data that are incomplete, with systematic biases (in reporting of symptoms, testing, etc.), and subject to future consolidation as has been acknowledged in other large studies [42]. In Ireland, the address and hence ED location of tested individuals was poorly recorded as part of the public health response. Ireland introduced a postal code system in recent years whereby each house has a separate individual code which is perfect for geocoding but to date, households do not know their code verbatim. Adjusting for the underlying number of people requesting tests (a proxy for symptomatic cases) would be desired as it would be a potentially important predictor of the future number of cases. However, the inclusion of the testing data did not improve model fit (data not shown) and we suspect that there was a strong reporting bias, where correct recording of addresses was influenced by the conscientiousness of the administrative inputter which would naturally vary across the country. We did not have access to Northern Ireland COVID-19 data, which may have implications for estimates from Irish border counties.

## 5. Conclusions

In conclusion, we found an association between population density, the average number of persons per room and deprivation index, and COVID-19 incidence. Our results suggest that prioritising densely populated, deprived areas (that are at increased risk of comorbidities) during vaccination rollout may capture people that are at risk of infection and, potentially, also those at increased risk of hospitalisation. Results and the methodology presented here may be useful for preparing for future pandemics, which may help to minimize the health and economic damage sustained by countries. Managing epidemics requires facing up to inequalities and ensuring supports are put in place to protect those most at risk.

## Figures and Tables

**Table 1:** The number of cases in each wave of the pandemic in Ireland with a breakdown by age and sex.

Variable		Wave 1 2 March 2020–31 May 2020	Wave 2 1 June 2020–30 November 2020	Wave 3 1 December 2020–22 February 2021
Total N (%)		24,957 (12.6)	47,729 (24.1)	125,586 (63.3)
Sex	f	14,287 (57.2)	24,131 (50.6)	66,082 (52.6)
	m	10,670 (42.8)	23,598 (49.4)	59,504 (47.4)
Age	0−19	899 (3.6)	10,655 (22.3)	17,783 (14.2)
	20−39	7761 (31.1)	17,922 (37.5)	47,659 (37.9)
	40−59	8564 (34.3)	12,524 (26.2)	38,196 (30.4)
	60−79	4146 (16.6)	5072 (10.6)	16,028 (12.8)
	80+	3587 (14.4)	1556 (3.3)	5920 (4.7)

% for “Total” row is row-wise.

**Table 2:** Comparison of spatio-temporal negative binomial BYM models.

Model	DIC	WAIC	LPML	Dispersion Statistic
Deprivation quintile	337,975	338,798	−169,485	1.02
Log population density	337,541	338,307	−169,224	1.06
SHI % change	334,401	335,148	−167,659	0.98
Cubic spline (week)	285,226	286,153	−143,171	1.13
Persons per room	337,944	338,776	−169,478	1.01
Deprivation quintile + log population density + SHI % change	333,869	334,537	−167,329	1.03
Deprivation quintile + log population density + cubic spline (week)	284,776	285,672	−142,918	1.16
Deprivation quintile + SHI % change + cubic spline (week)	281,942	282,899	−141,538	1.15
Log population density + SHI % change + cubic spline (week)	281,604	282,552	−141,357	1.16
Deprivation quintile + log population density + SHI % change + cubic spline (week)	281,595	282,537	−141,348	1.16
Deprivation quintile + log population density + persons per room + cubic spline (week)	284,764	285,658	−142,911	1.16
Deprivation quintile + log population density + persons per room + SHI % change + cubic spline (week)	281,583	282,523	−141,340	1.16

BYM: Besag, York, and Mollié model; DIC: deviance information criterion; WAIC: widely available information criterion; LPML: log pseudo marginal likelihood; cubic spline: cubic regression spline (8 knots).

**Table 3:** Posterior means and 95% credible intervals from negative binomial BYM models.

	Model 1		Model 2		Model 3	
Variable	Mean	95% Credible Interval	Mean	95% Credible Interval	Mean	95% Credible Interval
Deprivation quintile 2	1.171	1.060–1.294	0.970	0.891–1.056	0.973	0.894–1.059
Deprivation quintile 3	1.360	1.225–1.510	1.025	0.937–1.121	1.044	0.955–1.142
Deprivation quintile 4	1.931	1.737–2.147	1.159	1.056–1.272	1.157	1.055–1.269
Deprivation quintile 5	3.819	3.406–4.281	1.210	1.077–1.357	1.262	1.126–1.414
Log population density			1.985	1.915–2.058	1.926	1.860–1.996
Person per room			10.411	5.264–22.533	11.822	5.954–26.231
SHI% change (per 5)					0.879	0.875–0.883
Spline term 1	2.207	2.100–2.320	2.220	2.113–2.333	1.084	1.025–1.145
Spline term 2	0.061	0.058–0.066	0.061	0.057–0.066	0.078	0.073–0.084
Spline term 3	0.124	0.117–0.132	0.124	0.117–0.132	0.215	0.202–0.228
Spline term 4	3.398	3.265–3.536	3.403	3.270–3.542	5.245	5.032–5.467
Spline term 5	2.254	2.172–2.339	2.259	2.176–2.344	2.903	2.797–3.014
Spline term 6	14.805	14.282–15.349	14.826	14.301–15.371	16.088	15.525–16.672
Spline term 7	5.915	5.657–6.186	5.907	5.649–6.178	3.942	3.769–4.123
						
Spatial hyperparameters						
σ_ui_	1.162	1.081–250	0.861	0.804–0.922	0.815	0.754–0.878
σ_vi_	0.529	0.477–0.580	0.466	0.429–0.500	0.483	0.448–0.517

## Data Availability

The data that support the findings of this study are available from the Irish Central Statistics Office, but restrictions apply to the availability of these data, which were accessed by the primary author for the purpose of these analyses and under Section 20(b) of the Statistics Act, 1993 for the purpose of using data collected during the COVID-19 pandemic to aid in the national response.

## Supplementary Materials

The following are available online at https://www.mdpi.com/article/10.3390/ijerph18126285/s1, Figure S1: SM1_standardise_per_week_inset_04_Mar_2021. Figure S2: SM2_county_shi_age_all_counties23_Feb_2021. Figure S3: SM3_county_CM_age_all_counties08_Mar_2021. Figure S4: SM4_RR_per_week_inset_05_Mar_2021.
