# Supplementary material for: Population Mobility Trends, Deprivation Index and the Spatio-Temporal Spread of Coronavirus Disease 2019 in Ireland
Source: Int J Environ Res Public Health. 2021 Jun 10;18(12):6285. doi: 10.3390/ijerph18126285 (PMC8296107; doi:10.3390/ijerph18126285)
Supplement: Supplementary file 1 [file ijerph-18-06285-s001.zip › SM2_county_shi_age_all_counties23_Feb_2021.pdf]

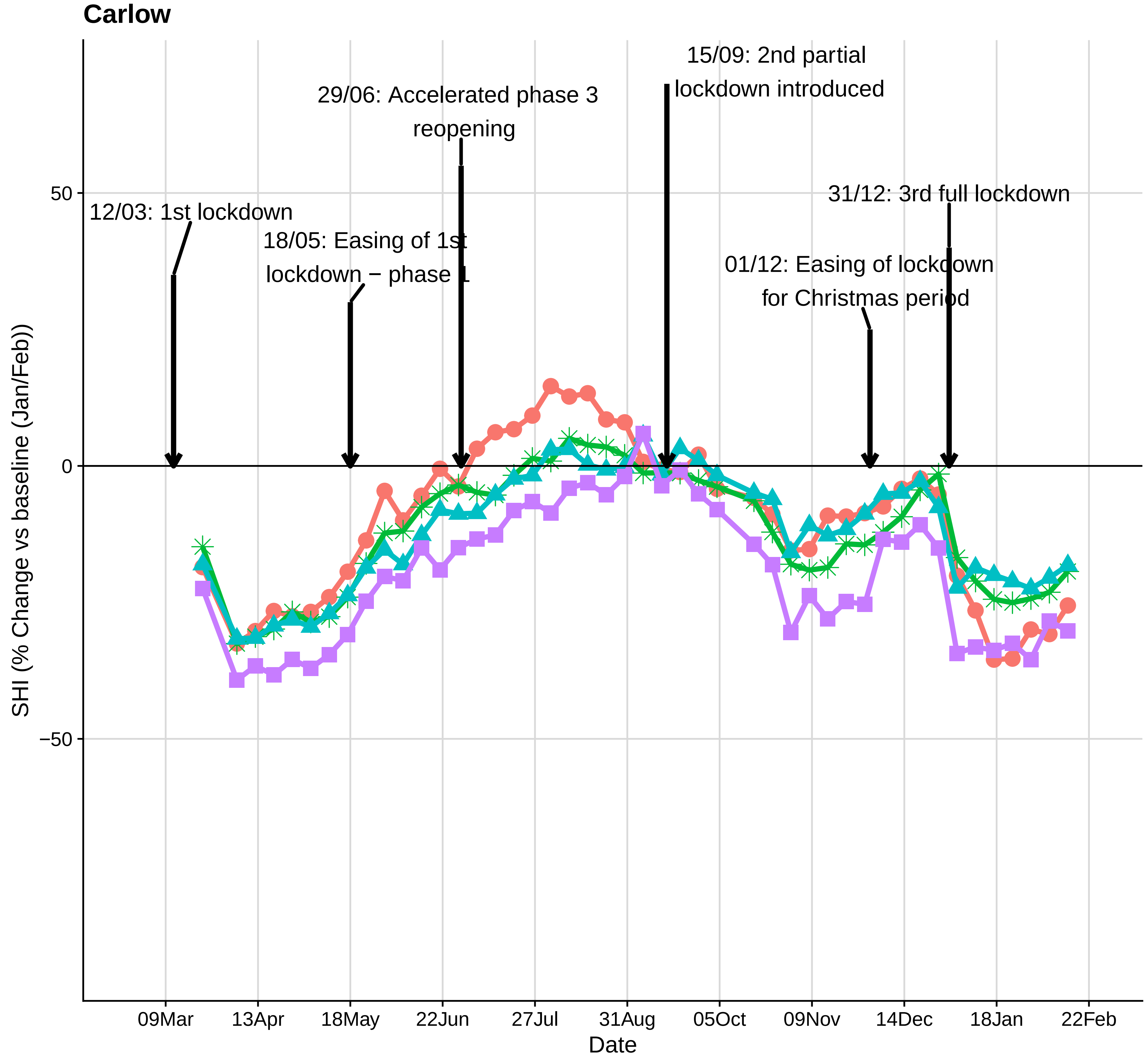

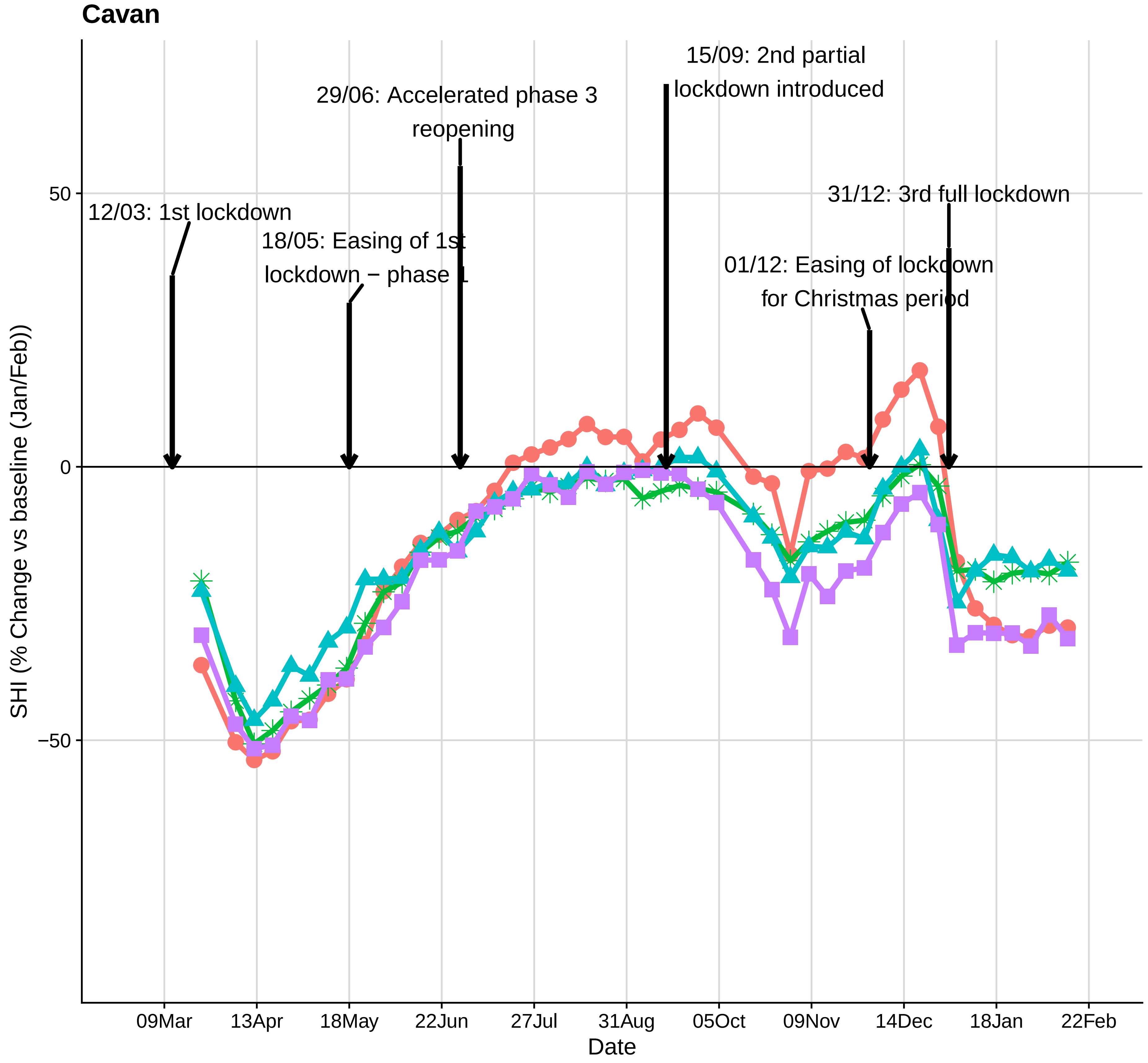

Age Bins u20 SHI 20−24 SHI 60−64 SHI o65 SHI

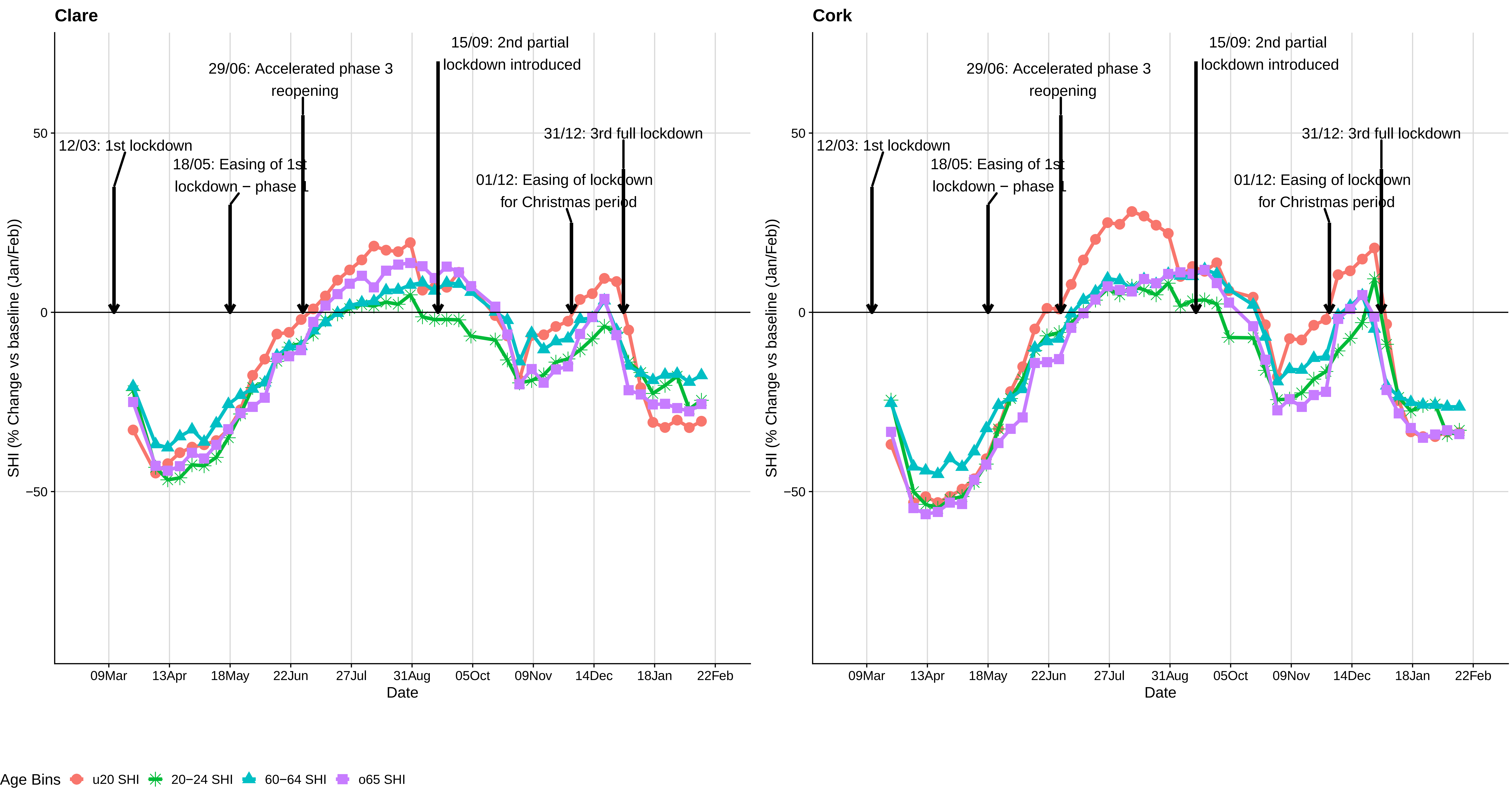
Clare
Cork
15/09: 2nd partial lockdown introduced
29/06: Accelerated phase 3 reopening
12/03: 1st lockdown
18/05: Easing of 1st lockdown − phase 1
31/12: 3rd full lockdown
01/12: Easing of lockdown for Christmas period
SHI (% Change vs baseline (Jan/Feb))
50
0
−50
09Mar
13Apr
18May
22Jun
27Jul
31Aug
05Oct
09Nov
14Dec
18Jan
22Feb
Date
Age Bins
u20 SHI
20−24 SHI
60−64 SHI
o65 SHI

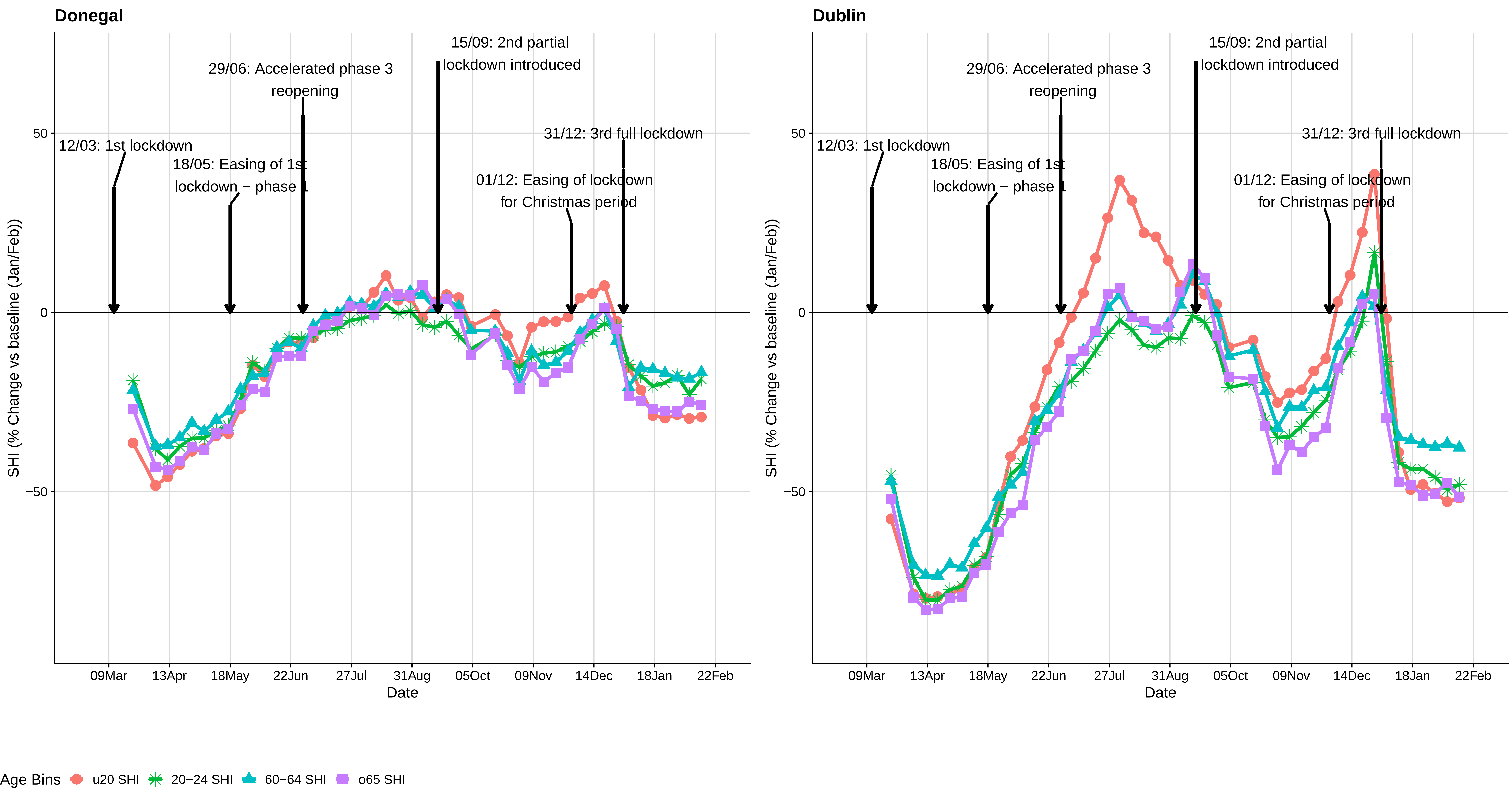
Donegal
Dublin
12/03: 1st lockdown
18/05: Easing of 1st lockdown − phase 1
29/06: Accelerated phase 3 reopening
15/09: 2nd partial lockdown introduced
01/12: Easing of lockdown for Christmas period
31/12: 3rd full lockdown
SHI (% Change vs baseline (Jan/Feb))
50
0
−50
09Mar
13Apr
18May
22Jun
27Jul
31Aug
05Oct
09Nov
14Dec
18Jan
22Feb
Date
Age Bins
u20 SHI
20−24 SHI
60−64 SHI
o65 SHI

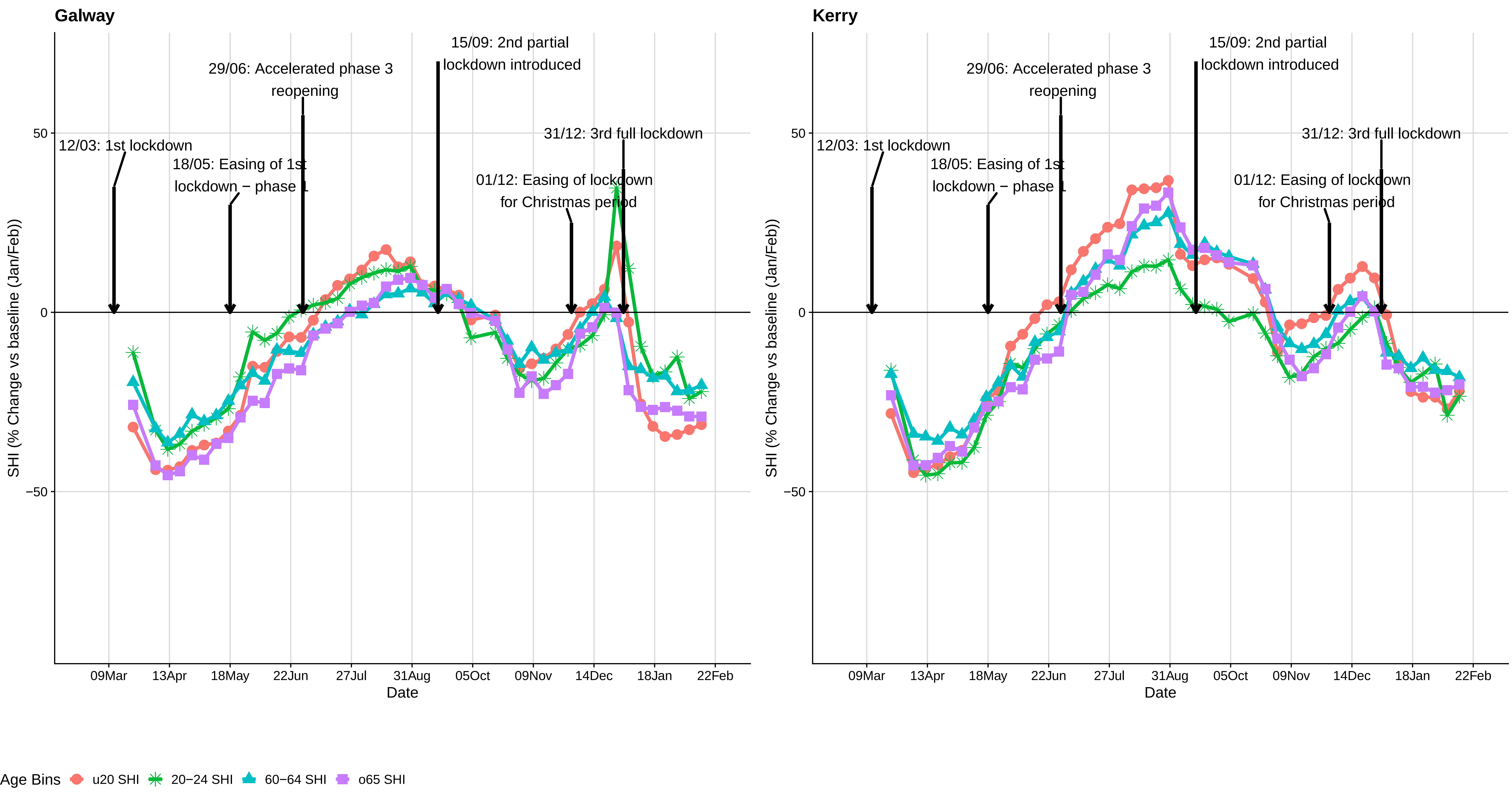
Galway
Kerry
15/09: 2nd partial lockdown introduced
29/06: Accelerated phase 3 reopening
31/12: 3rd full lockdown
12/03: 1st lockdown
18/05: Easing of 1st lockdown − phase 1
01/12: Easing of lockdown for Christmas period
SHI (% Change vs baseline (Jan/Feb))
50
0
−50
09Mar
13Apr
18May
22Jun
27Jul
31Aug
05Oct
09Nov
14Dec
18Jan
22Feb
Date
Age Bins
u20 SHI
20−24 SHI
60−64 SHI
o65 SHI

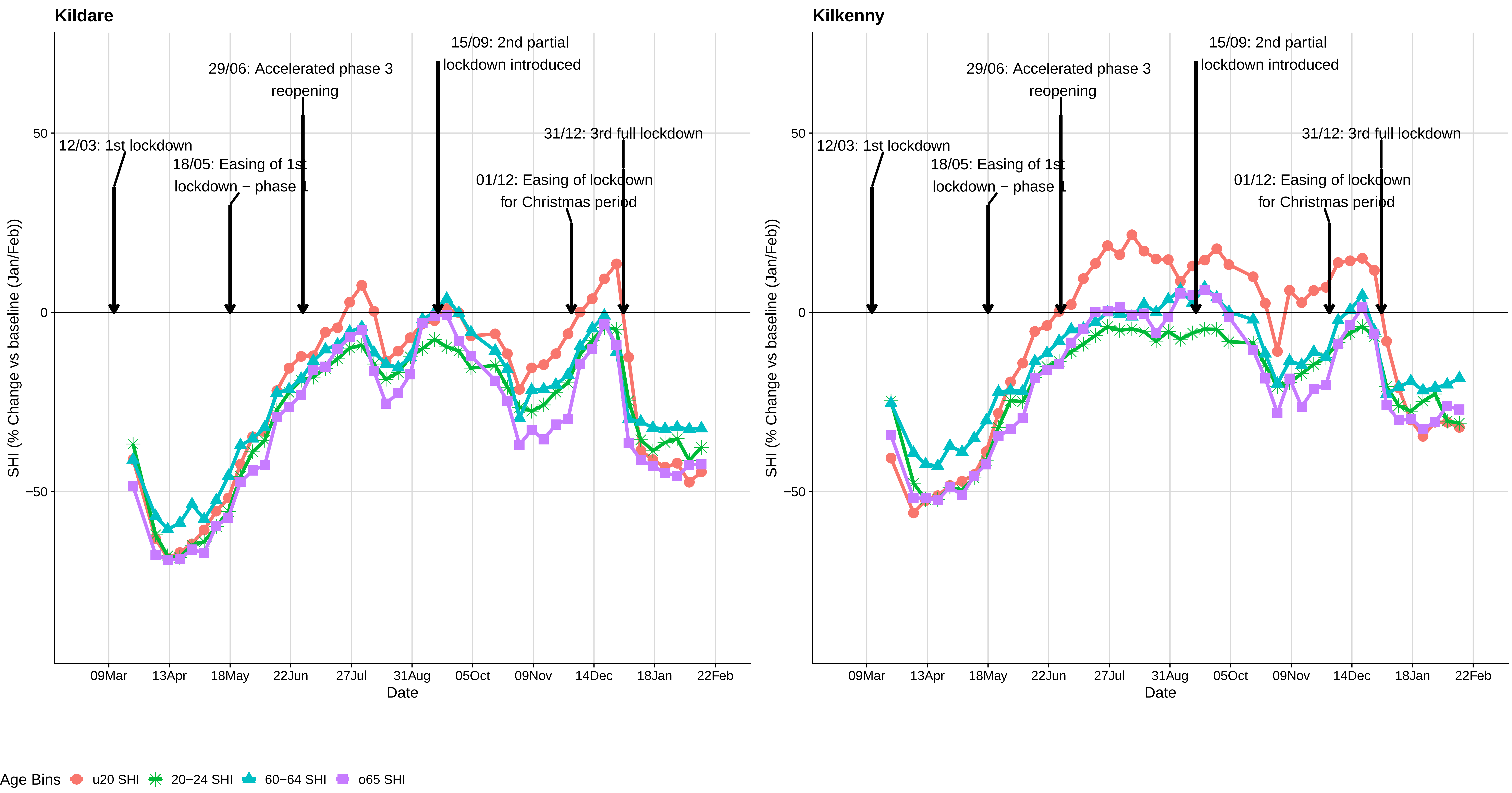
Kildare
12/03: 1st lockdown
18/05: Easing of 1st lockdown − phase 1
29/06: Accelerated phase 3 reopening
15/09: 2nd partial lockdown introduced
01/12: Easing of lockdown for Christmas period
31/12: 3rd full lockdown
SHI (% Change vs baseline (Jan/Feb))
50
0
−50
09Mar
13Apr
18May
22Jun
27Jul
31Aug
05Oct
09Nov
14Dec
18Jan
22Feb
Date
Kilkenny
12/03: 1st lockdown
18/05: Easing of 1st lockdown − phase 1
29/06: Accelerated phase 3 reopening
15/09: 2nd partial lockdown introduced
01/12: Easing of lockdown for Christmas period
31/12: 3rd full lockdown
SHI (% Change vs baseline (Jan/Feb))
50
0
−50
09Mar
13Apr
18May
22Jun
27Jul
31Aug
05Oct
09Nov
14Dec
18Jan
22Feb
Date
Age Bins
u20 SHI
20−24 SHI
60−64 SHI
o65 SHI

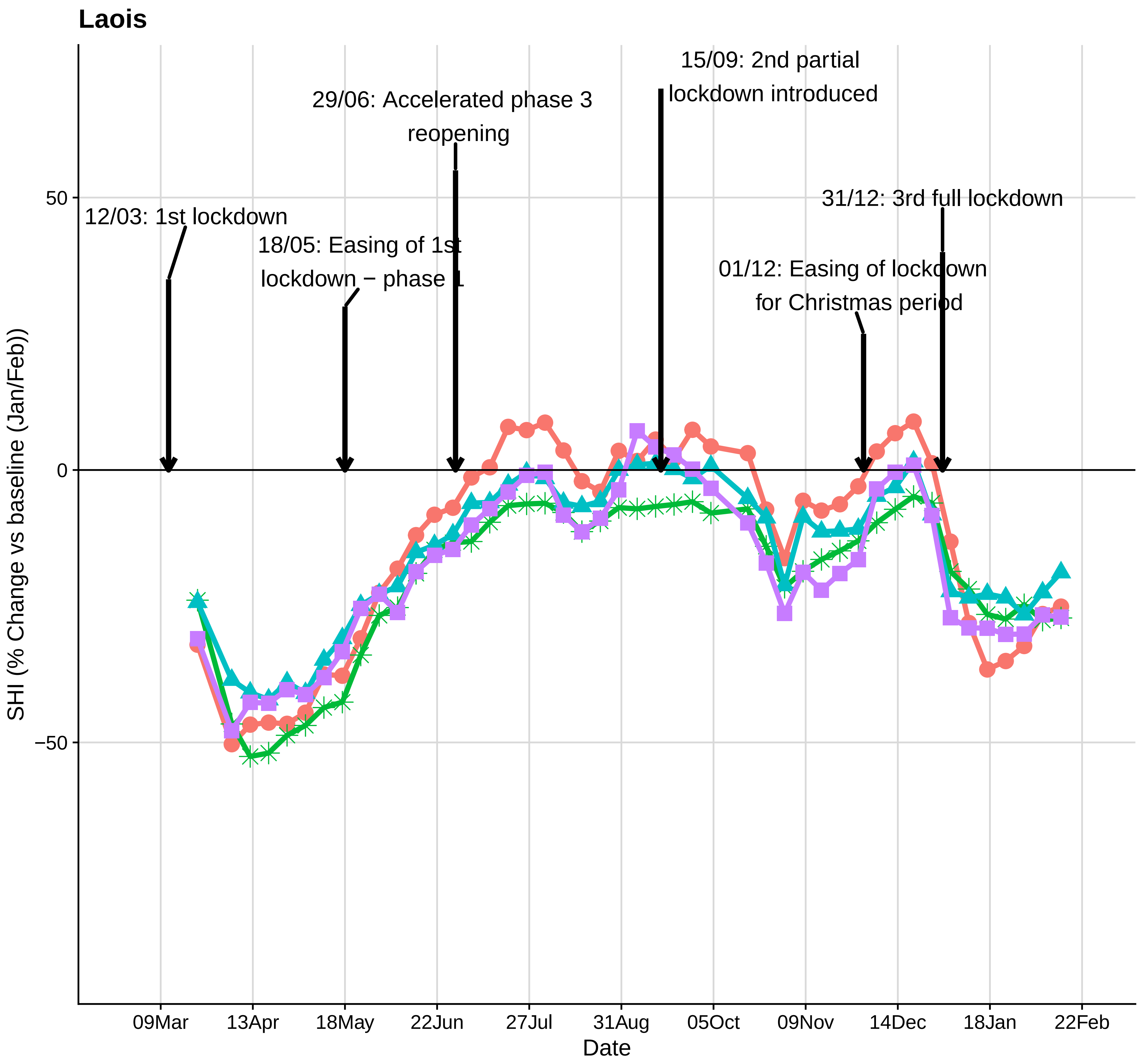

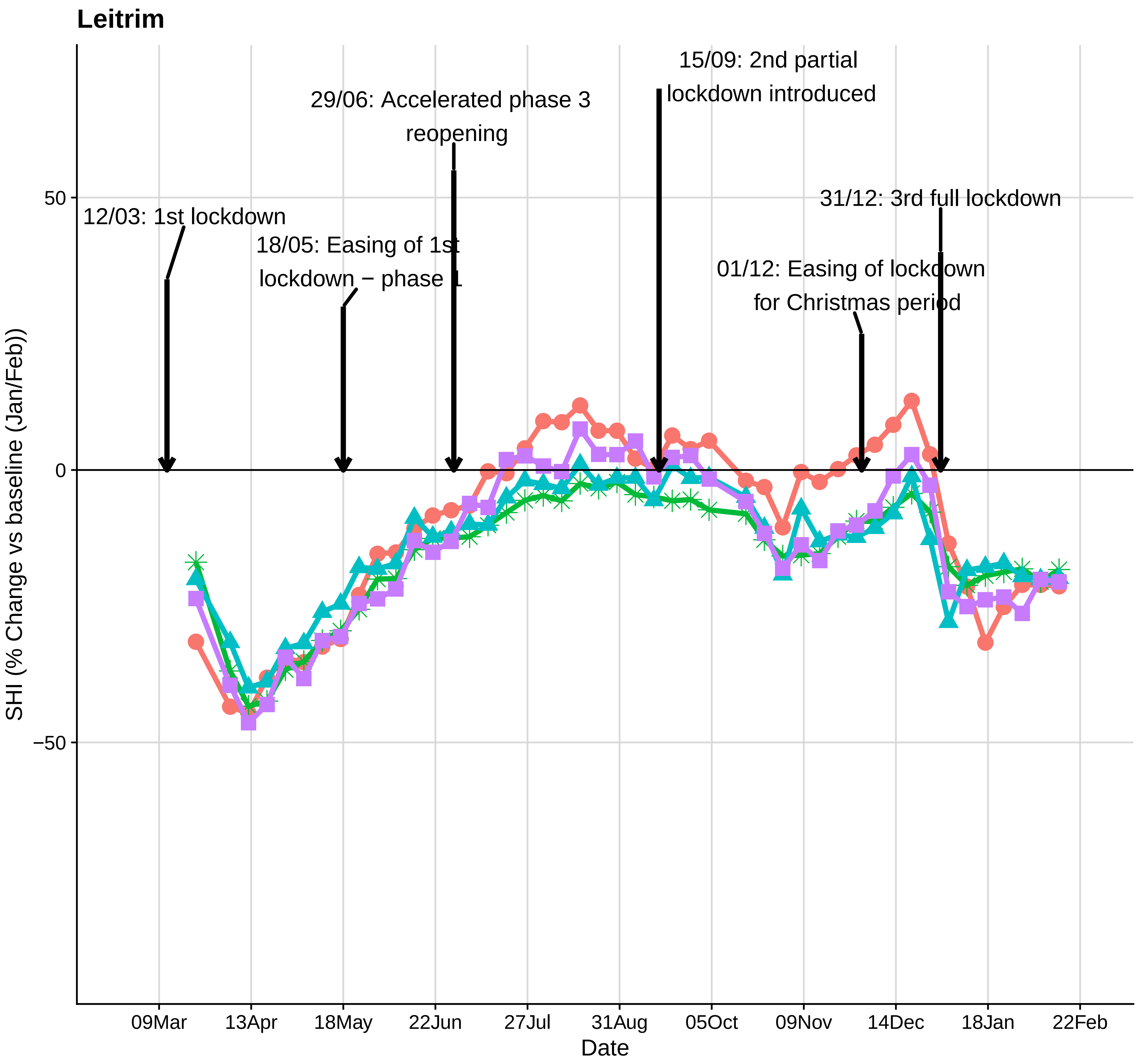

Age Bins u20 SHI 20−24 SHI 60−64 SHI o65 SHI

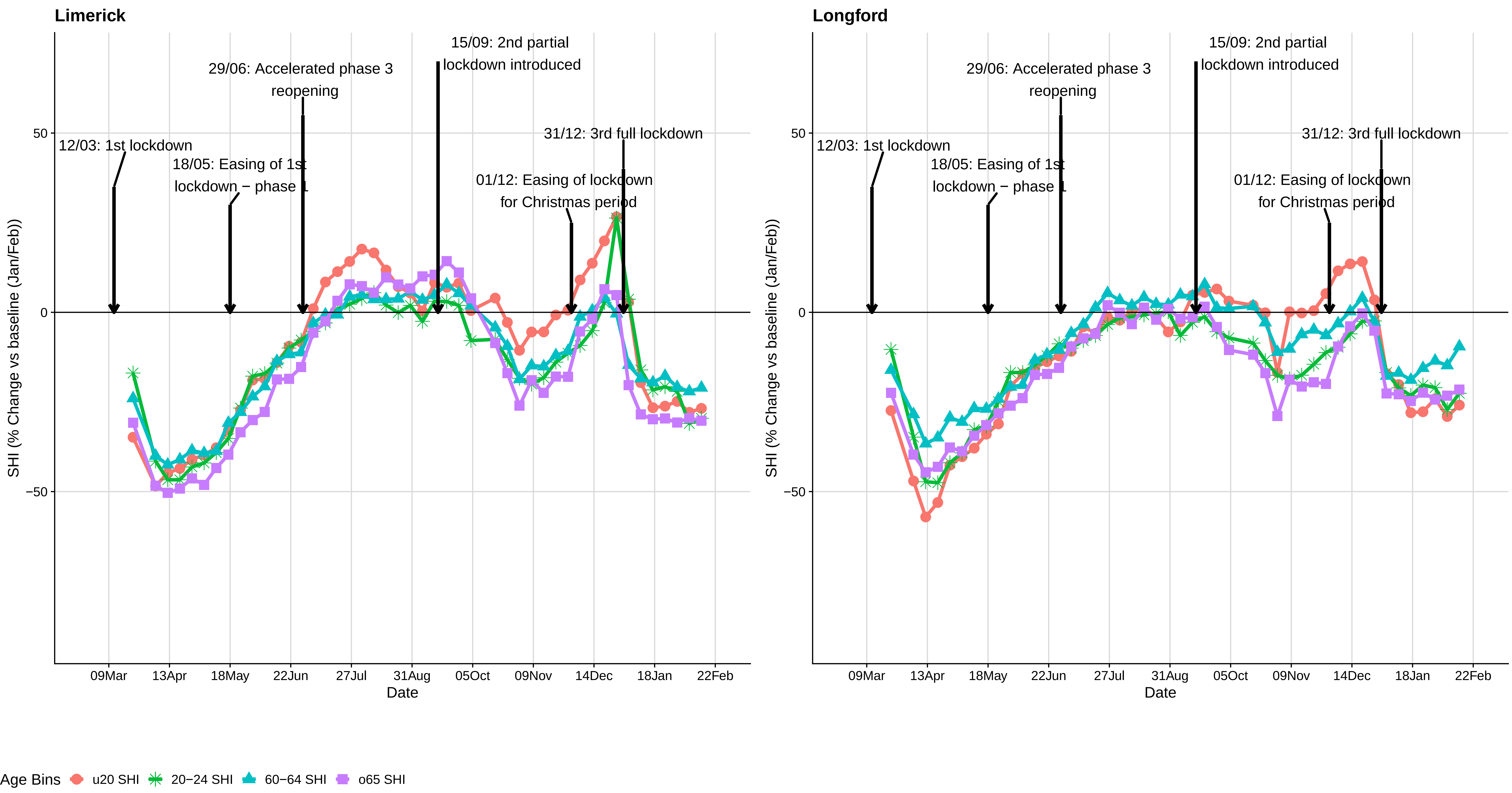
Limerick
Longford
15/09: 2nd partial lockdown introduced
29/06: Accelerated phase 3 reopening
31/12: 3rd full lockdown
12/03: 1st lockdown
18/05: Easing of 1st lockdown − phase 1
01/12: Easing of lockdown for Christmas period
SHI (% Change vs baseline (Jan/Feb))
50
0
−50
09Mar
13Apr
18May
22Jun
27Jul
31Aug
05Oct
09Nov
14Dec
18Jan
22Feb
Date
Age Bins
u20 SHI
20−24 SHI
60−64 SHI
o65 SHI

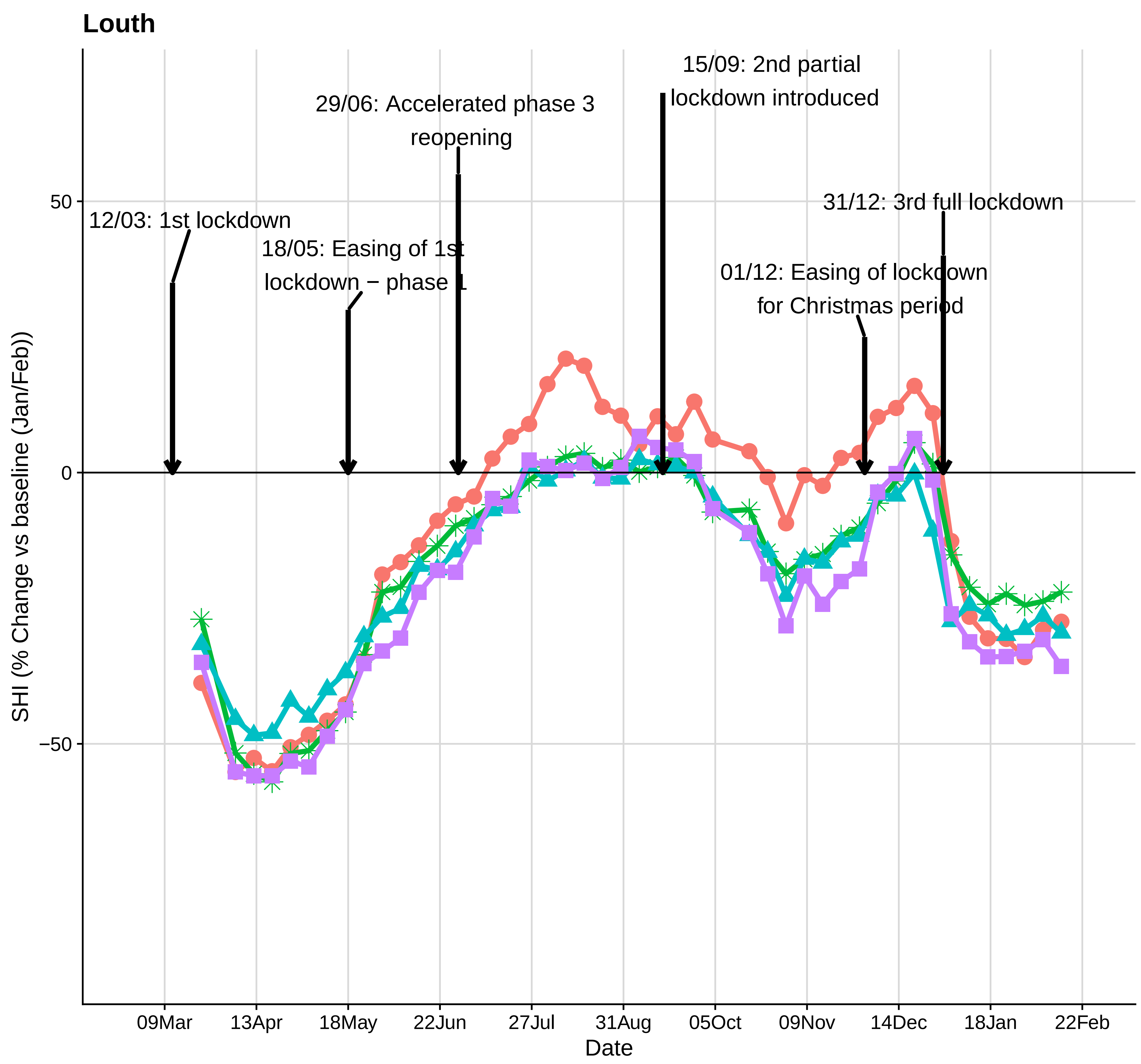

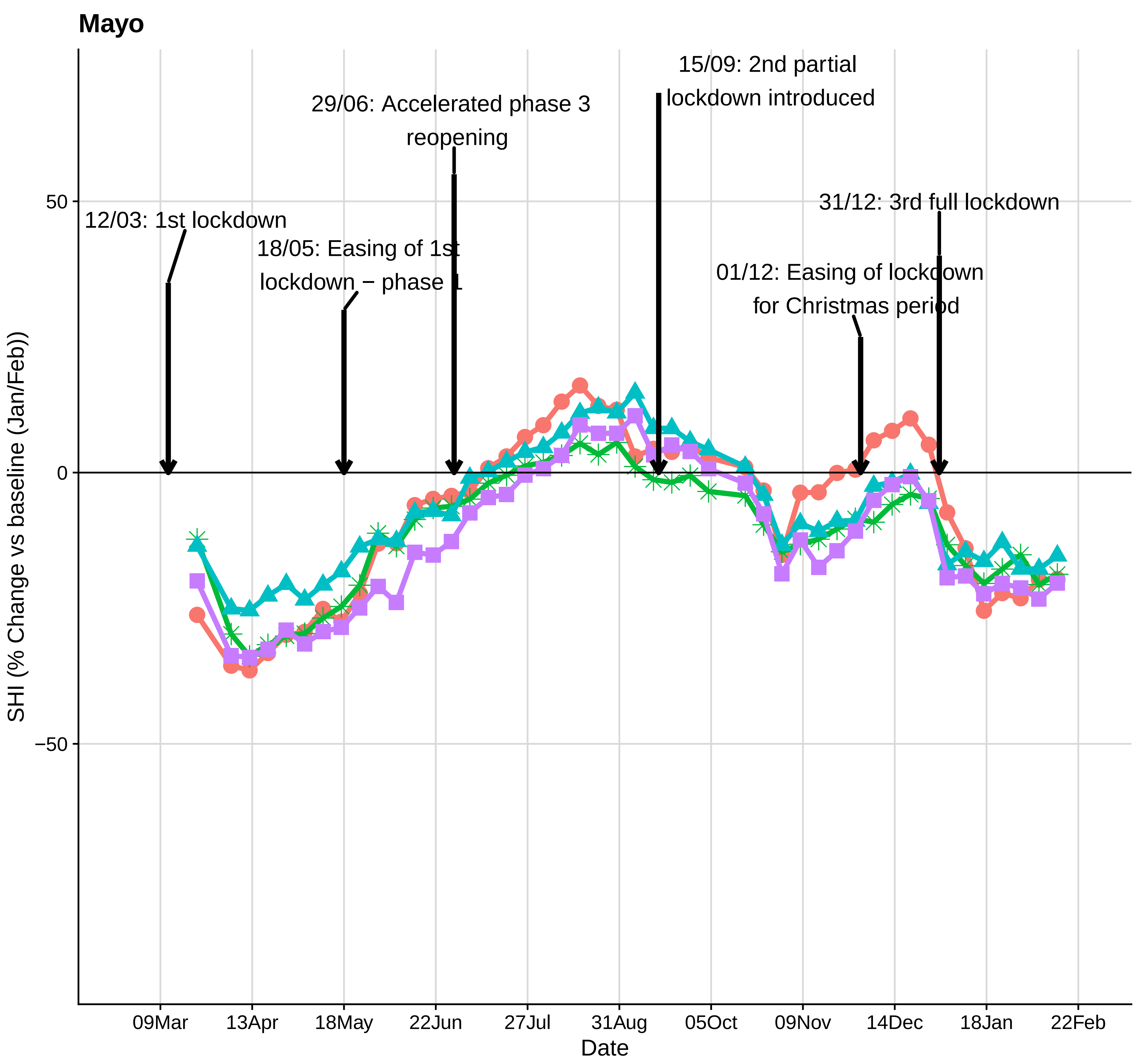

Age Bins u20 SHI 20−24 SHI 60−64 SHI o65 SHI

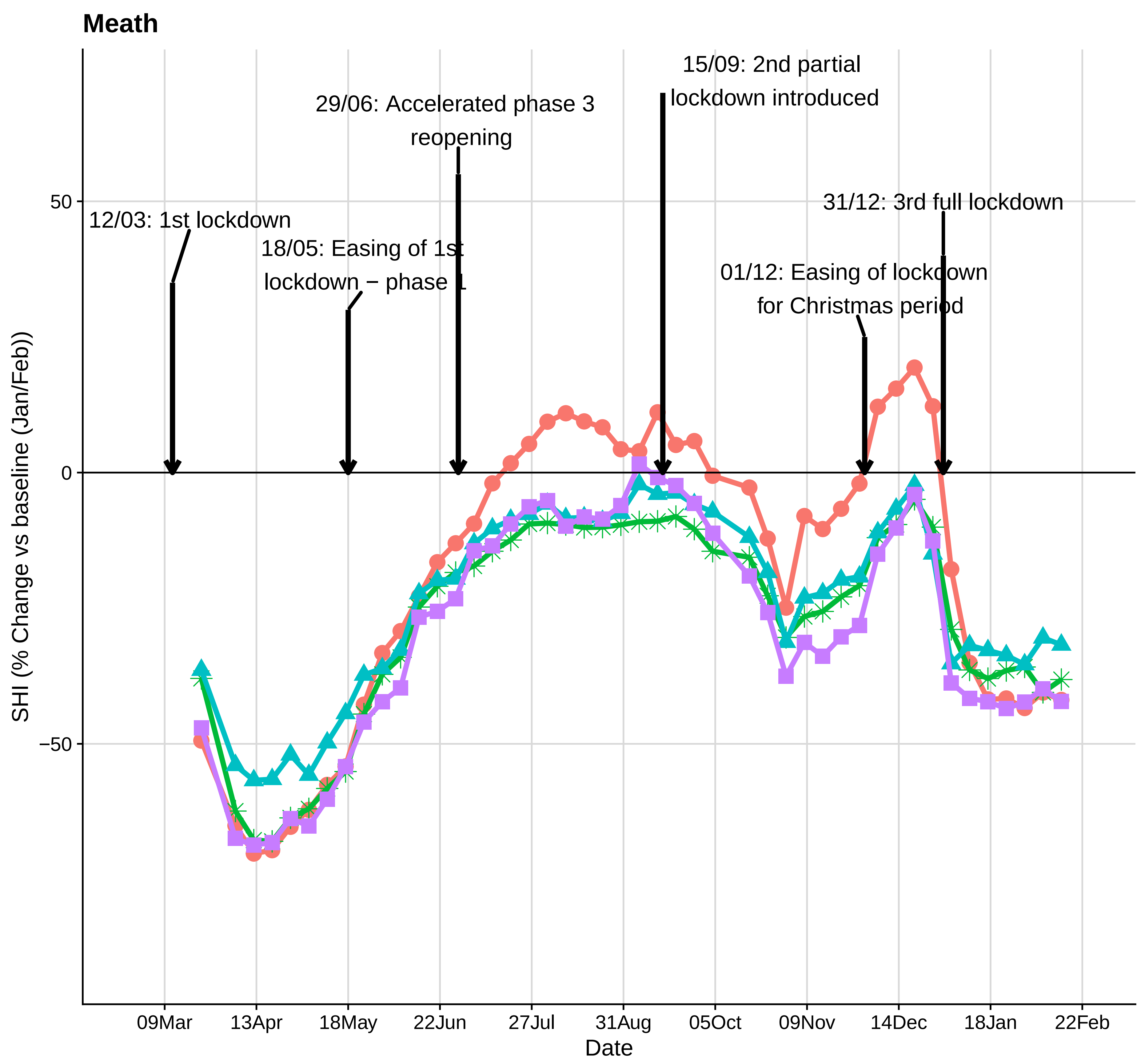

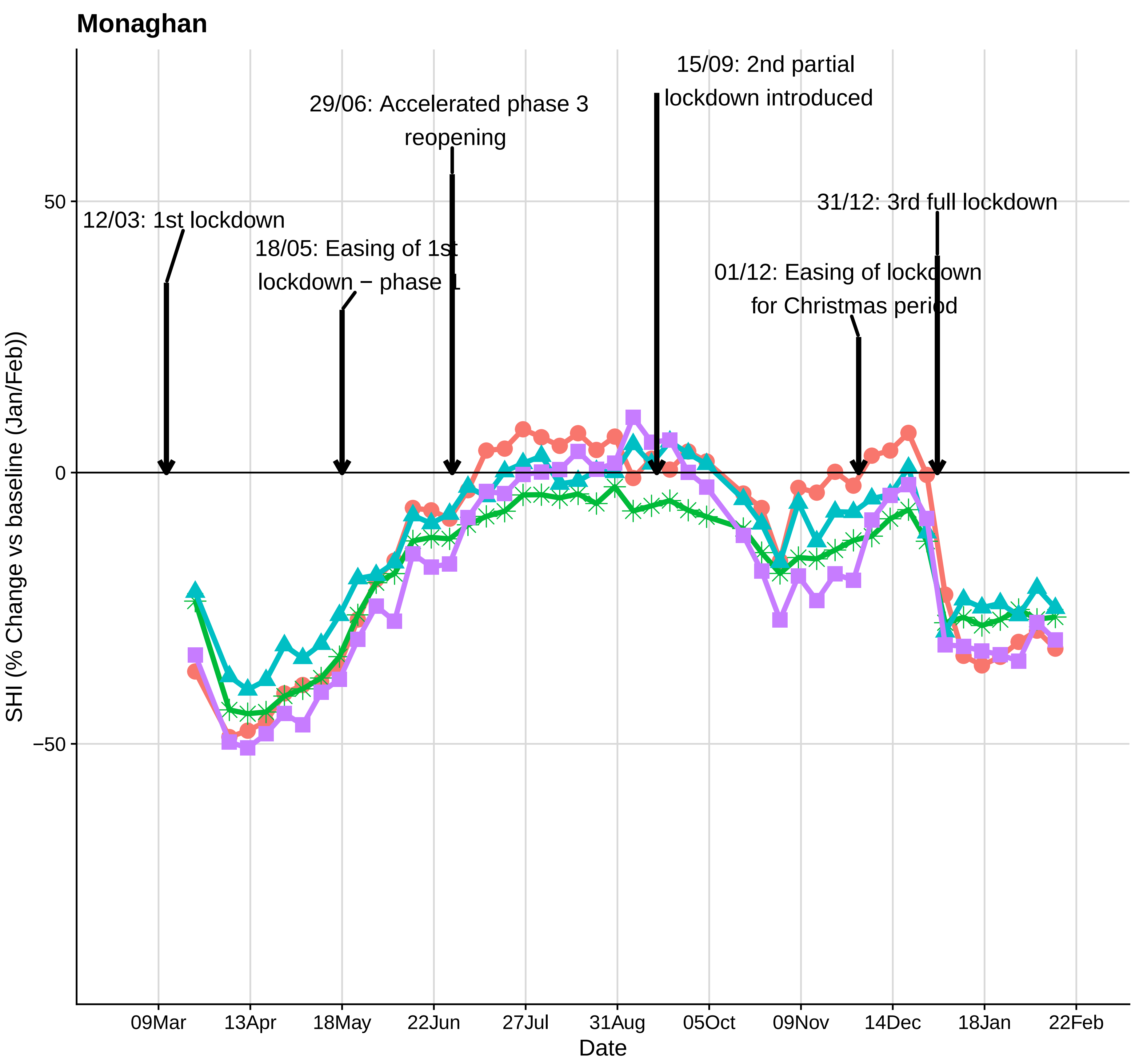

Age Bins u20 SHI 20−24 SHI 60−64 SHI o65 SHI

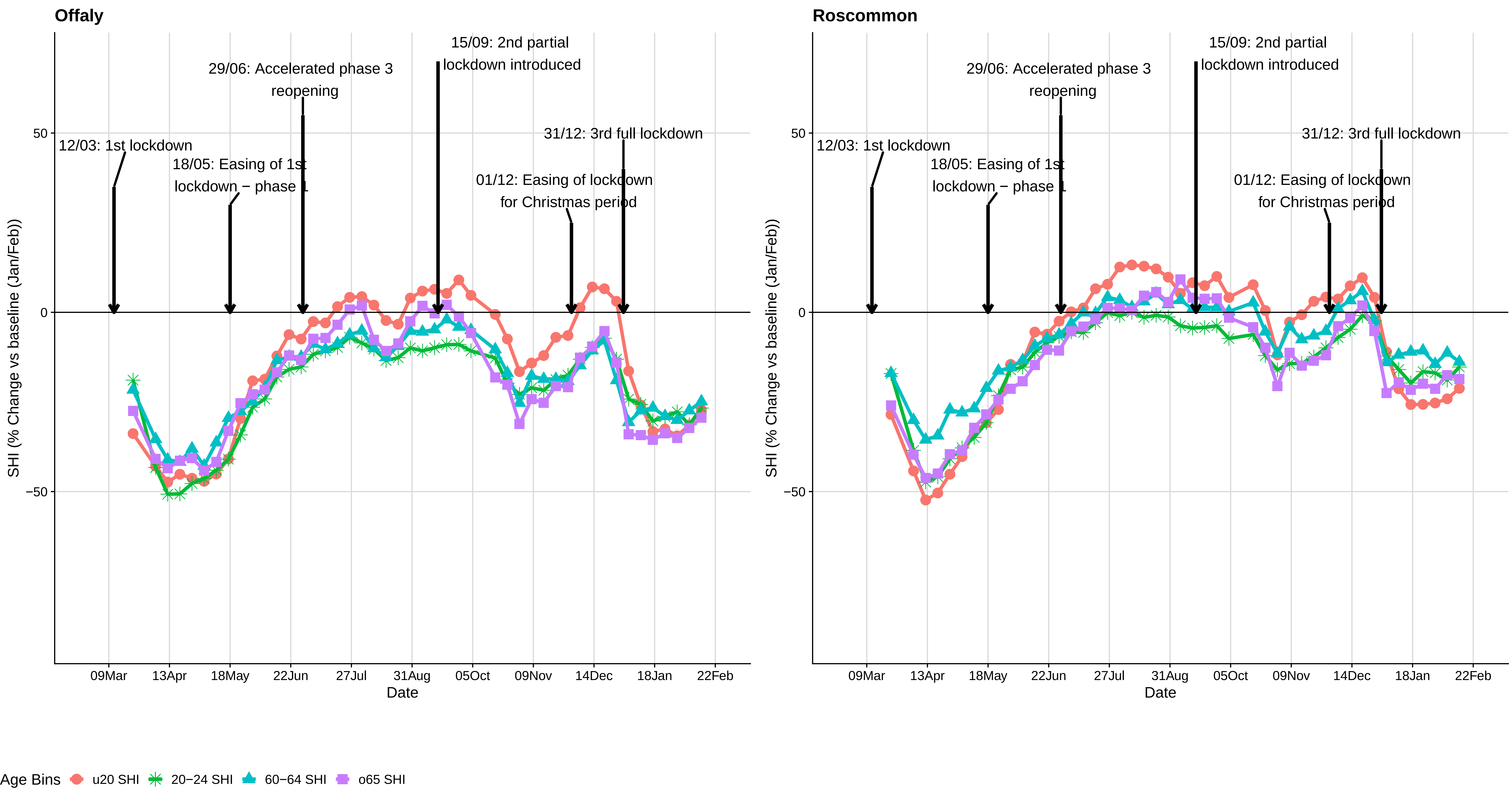
Offaly
Roscommon
12/03: 1st lockdown
18/05: Easing of 1st lockdown − phase 1
29/06: Accelerated phase 3 reopening
15/09: 2nd partial lockdown introduced
01/12: Easing of lockdown for Christmas period
31/12: 3rd full lockdown
SHI (% Change vs baseline (Jan/Feb))
50
0
−50
09Mar
13Apr
18May
22Jun
27Jul
31Aug
05Oct
09Nov
14Dec
18Jan
22Feb
Date
Age Bins
u20 SHI
20−24 SHI
60−64 SHI
o65 SHI

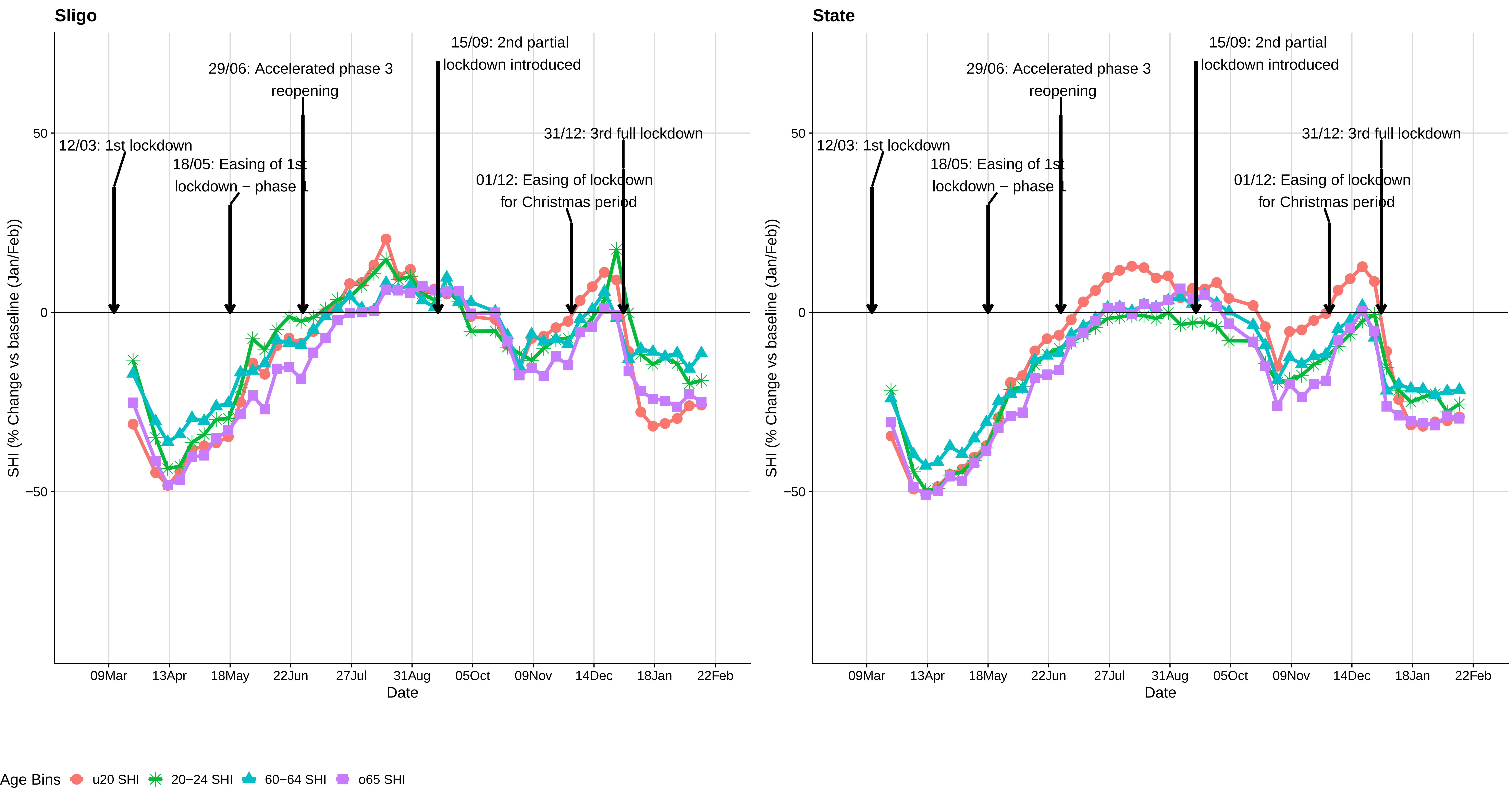
Sligo
State
12/03: 1st lockdown
18/05: Easing of 1st lockdown − phase 1
29/06: Accelerated phase 3 reopening
15/09: 2nd partial lockdown introduced
01/12: Easing of lockdown for Christmas period
31/12: 3rd full lockdown
SHI (% Change vs baseline (Jan/Feb))
50
0
−50
09Mar
13Apr
18May
22Jun
27Jul
31Aug
05Oct
09Nov
14Dec
18Jan
22Feb
Date
Age Bins
u20 SHI
20−24 SHI
60−64 SHI
o65 SHI

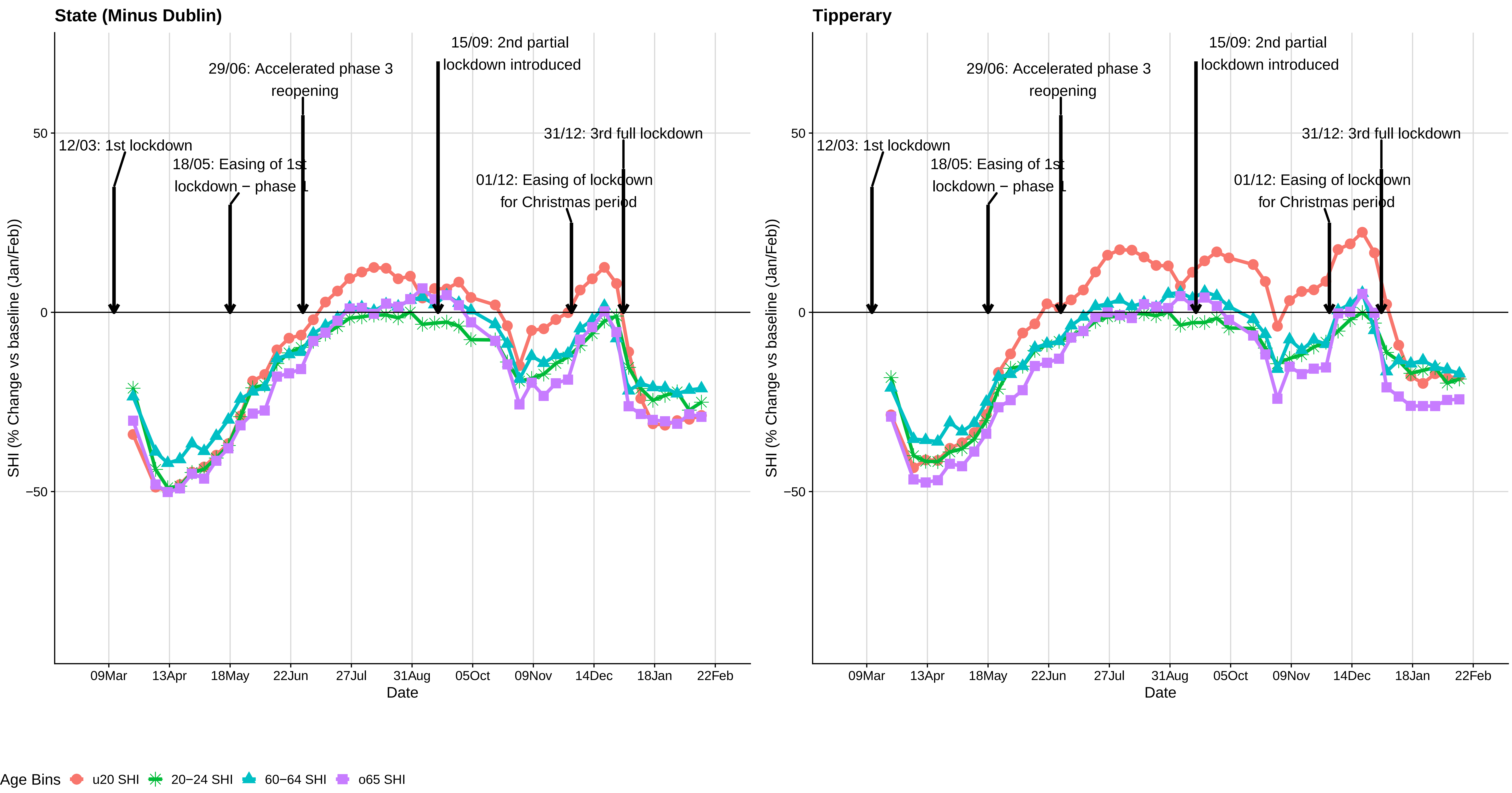
State (Minus Dublin)
Tipperary
15/09: 2nd partial lockdown introduced
29/06: Accelerated phase 3 reopening
31/12: 3rd full lockdown
12/03: 1st lockdown
18/05: Easing of 1st lockdown − phase 1
01/12: Easing of lockdown for Christmas period
SHI (% Change vs baseline (Jan/Feb))
50
0
−50
09Mar
13Apr
18May
22Jun
27Jul
31Aug
05Oct
09Nov
14Dec
18Jan
22Feb
Date
Age Bins
u20 SHI
20−24 SHI
60−64 SHI
o65 SHI

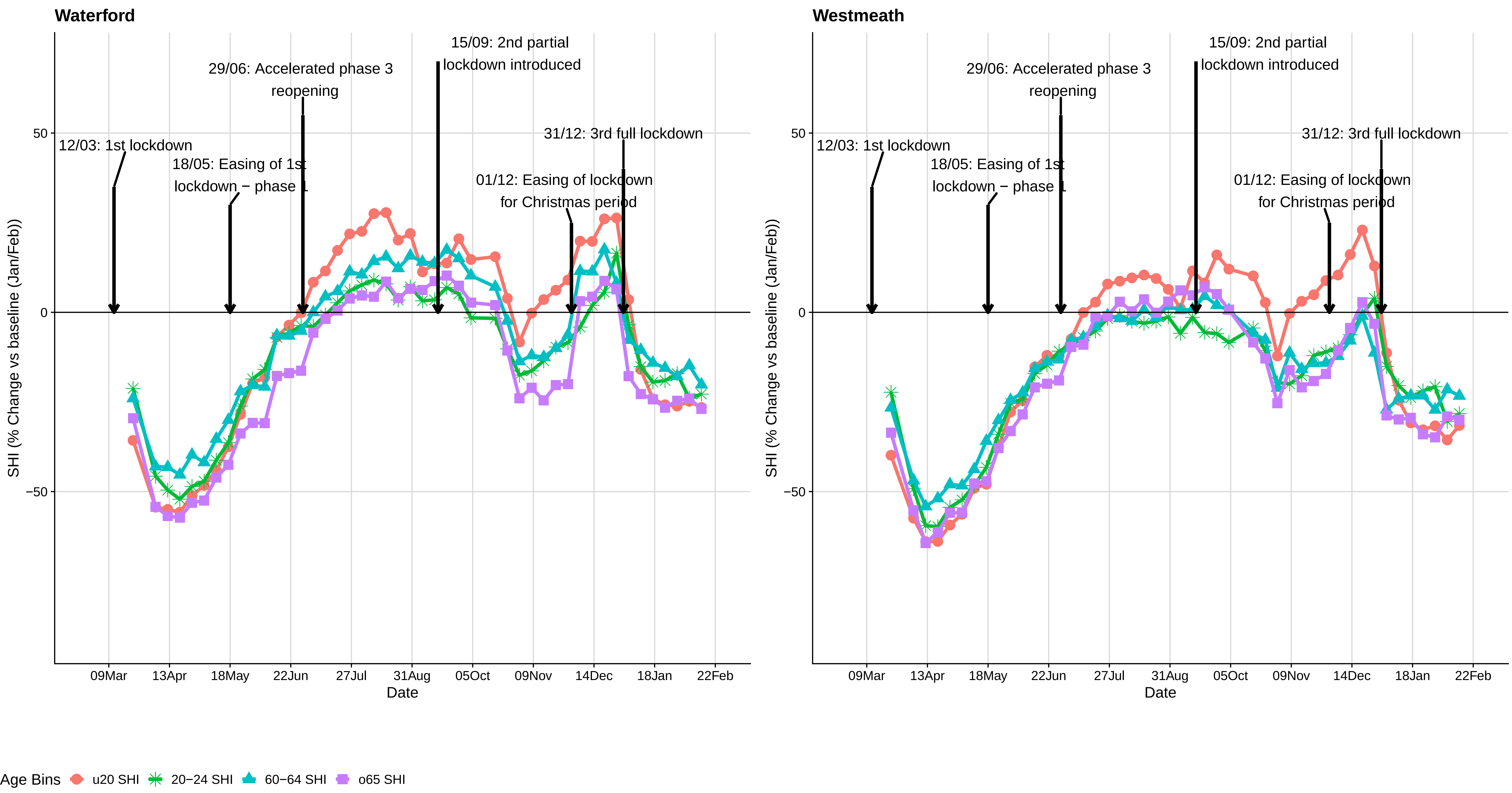
Waterford
Westmeath
15/09: 2nd partial
lockdown introduced
29/06: Accelerated phase 3
reopening
31/12: 3rd full lockdown
12/03: 1st lockdown
18/05: Easing of 1st
lockdown − phase 1
01/12: Easing of lockdown
for Christmas period
50
0
−50
SHI (% Change vs baseline (Jan/Feb))
09Mar
13Apr
18May
22Jun
27Jul
31Aug
05Oct
09Nov
14Dec
18Jan
22Feb
Date
Age Bins
u20 SHI
20−24 SHI
60−64 SHI
o65 SHI

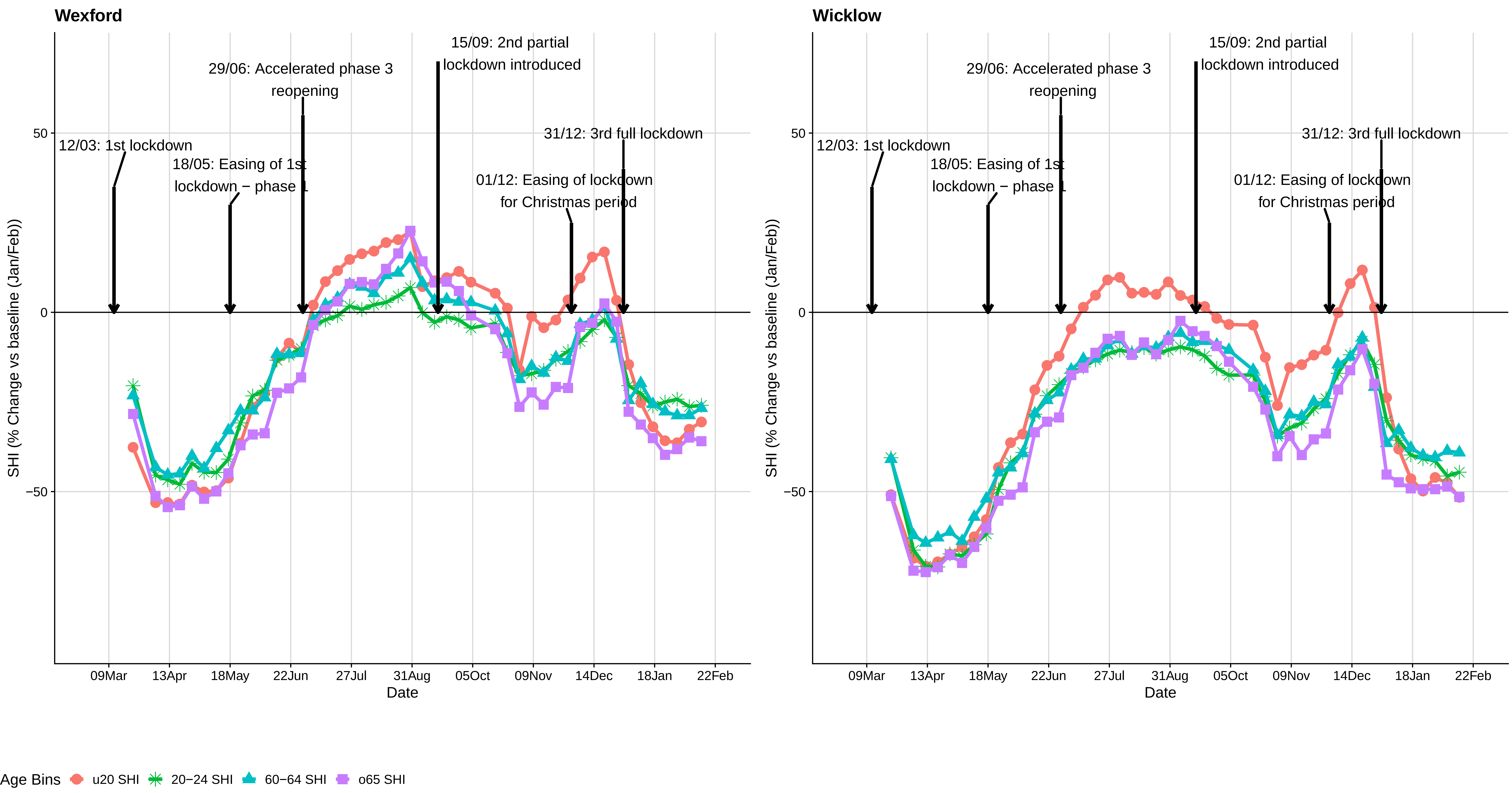
Wexford
Wicklow
12/03: 1st lockdown
18/05: Easing of 1st lockdown − phase 1
29/06: Accelerated phase 3 reopening
15/09: 2nd partial lockdown introduced
01/12: Easing of lockdown for Christmas period
31/12: 3rd full lockdown
SHI (% Change vs baseline (Jan/Feb))
50
0
−50
09Mar
13Apr
18May
22Jun
27Jul
31Aug
05Oct
09Nov
14Dec
18Jan
22Feb
Date
Age Bins
u20 SHI
20−24 SHI
60−64 SHI
o65 SHI
