# Supplementary material for: Population Mobility Trends, Deprivation Index and the Spatio-Temporal Spread of Coronavirus Disease 2019 in Ireland
Source: Int J Environ Res Public Health. 2021 Jun 10;18(12):6285. doi: 10.3390/ijerph18126285 (PMC8296107; doi:10.3390/ijerph18126285)
Supplement: Supplementary file 1 [file ijerph-18-06285-s001.zip › SM3_county_CM_age_all_counties08_Mar_2021.pdf]

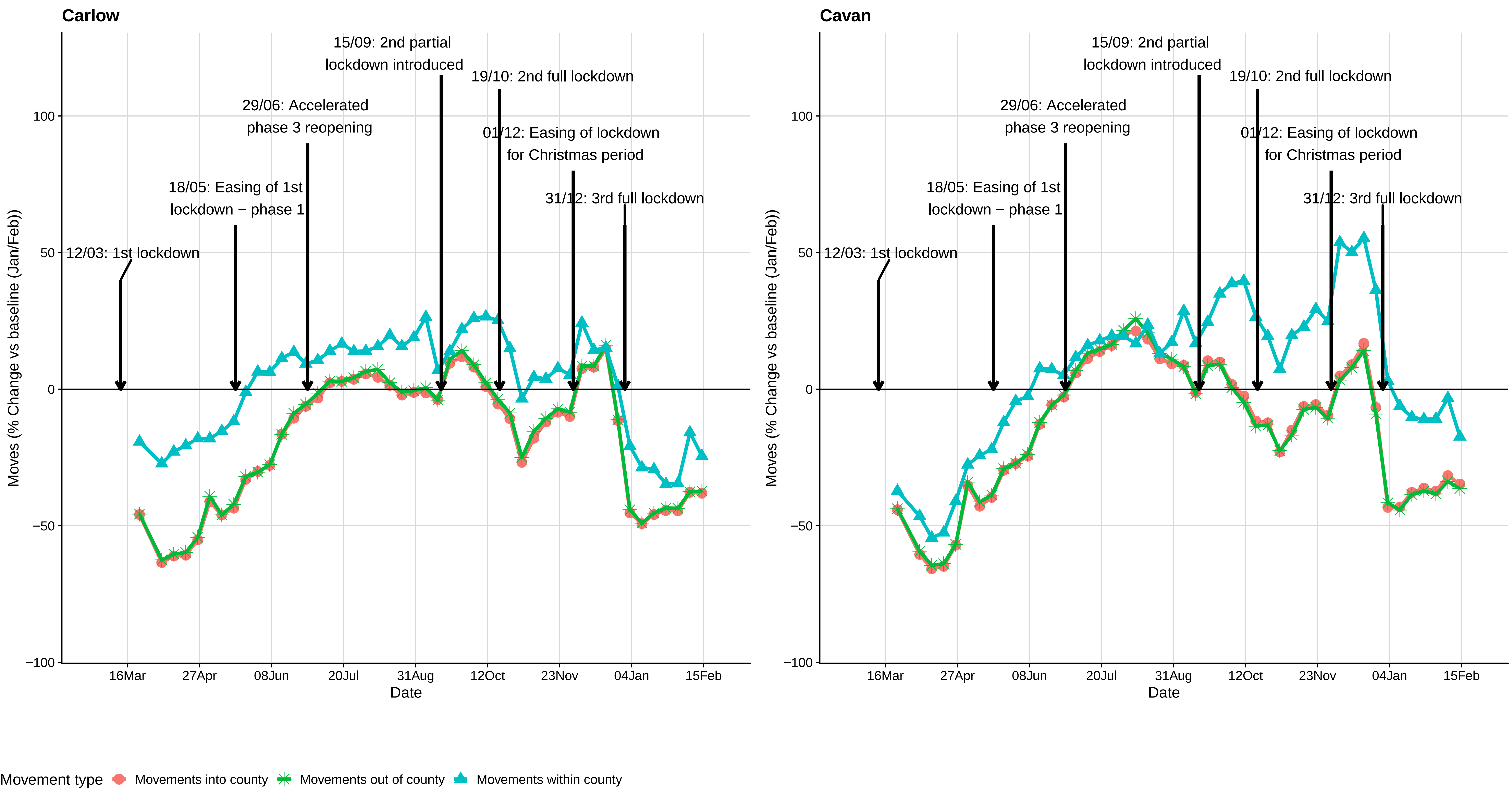
Carlow
Cavan
15/09: 2nd partial lockdown introduced
19/10: 2nd full lockdown
29/06: Accelerated phase 3 reopening
01/12: Easing of lockdown for Christmas period
18/05: Easing of 1st lockdown − phase 1
31/12: 3rd full lockdown
12/03: 1st lockdown
Moves (% Change vs baseline (Jan/Feb))
100
50
0
−50
−100
16Mar
27Apr
08Jun
20Jul
31Aug
12Oct
23Nov
04Jan
15Feb
Date
Movement type
Movements into county
Movements out of county
Movements within county

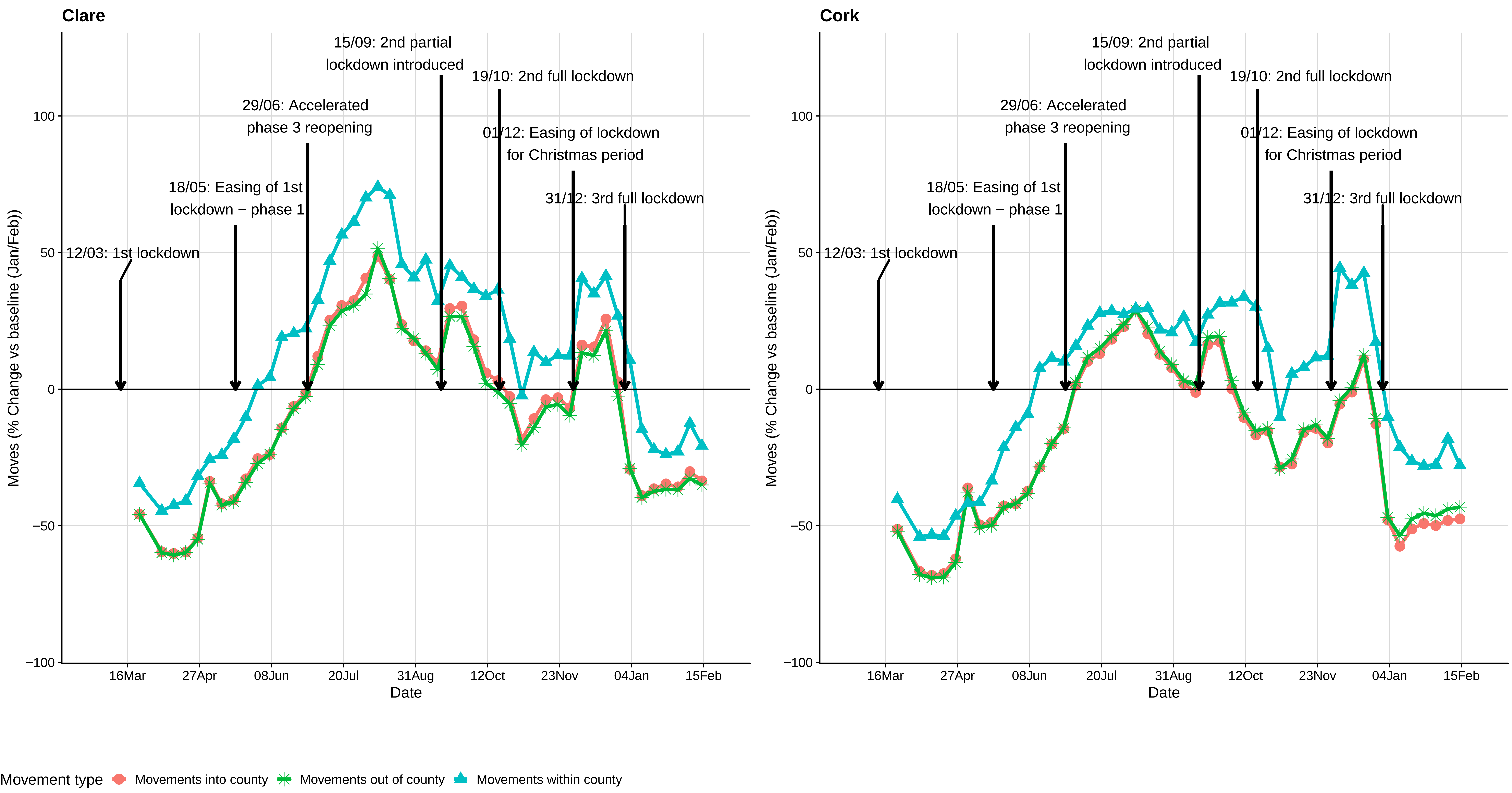
Clare
Cork
15/09: 2nd partial lockdown introduced
19/10: 2nd full lockdown
29/06: Accelerated phase 3 reopening
01/12: Easing of lockdown for Christmas period
18/05: Easing of 1st lockdown − phase 1
31/12: 3rd full lockdown
12/03: 1st lockdown
Moves (% Change vs baseline (Jan/Feb))
100
50
0
−50
−100
16Mar
27Apr
08Jun
20Jul
31Aug
12Oct
23Nov
04Jan
15Feb
Date
Movement type
Movements into county
Movements out of county
Movements within county

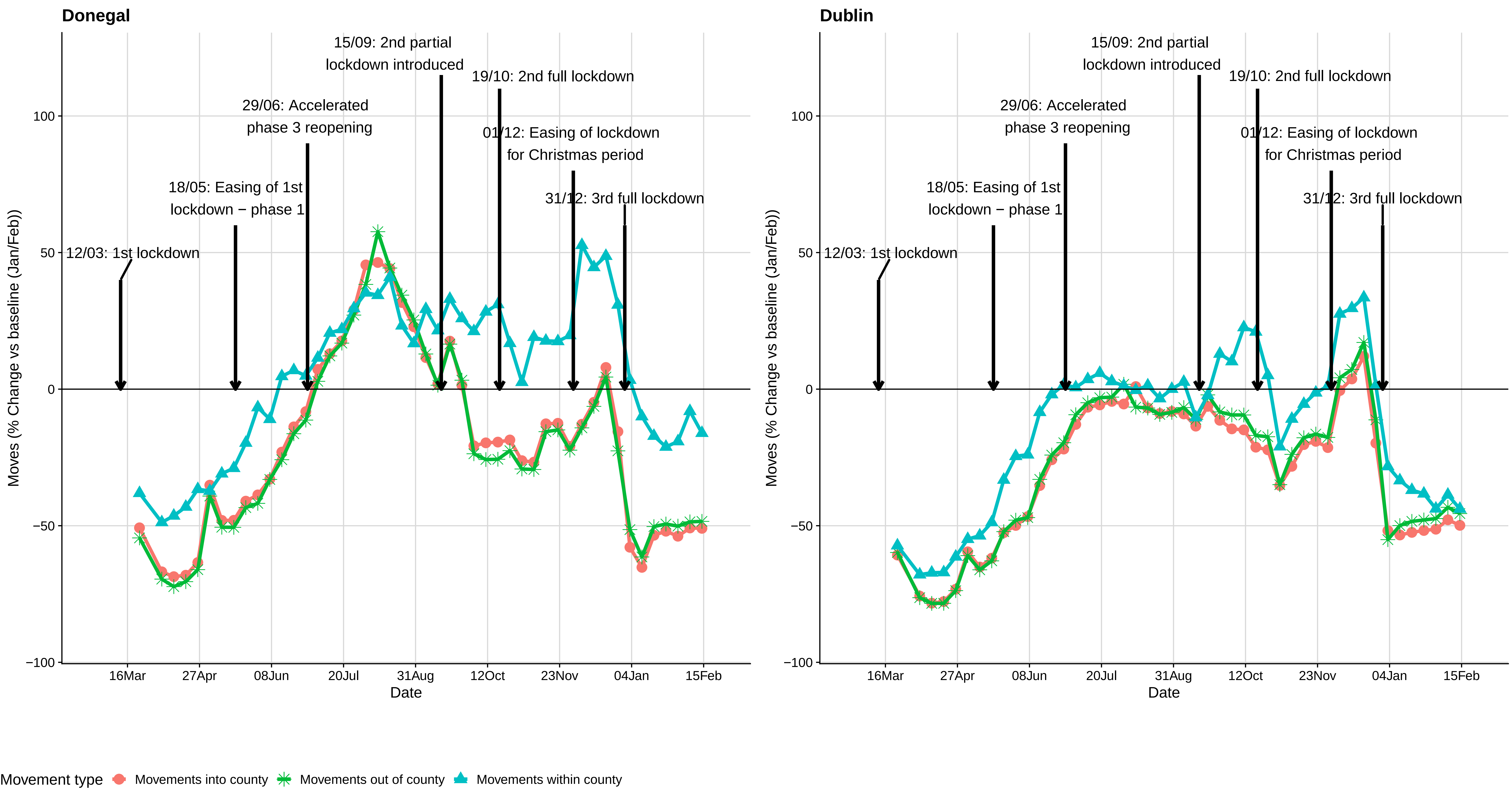

Donegal
Dublin
15/09: 2nd partial lockdown introduced
19/10: 2nd full lockdown
29/06: Accelerated phase 3 reopening
01/12: Easing of lockdown for Christmas period
18/05: Easing of 1st lockdown − phase 1
31/12: 3rd full lockdown
12/03: 1st lockdown
Moves (% Change vs baseline (Jan/Feb))
100
50
0
−50
−100
16Mar
27Apr
08Jun
20Jul
31Aug
12Oct
23Nov
04Jan
15Feb
Date
Movement type
Movements into county
Movements out of county
Movements within county

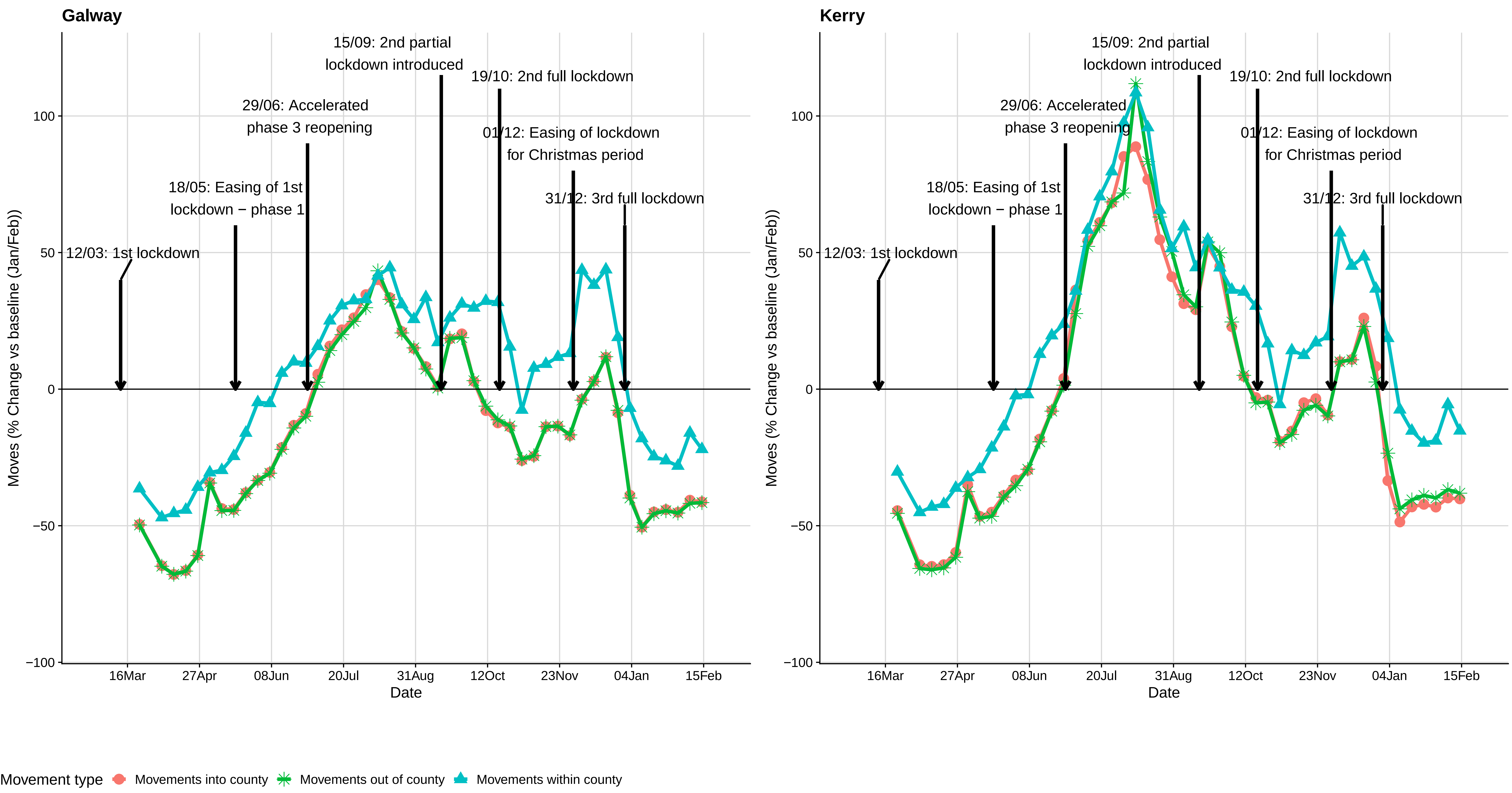
Galway
Kerry
15/09: 2nd partial lockdown introduced
19/10: 2nd full lockdown
29/06: Accelerated phase 3 reopening
01/12: Easing of lockdown for Christmas period
18/05: Easing of 1st lockdown − phase 1
31/12: 3rd full lockdown
12/03: 1st lockdown
Moves (% Change vs baseline (Jan/Feb))
100
50
0
−50
−100
16Mar
27Apr
08Jun
20Jul
31Aug
12Oct
23Nov
04Jan
15Feb
Date
Movement type
Movements into county
Movements out of county
Movements within county

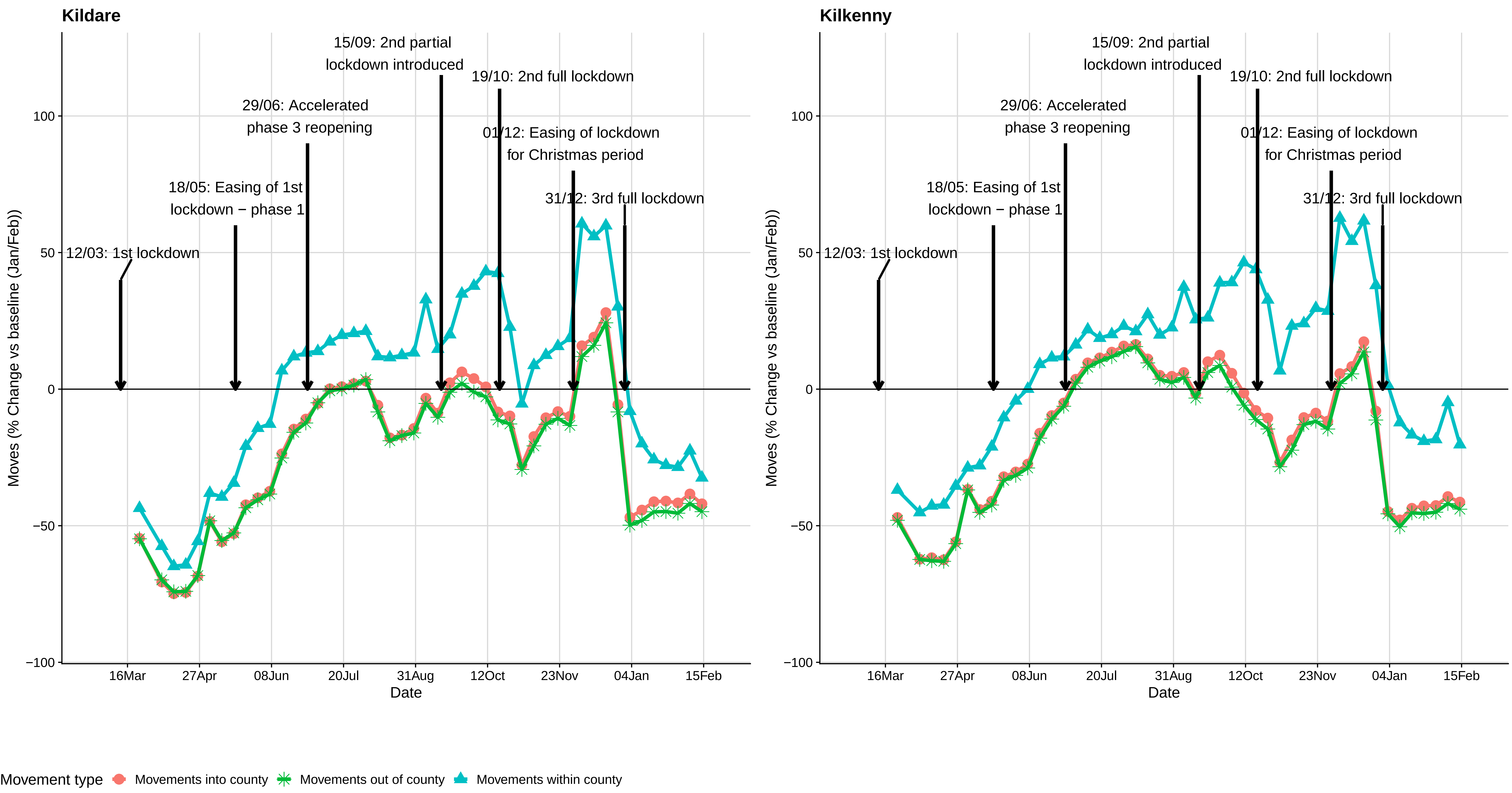
Kildare
15/09: 2nd partial lockdown introduced
19/10: 2nd full lockdown
29/06: Accelerated phase 3 reopening
01/12: Easing of lockdown for Christmas period
18/05: Easing of 1st lockdown − phase 1
31/12: 3rd full lockdown
12/03: 1st lockdown
100
50
0
−50
−100
Moves (% Change vs baseline (Jan/Feb))
16Mar
27Apr
08Jun
20Jul
31Aug
12Oct
23Nov
04Jan
15Feb
Date
Kilkenny
15/09: 2nd partial lockdown introduced
19/10: 2nd full lockdown
29/06: Accelerated phase 3 reopening
01/12: Easing of lockdown for Christmas period
18/05: Easing of 1st lockdown − phase 1
31/12: 3rd full lockdown
12/03: 1st lockdown
Movement type
Movements into county
Movements out of county
Movements within county

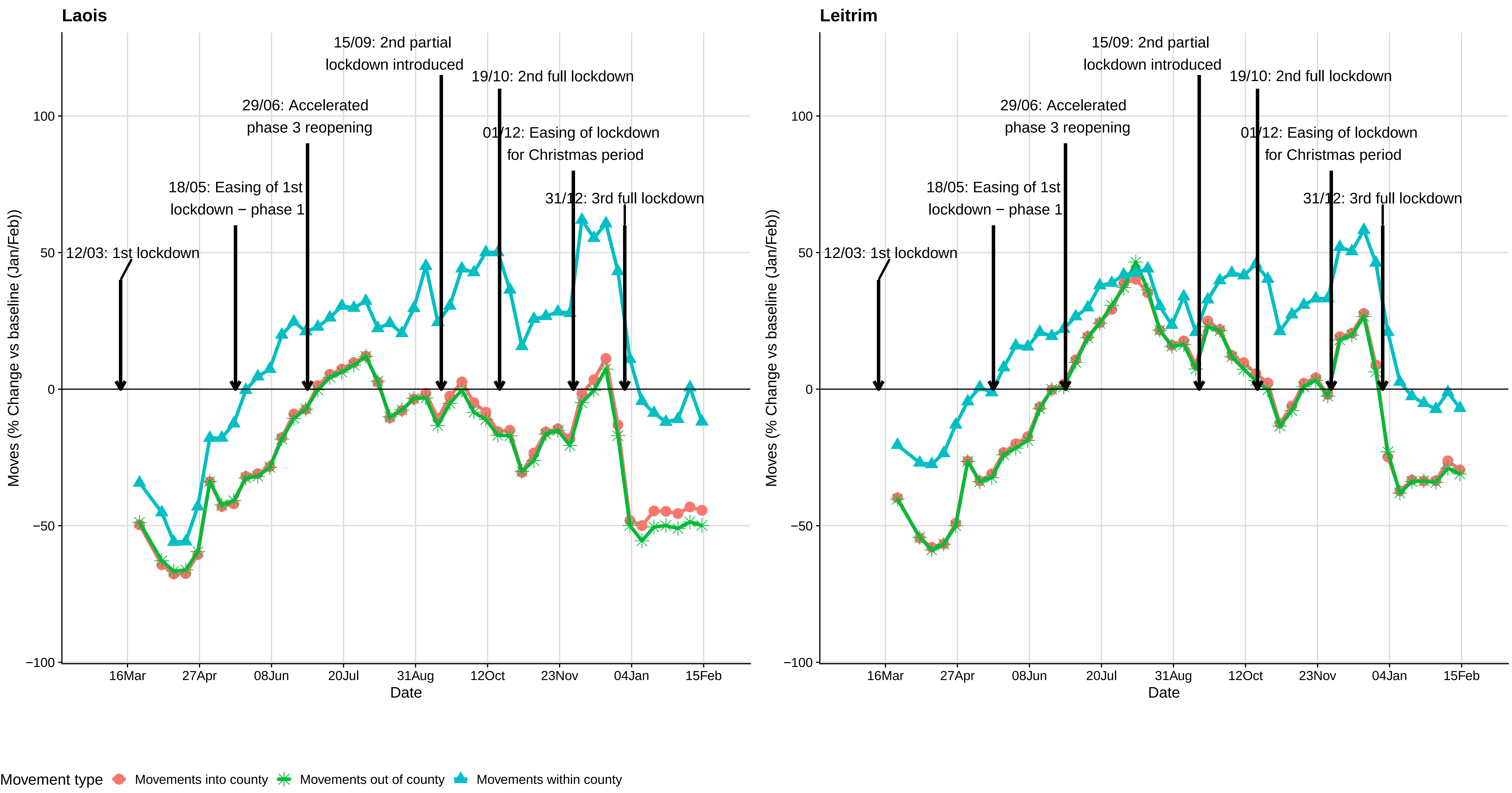
Laois
Leitrim
15/09: 2nd partial lockdown introduced
19/10: 2nd full lockdown
29/06: Accelerated phase 3 reopening
01/12: Easing of lockdown for Christmas period
18/05: Easing of 1st lockdown − phase 1
31/12: 3rd full lockdown
12/03: 1st lockdown
Moves (% Change vs baseline (Jan/Feb))
100
50
0
−50
−100
16Mar
27Apr
08Jun
20Jul
31Aug
12Oct
23Nov
04Jan
15Feb
Date
Movement type
Movements into county
Movements out of county
Movements within county

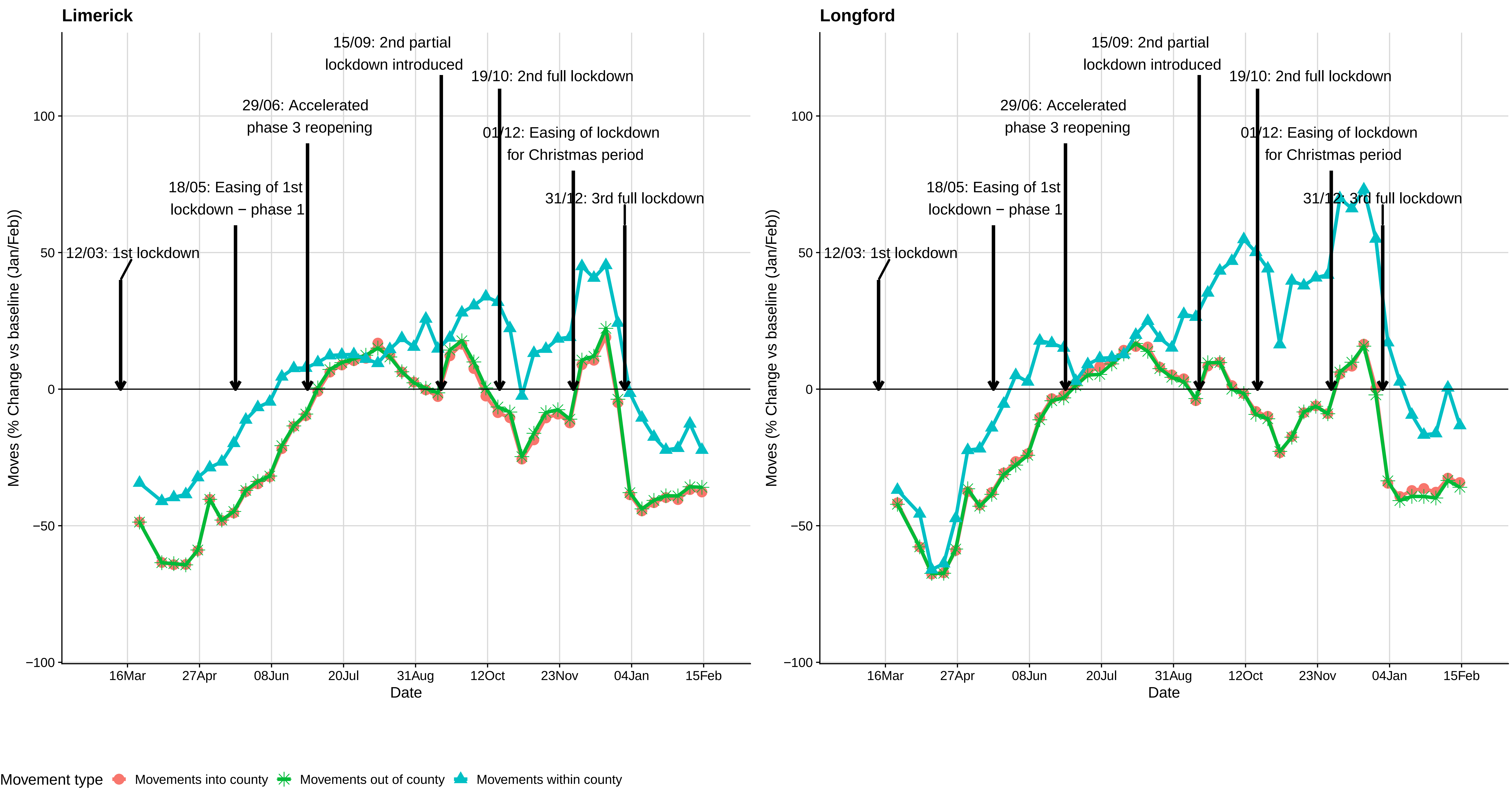

Limerick
Longford
15/09: 2nd partial lockdown introduced
19/10: 2nd full lockdown
29/06: Accelerated phase 3 reopening
01/12: Easing of lockdown for Christmas period
18/05: Easing of 1st lockdown − phase 1
31/12: 3rd full lockdown
12/03: 1st lockdown
Moves (% Change vs baseline (Jan/Feb))
100
50
0
−50
−100
16Mar
27Apr
08Jun
20Jul
31Aug
12Oct
23Nov
04Jan
15Feb
Date
Movement type
Movements into county
Movements out of county
Movements within county

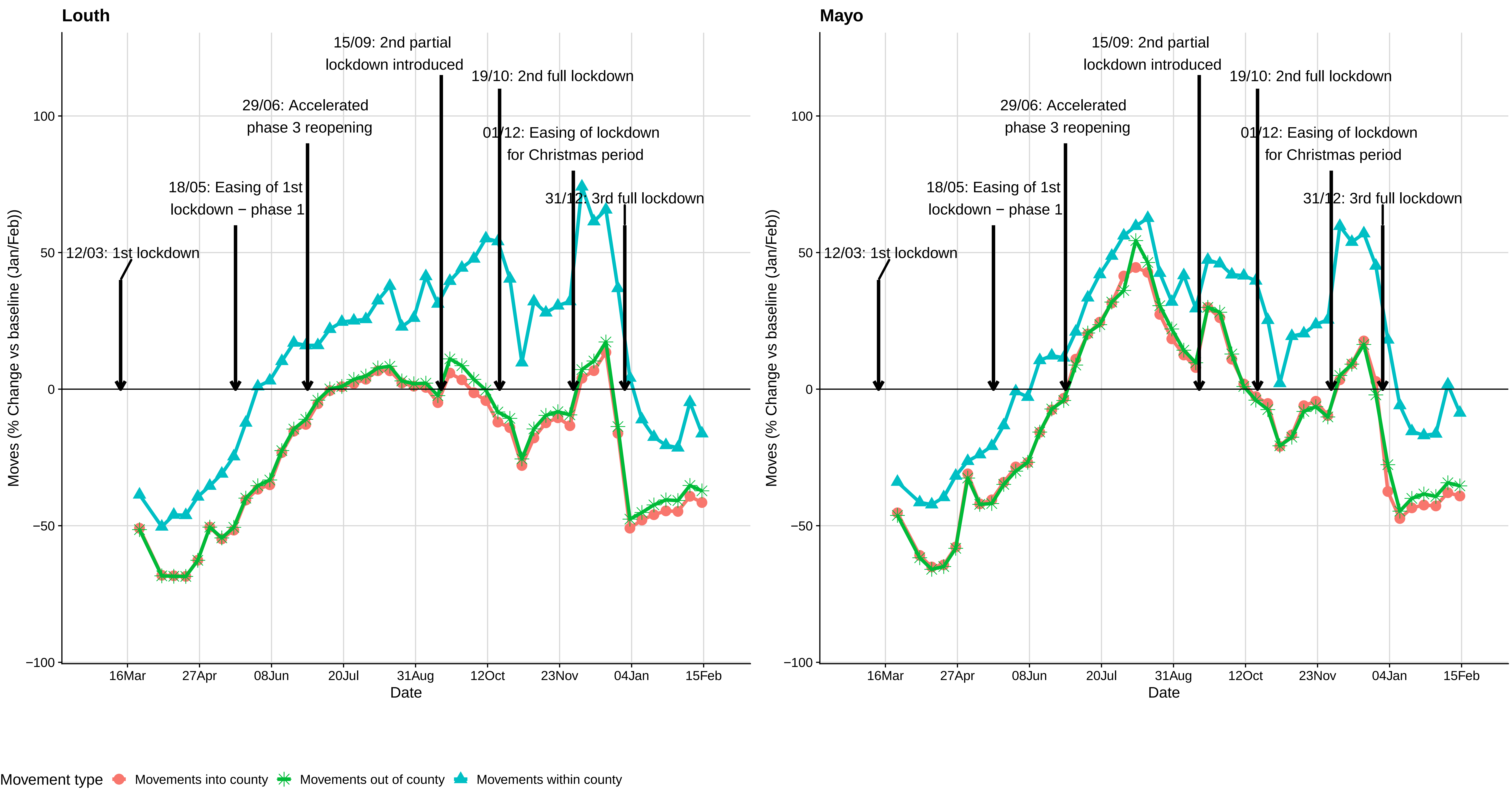

Louth
Mayo
12/03: 1st lockdown
18/05: Easing of 1st lockdown − phase 1
29/06: Accelerated phase 3 reopening
15/09: 2nd partial lockdown introduced
19/10: 2nd full lockdown
01/12: Easing of lockdown for Christmas period
31/12: 3rd full lockdown
Moves (% Change vs baseline (Jan/Feb))
100
50
0
−50
−100
16Mar
27Apr
08Jun
20Jul
31Aug
12Oct
23Nov
04Jan
15Feb
Date
Movement type
Movements into county
Movements out of county
Movements within county

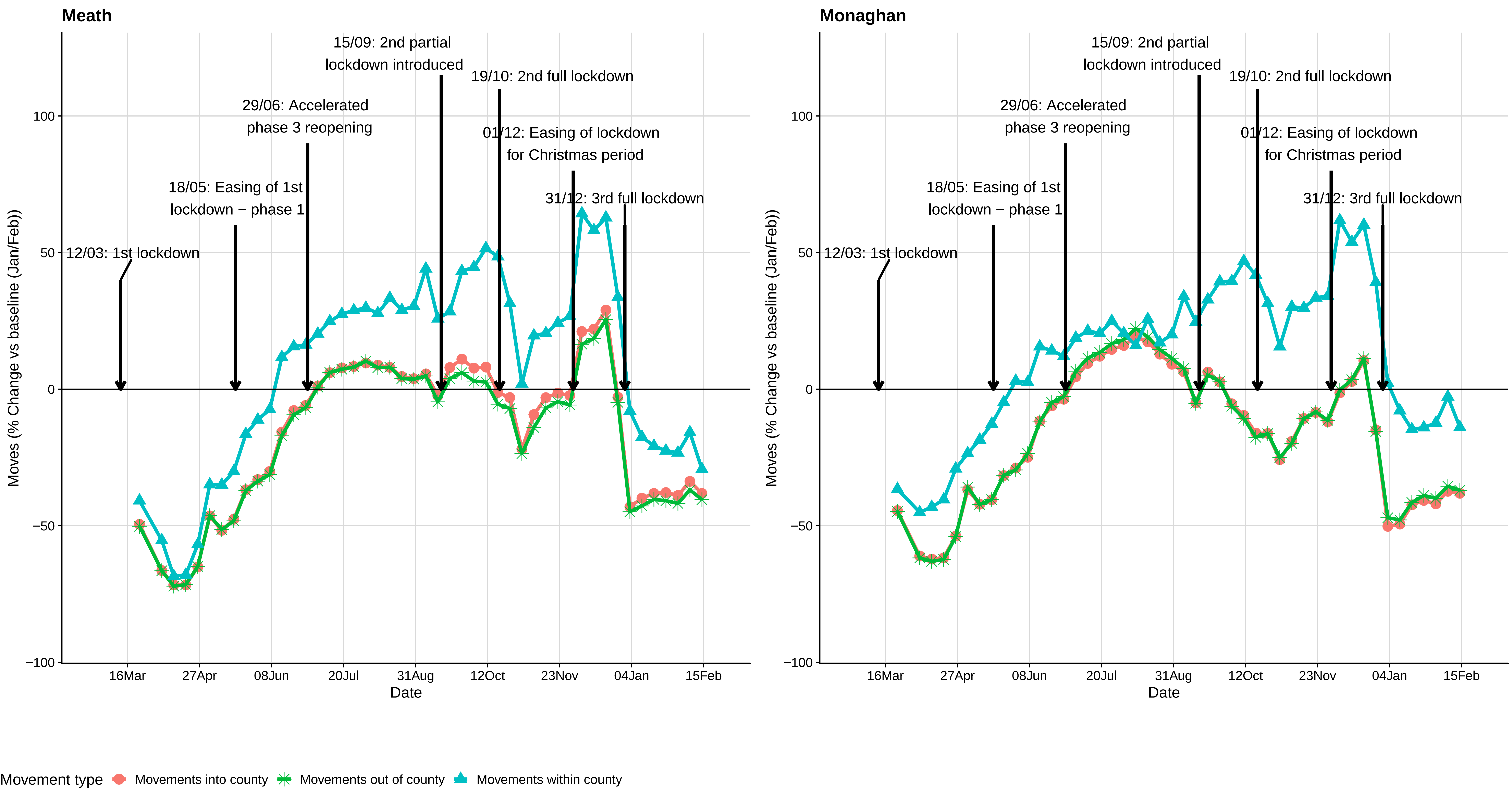
Meath
Monaghan
12/03: 1st lockdown
18/05: Easing of 1st lockdown − phase 1
29/06: Accelerated phase 3 reopening
15/09: 2nd partial lockdown introduced
19/10: 2nd full lockdown
01/12: Easing of lockdown for Christmas period
31/12: 3rd full lockdown
Moves (% Change vs baseline (Jan/Feb))
100
50
0
−50
−100
16Mar
27Apr
08Jun
20Jul
31Aug
12Oct
23Nov
04Jan
15Feb
Date
Movement type
Movements into county
Movements out of county
Movements within county

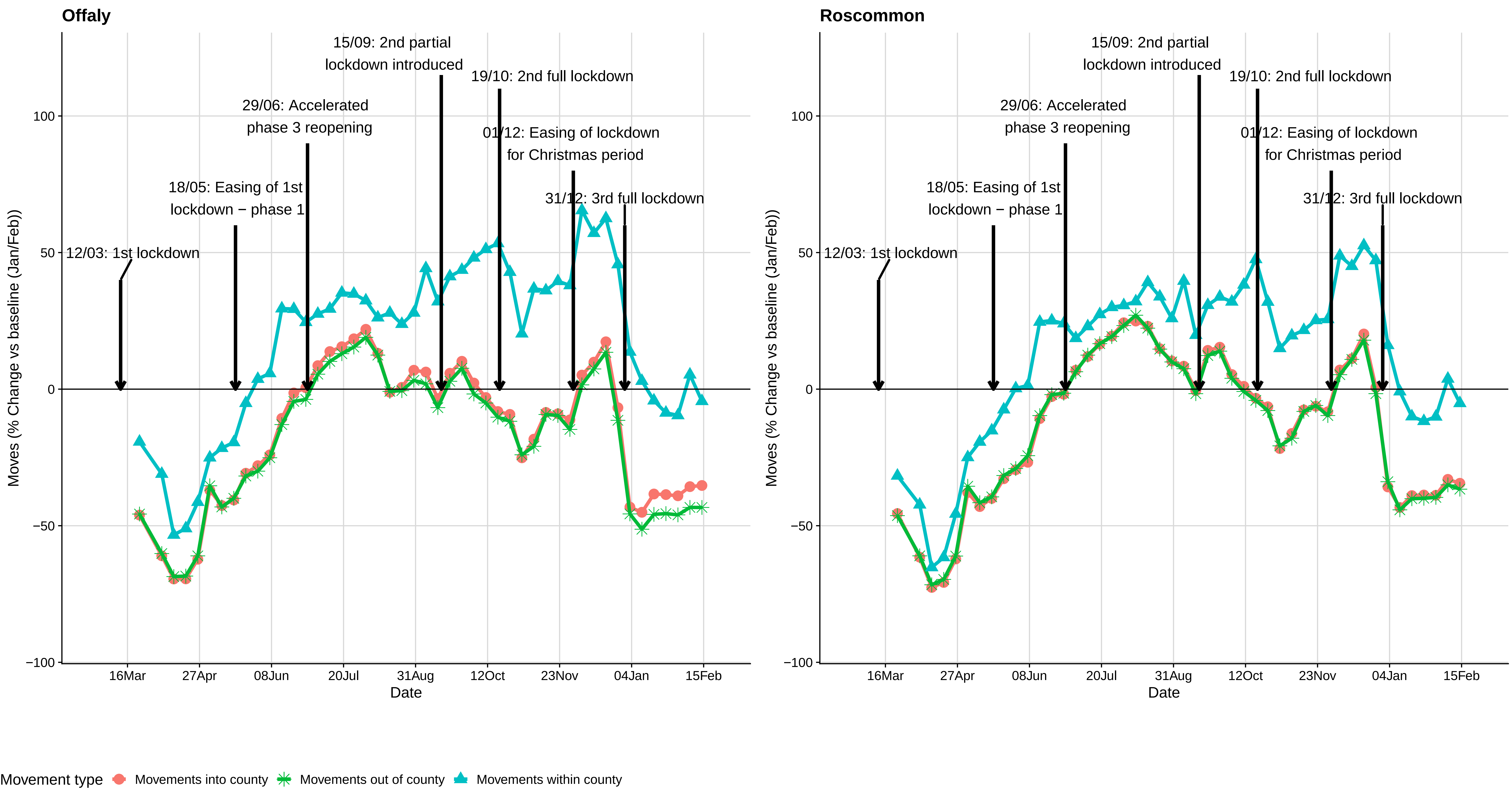
Offaly
Roscommon
15/09: 2nd partial lockdown introduced
19/10: 2nd full lockdown
29/06: Accelerated phase 3 reopening
01/12: Easing of lockdown for Christmas period
18/05: Easing of 1st lockdown − phase 1
31/12: 3rd full lockdown
12/03: 1st lockdown
Moves (% Change vs baseline (Jan/Feb))
100
50
0
−50
−100
16Mar
27Apr
08Jun
20Jul
31Aug
12Oct
23Nov
04Jan
15Feb
Date
Movement type
Movements into county
Movements out of county
Movements within county

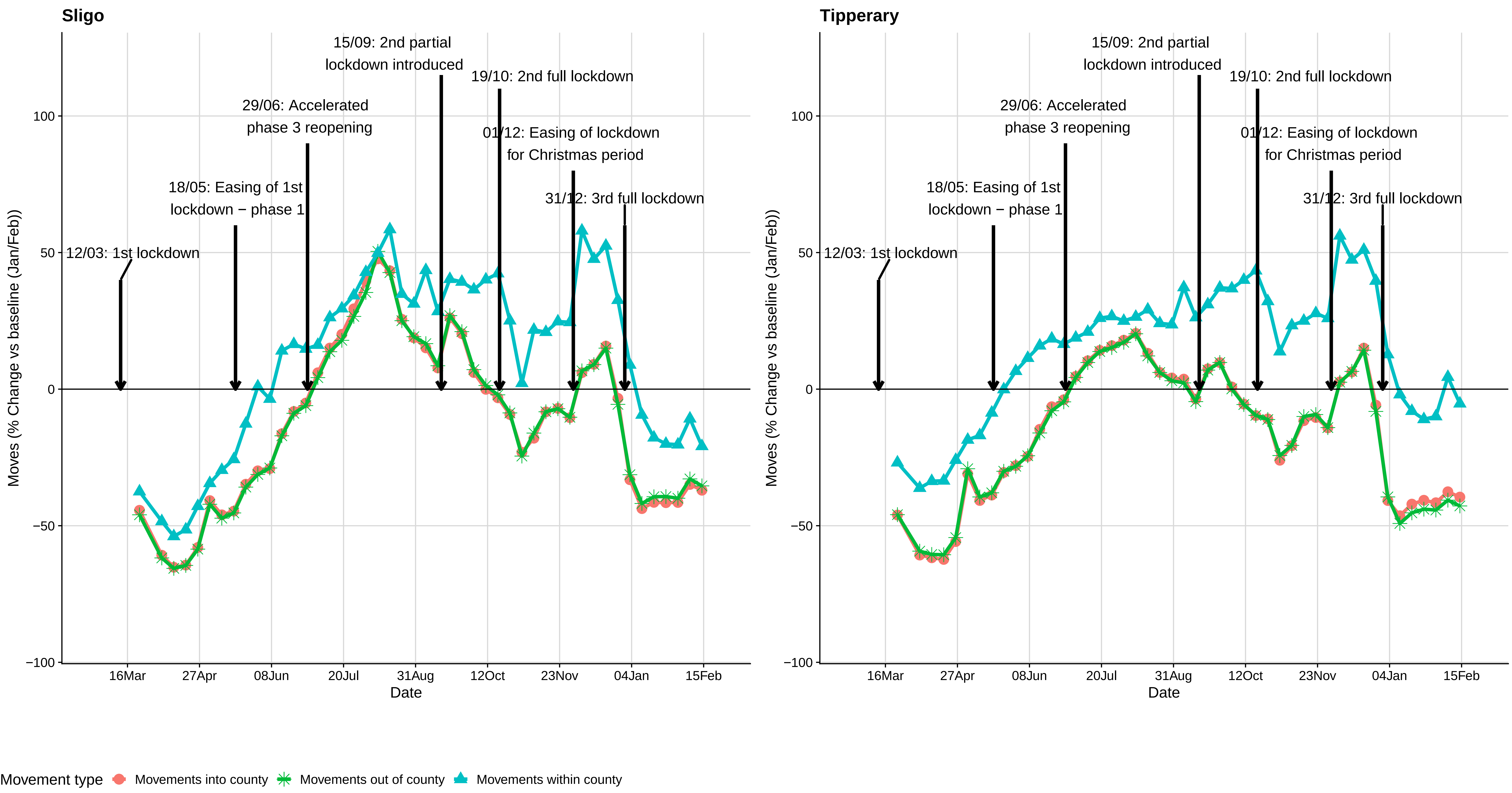
Sligo
Tipperary
15/09: 2nd partial lockdown introduced
19/10: 2nd full lockdown
29/06: Accelerated phase 3 reopening
01/12: Easing of lockdown for Christmas period
18/05: Easing of 1st lockdown − phase 1
31/12: 3rd full lockdown
12/03: 1st lockdown
Moves (% Change vs baseline (Jan/Feb))
100
50
0
−50
−100
16Mar
27Apr
08Jun
20Jul
31Aug
12Oct
23Nov
04Jan
15Feb
Date
Movement type
Movements into county
Movements out of county
Movements within county

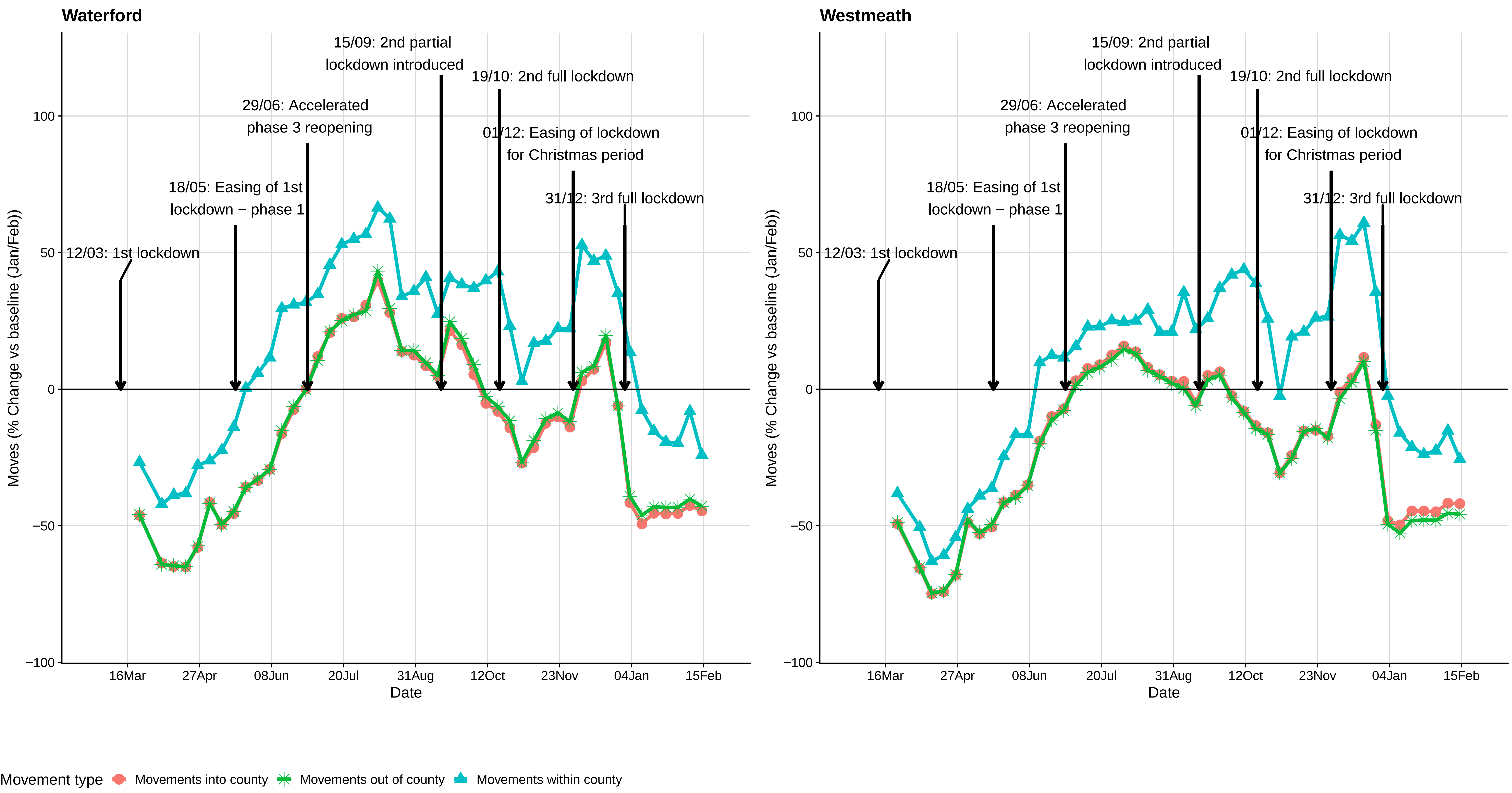
Waterford
Westmeath
12/03: 1st lockdown
18/05: Easing of 1st lockdown − phase 1
29/06: Accelerated phase 3 reopening
15/09: 2nd partial lockdown introduced
19/10: 2nd full lockdown
01/12: Easing of lockdown for Christmas period
31/12: 3rd full lockdown
Moves (% Change vs baseline (Jan/Feb))
100
50
0
−50
−100
16Mar
27Apr
08Jun
20Jul
31Aug
12Oct
23Nov
04Jan
15Feb
Date
Movement type
Movements into county
Movements out of county
Movements within county

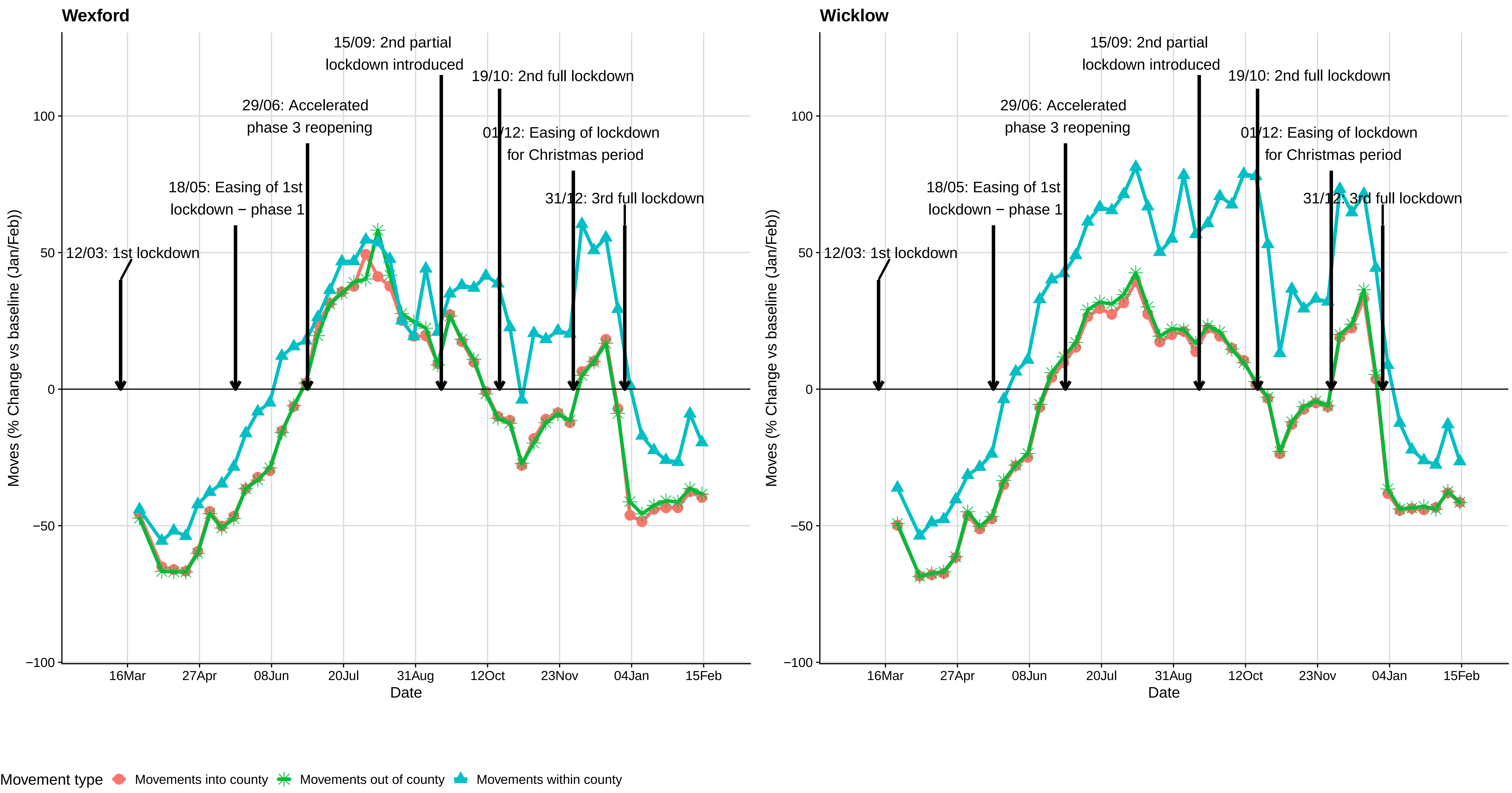
Wexford
15/09: 2nd partial lockdown introduced
19/10: 2nd full lockdown
29/06: Accelerated phase 3 reopening
01/12: Easing of lockdown for Christmas period
18/05: Easing of 1st lockdown − phase 1
31/12: 3rd full lockdown
12/03: 1st lockdown
Moves (% Change vs baseline (Jan/Feb))
100
50
0
−50
−100
16Mar
27Apr
08Jun
20Jul
31Aug
12Oct
23Nov
04Jan
15Feb
Date
Wicklow
15/09: 2nd partial lockdown introduced
19/10: 2nd full lockdown
29/06: Accelerated phase 3 reopening
01/12: Easing of lockdown for Christmas period
18/05: Easing of 1st lockdown − phase 1
31/12: 3rd full lockdown
12/03: 1st lockdown
Moves (% Change vs baseline (Jan/Feb))
Date
Movement type
Movements into county
Movements out of county
Movements within county
