# Supplementary material for: Population Mobility Trends, Deprivation Index and the Spatio-Temporal Spread of Coronavirus Disease 2019 in Ireland
Source: Int J Environ Res Public Health. 2021 Jun 10;18(12):6285. doi: 10.3390/ijerph18126285 (PMC8296107; doi:10.3390/ijerph18126285)

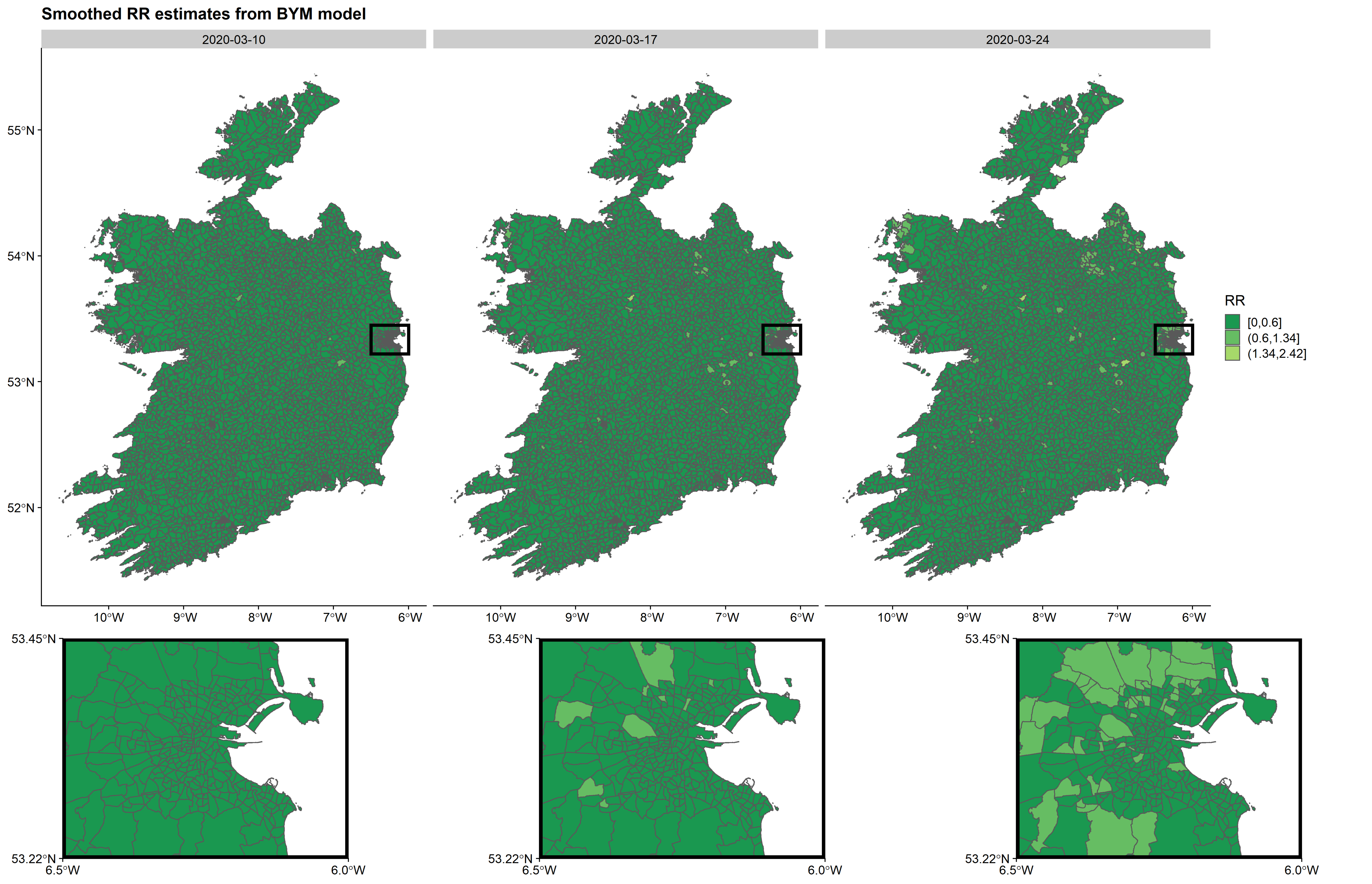
Smoothed RR estimates from BYM model
2020-03-10
2020-03-17
2020-03-24
55°N
54°N
53°N
52°N
10°W
9°W
8°W
7°W
6°W
RR
[0,0.6]
(0.6,1.34]
(1.34,2.42]
53.45°N
53.22°N
6.5°W
6.0°W

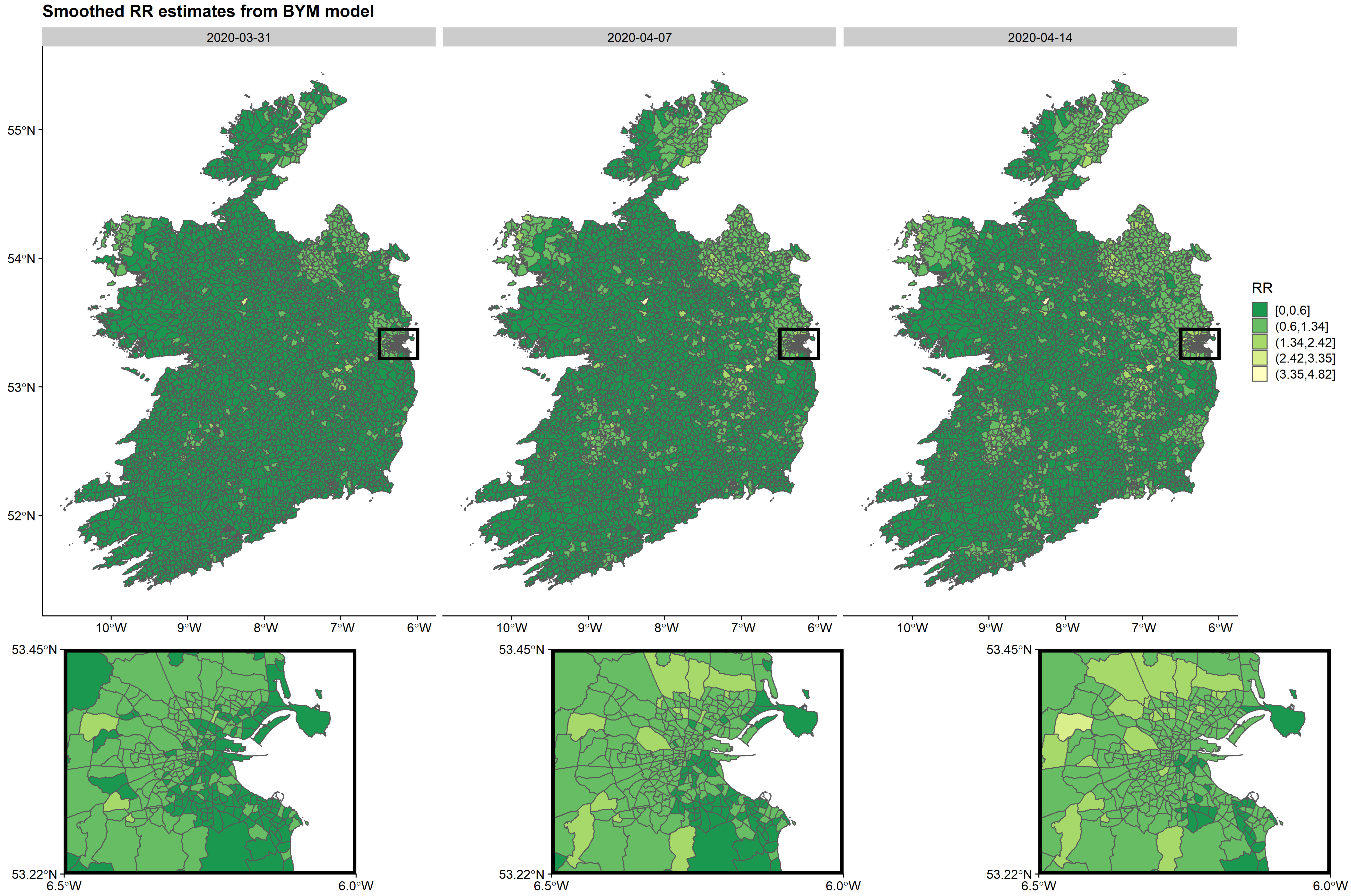

Smoothed RR estimates from BYM model
2020-03-31
2020-04-07
2020-04-14
55°N
54°N
53°N
52°N
10°W
9°W
8°W
7°W
6°W
RR
[0,0.6]
(0.6,1.34]
(1.34,2.42]
(2.42,3.35]
(3.35,4.82]
53.45°N
53.22°N
6.5°W
6.0°W

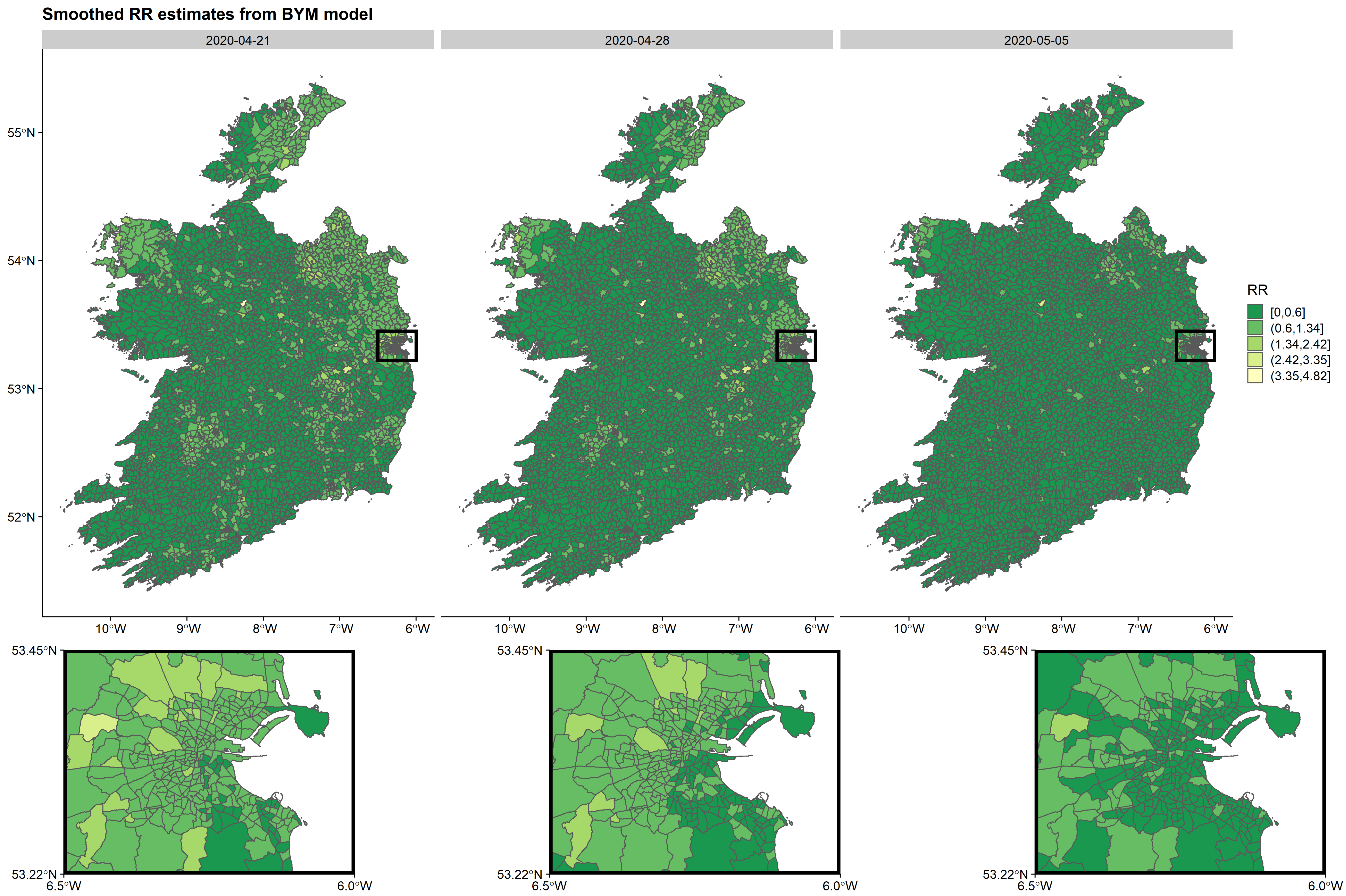

Smoothed RR estimates from BYM model
2020-04-21
2020-04-28
2020-05-05
55°N
54°N
53°N
52°N
10°W
9°W
8°W
7°W
6°W
RR
[0,0.6]
(0.6,1.34]
(1.34,2.42]
(2.42,3.35]
(3.35,4.82]
53.45°N
53.22°N
6.5°W
6.0°W

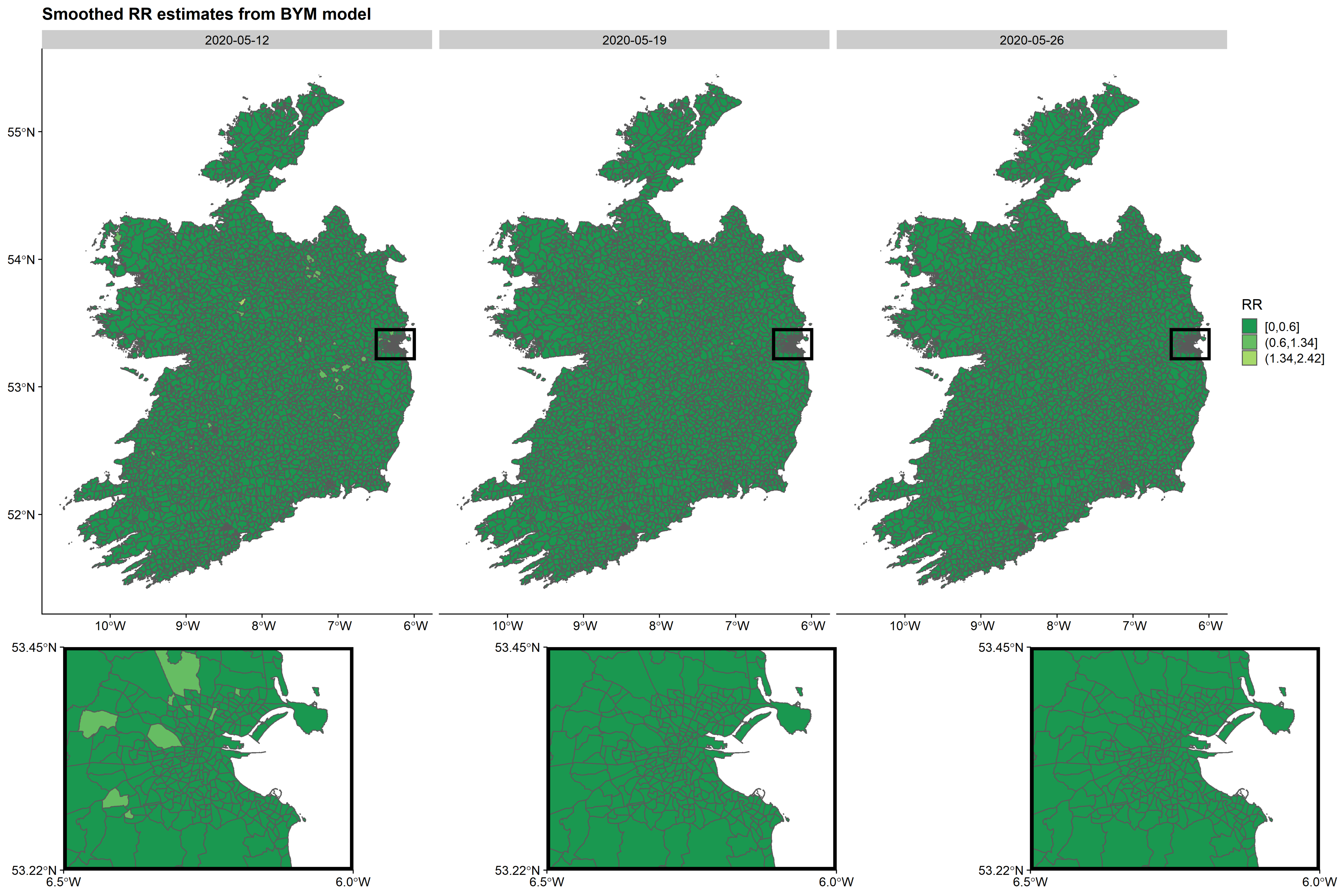
Smoothed RR estimates from BYM model
2020-05-12
2020-05-19
2020-05-26
55°N
54°N
53°N
52°N
10°W
9°W
8°W
7°W
6°W
RR
[0,0.6]
(0.6,1.34]
(1.34,2.42]
53.45°N
53.22°N
6.5°W
6.0°W

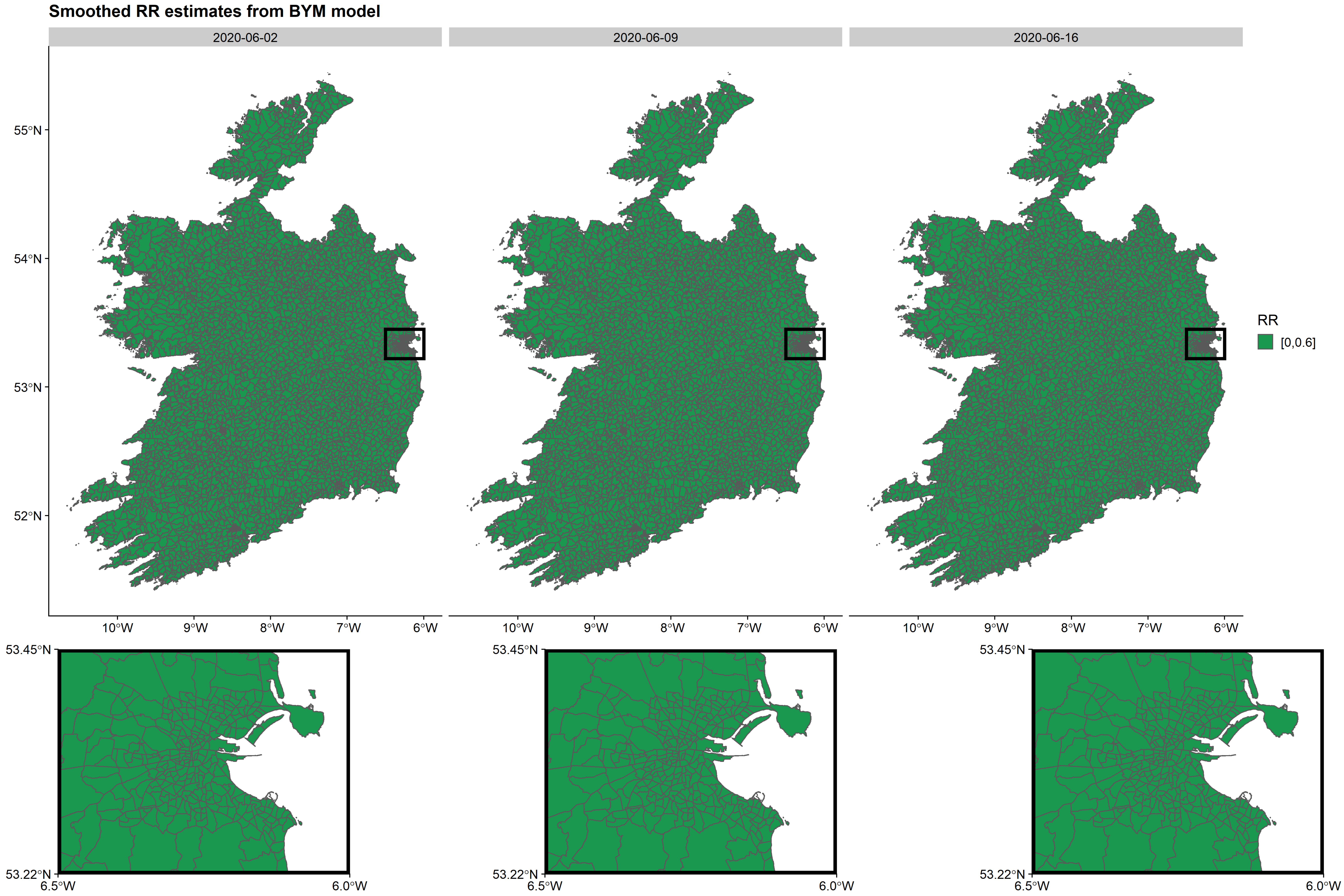
Smoothed RR estimates from BYM model
2020-06-02
2020-06-09
2020-06-16
55°N
54°N
53°N
52°N
10°W
9°W
8°W
7°W
6°W
RR
[0,0.6]
53.45°N
53.22°N
6.5°W
6.0°W

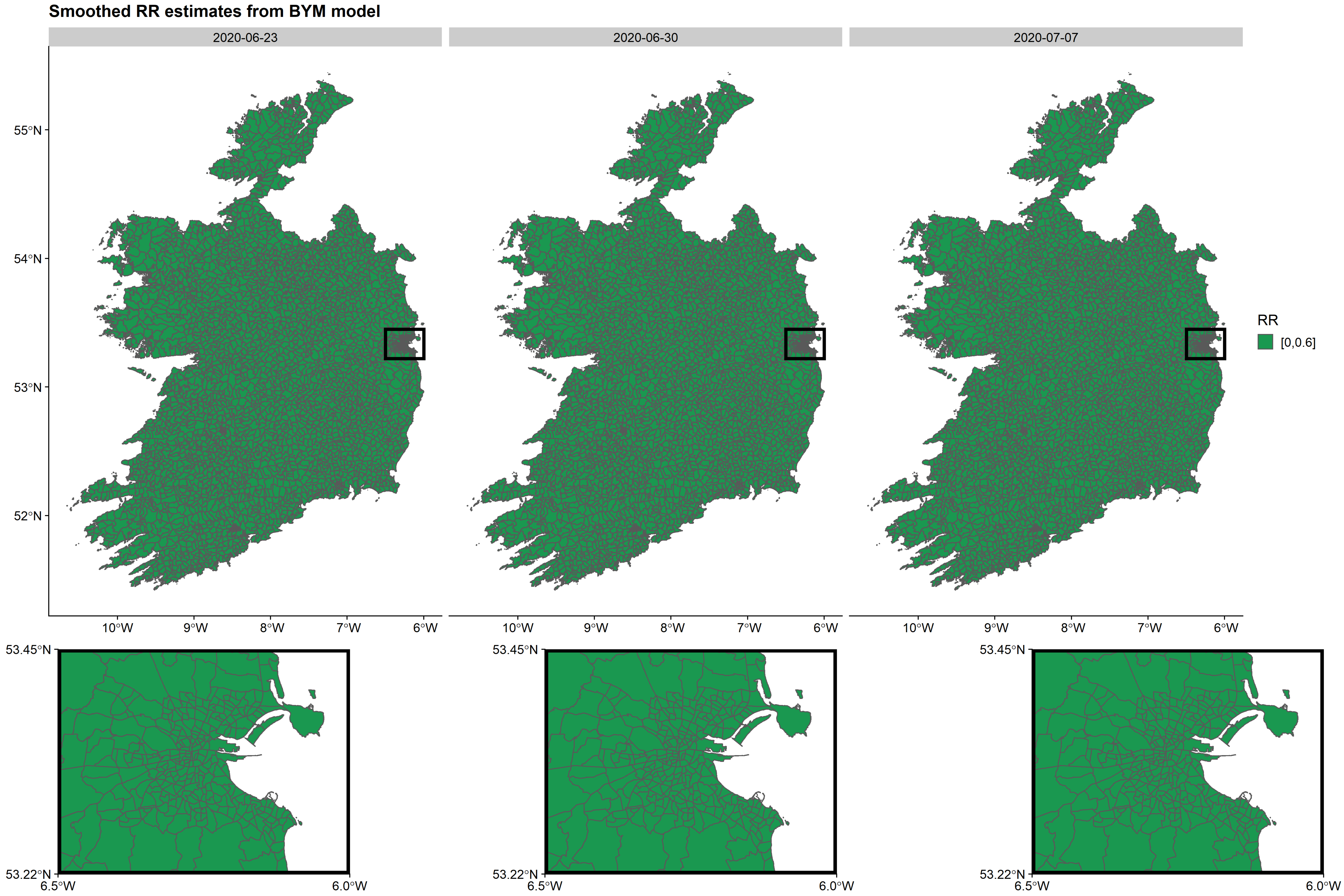

Smoothed RR estimates from BYM model
2020-06-23
2020-06-30
2020-07-07
55°N
54°N
53°N
52°N
10°W
9°W
8°W
7°W
6°W
RR
[0,0.6]
53.45°N
53.22°N
6.5°W
6.0°W

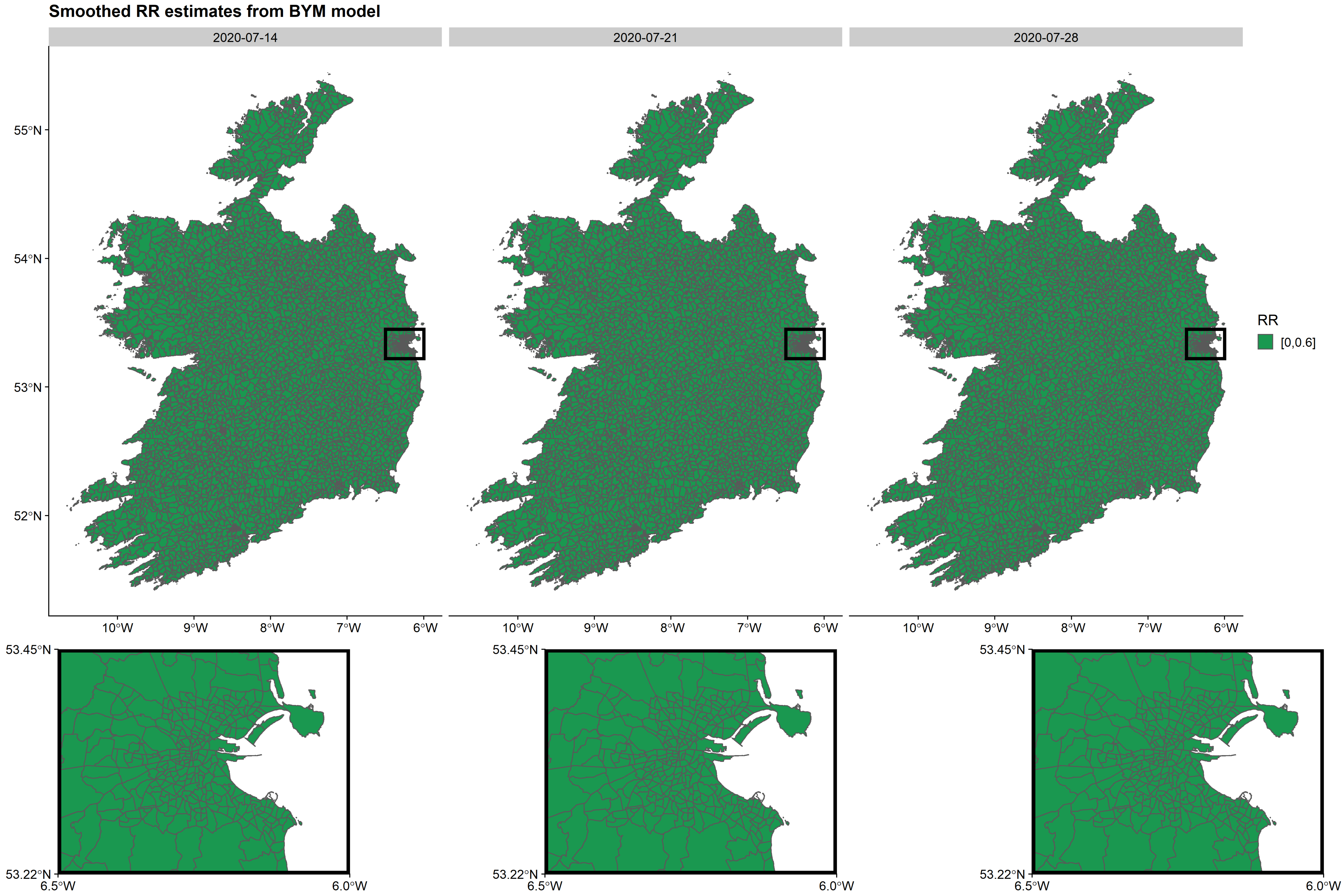

Smoothed RR estimates from BYM model
2020-07-14
2020-07-21
2020-07-28
55°N
54°N
53°N
52°N
10°W
9°W
8°W
7°W
6°W
RR
[0,0.6]
53.45°N
53.22°N
6.5°W
6.0°W

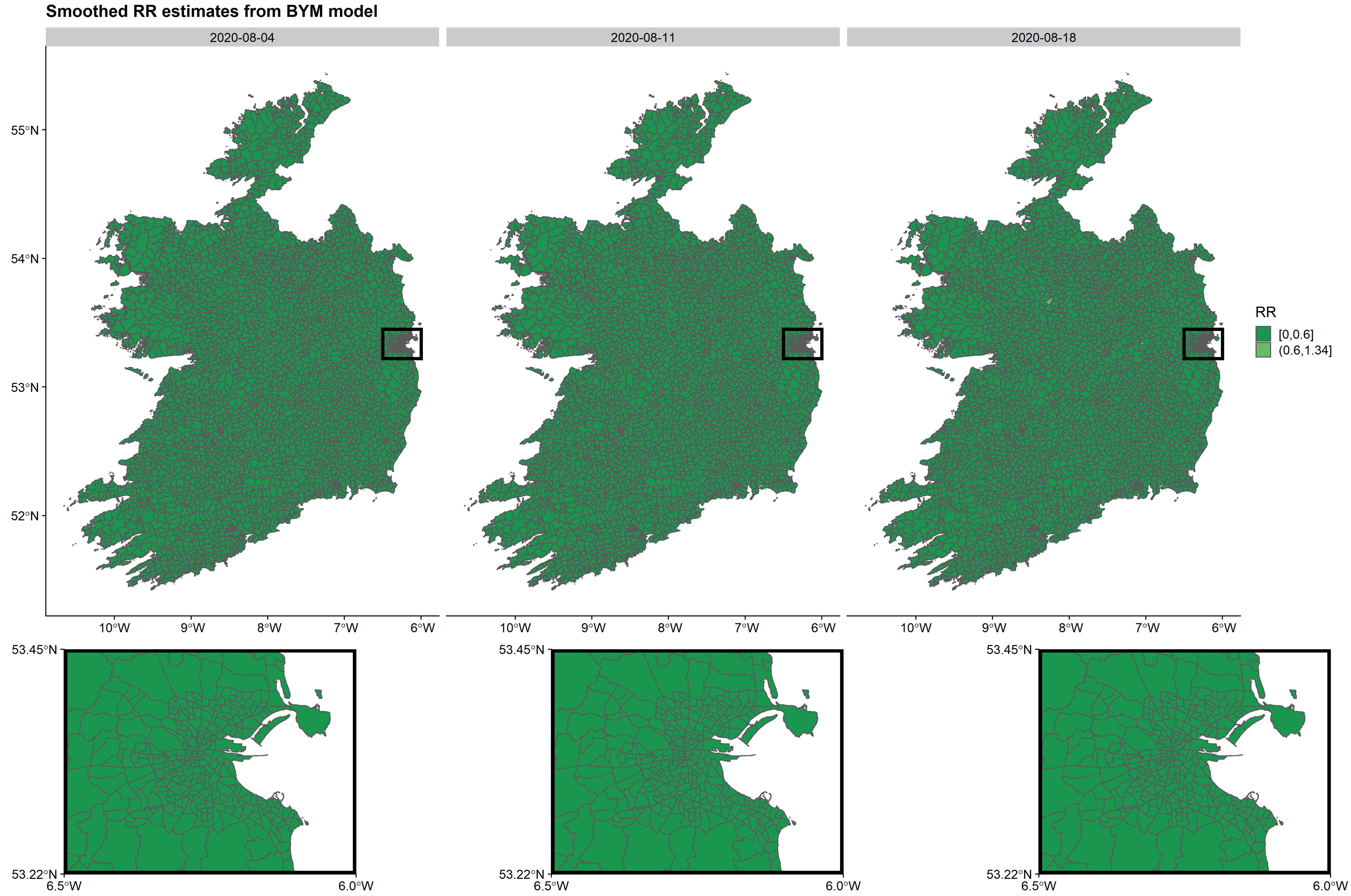

Smoothed RR estimates from BYM model
2020-08-04
2020-08-11
2020-08-18
55°N
54°N
53°N
52°N
10°W
9°W
8°W
7°W
6°W
RR
[0,0.6]
(0.6,1.34]
53.45°N
53.22°N
6.5°W
6.0°W

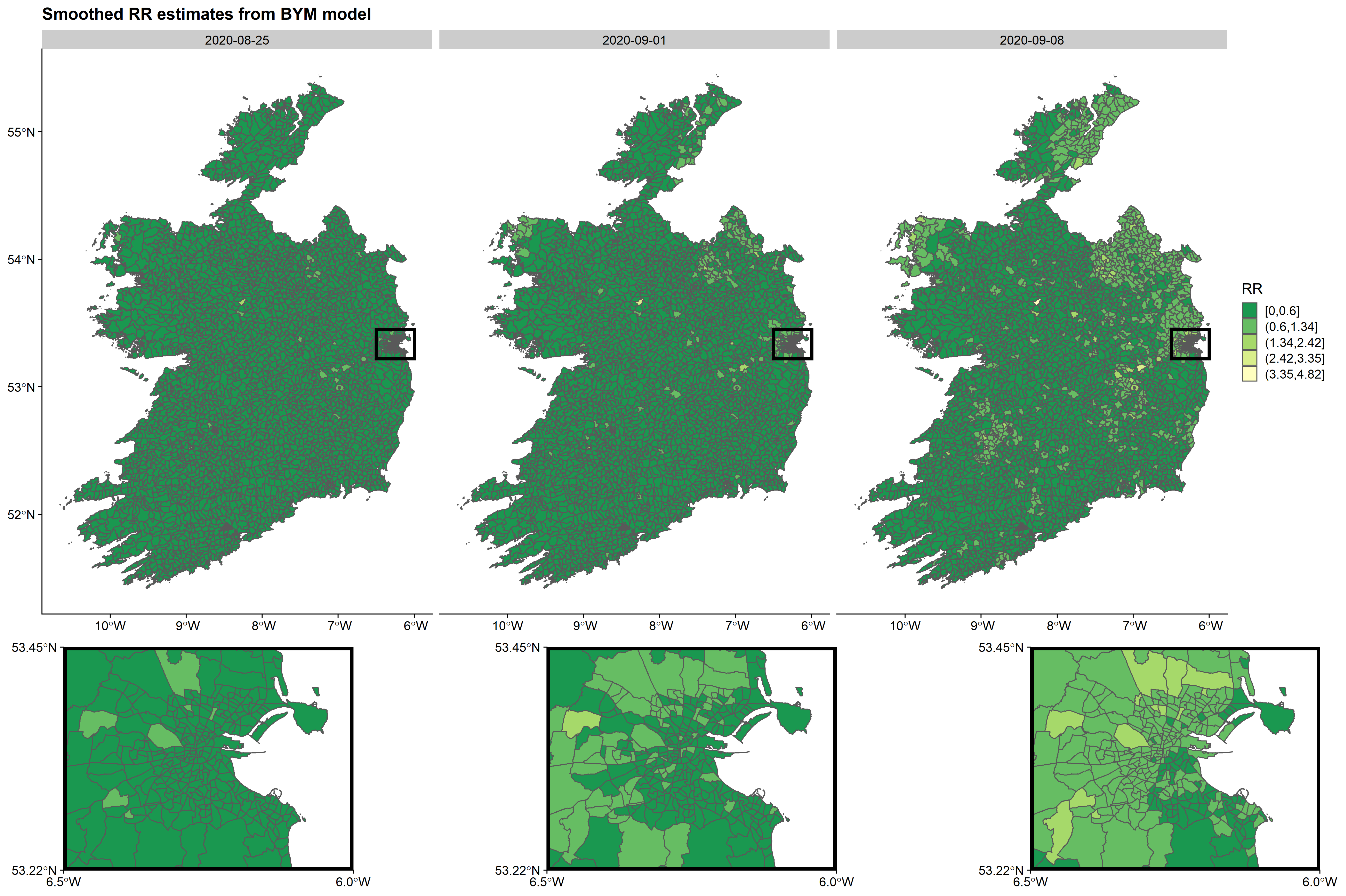
Smoothed RR estimates from BYM model
2020-08-25
2020-09-01
2020-09-08
55°N
54°N
53°N
52°N
10°W
9°W
8°W
7°W
6°W
RR
[0,0.6]
(0.6,1.34]
(1.34,2.42]
(2.42,3.35]
(3.35,4.82]
53.45°N
53.22°N
6.5°W
6.0°W

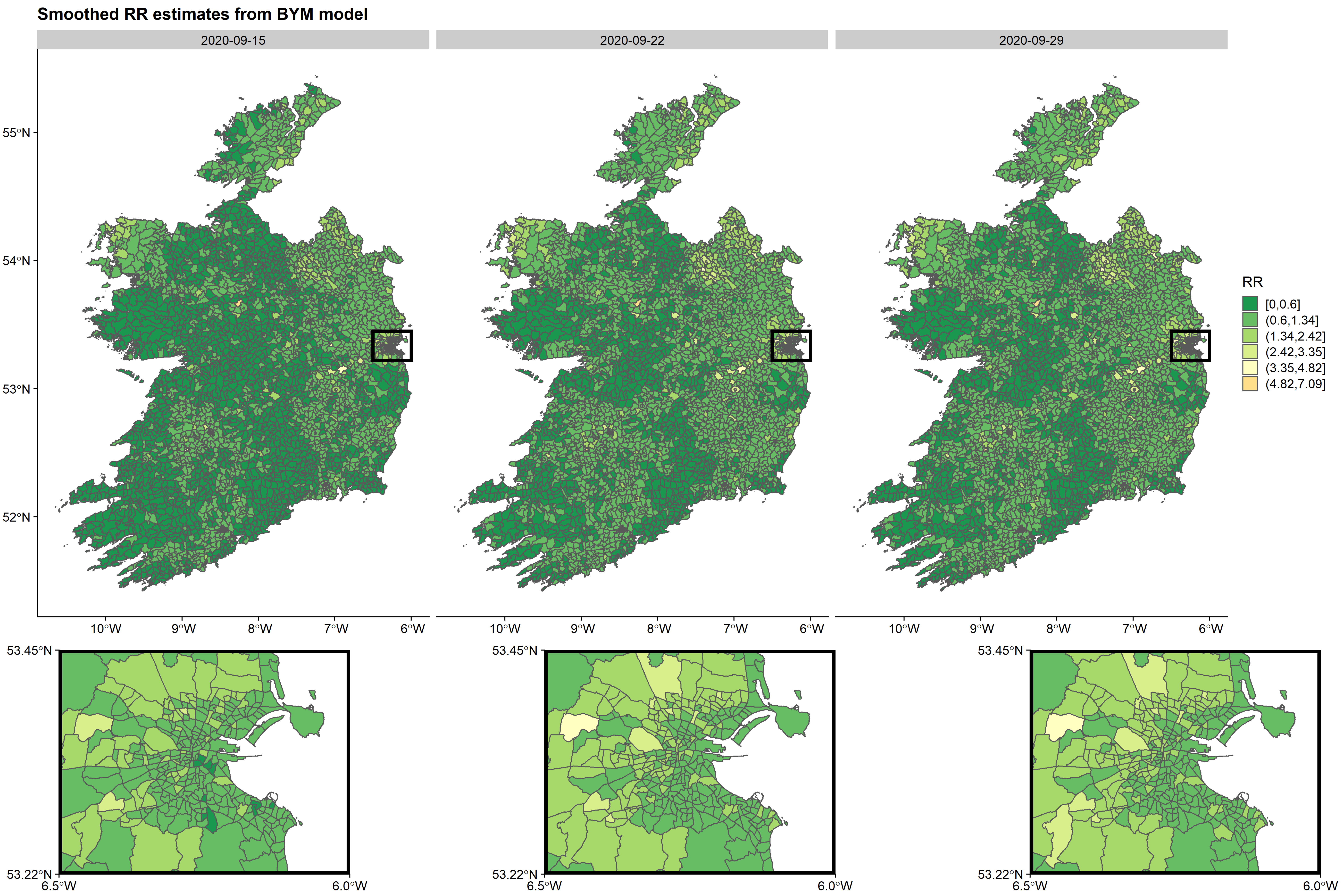
Smoothed RR estimates from BYM model
2020-09-15
2020-09-22
2020-09-29
55°N
54°N
53°N
52°N
10°W
9°W
8°W
7°W
6°W
RR
[0,0.6]
(0.6,1.34]
(1.34,2.42]
(2.42,3.35]
(3.35,4.82]
(4.82,7.09]
53.45°N
53.22°N
6.5°W
6.0°W

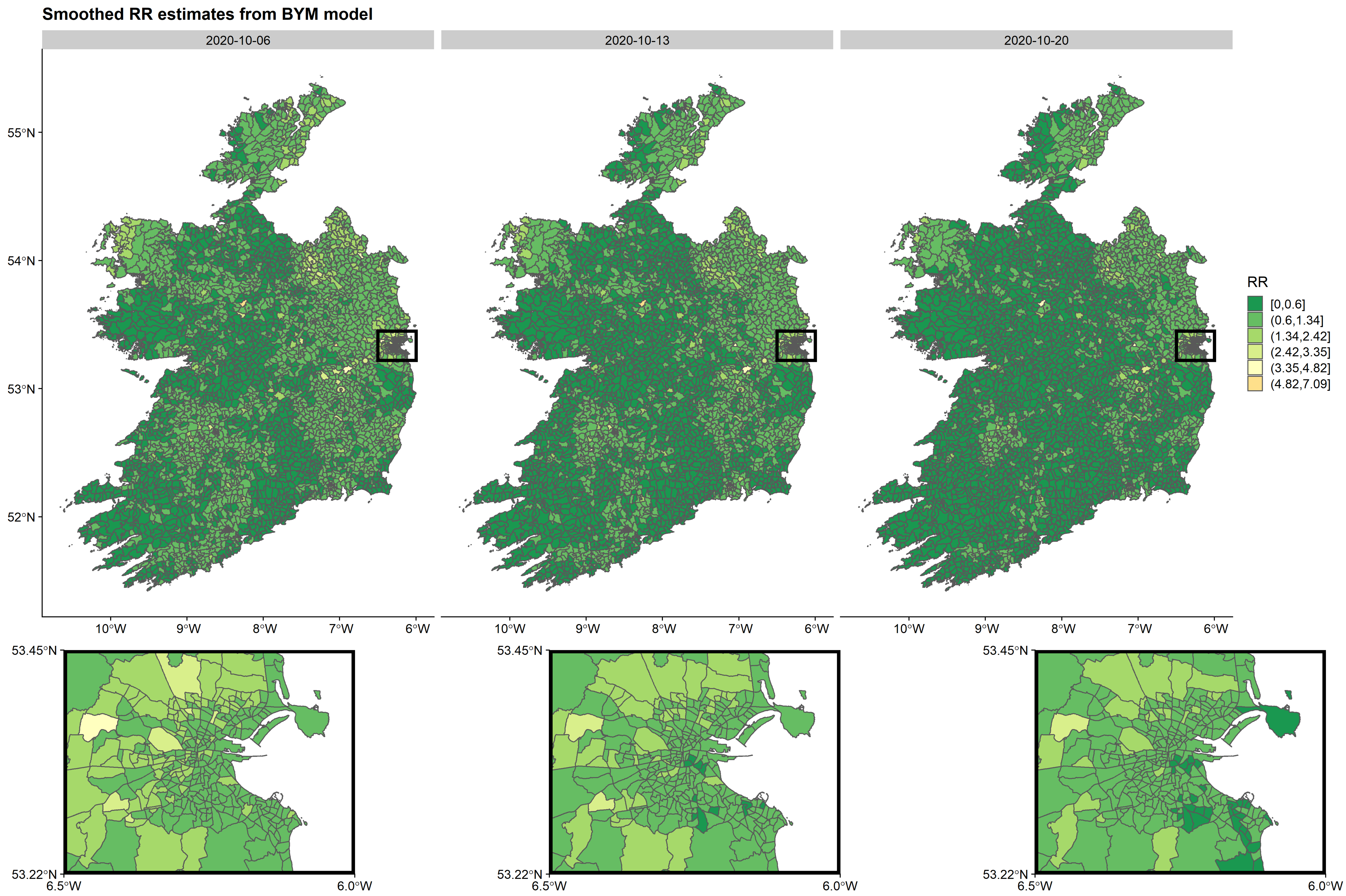
Smoothed RR estimates from BYM model
2020-10-06
2020-10-13
2020-10-20
55°N
54°N
53°N
52°N
10°W
9°W
8°W
7°W
6°W
RR
[0,0.6]
(0.6,1.34]
(1.34,2.42]
(2.42,3.35]
(3.35,4.82]
(4.82,7.09]
53.45°N
53.22°N
6.5°W
6.0°W

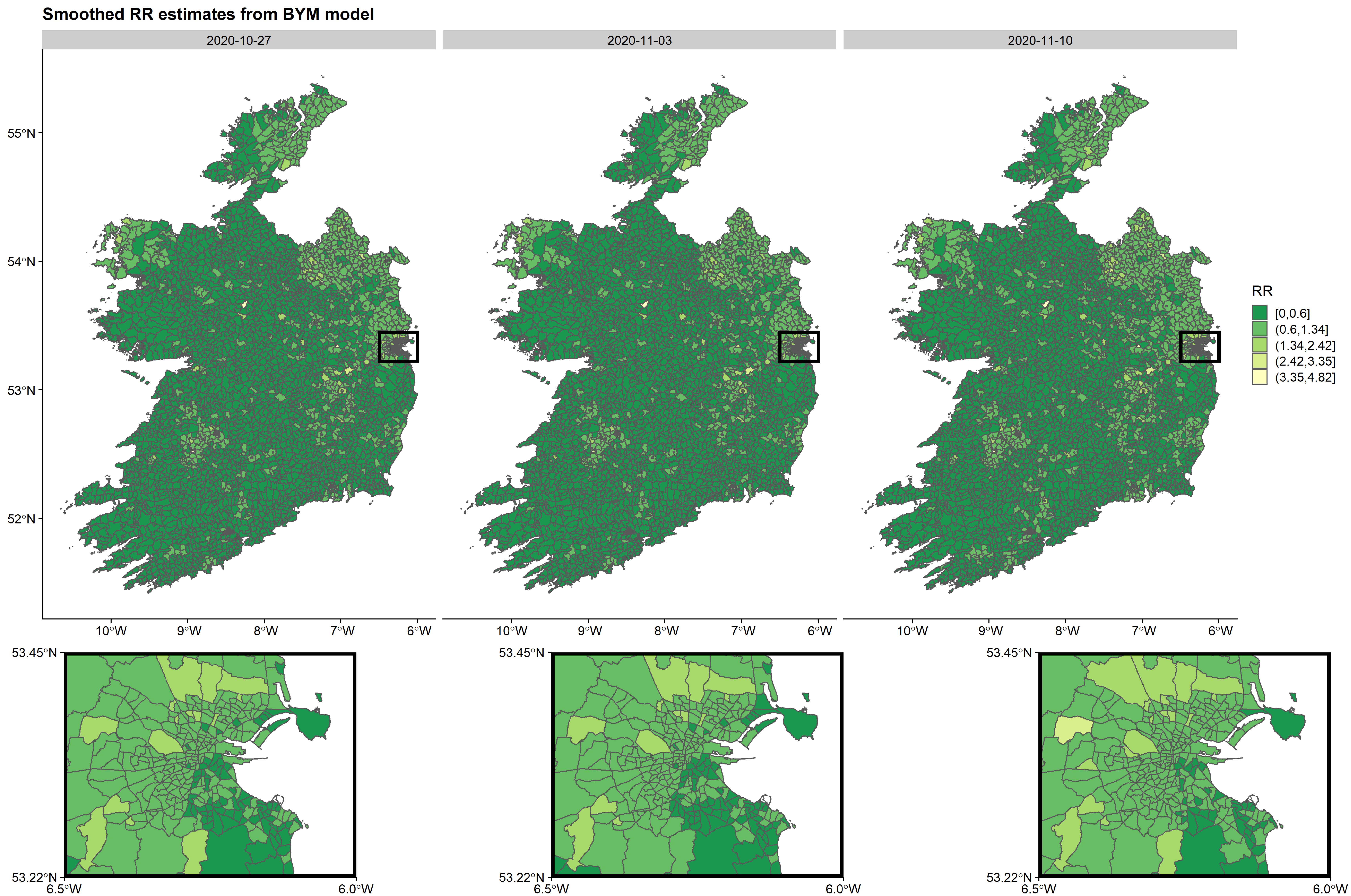
Smoothed RR estimates from BYM model
2020-10-27
2020-11-03
2020-11-10
55°N
54°N
53°N
52°N
10°W
9°W
8°W
7°W
6°W
RR
[0,0.6]
(0.6,1.34]
(1.34,2.42]
(2.42,3.35]
(3.35,4.82]
53.45°N
53.22°N
6.5°W
6.0°W

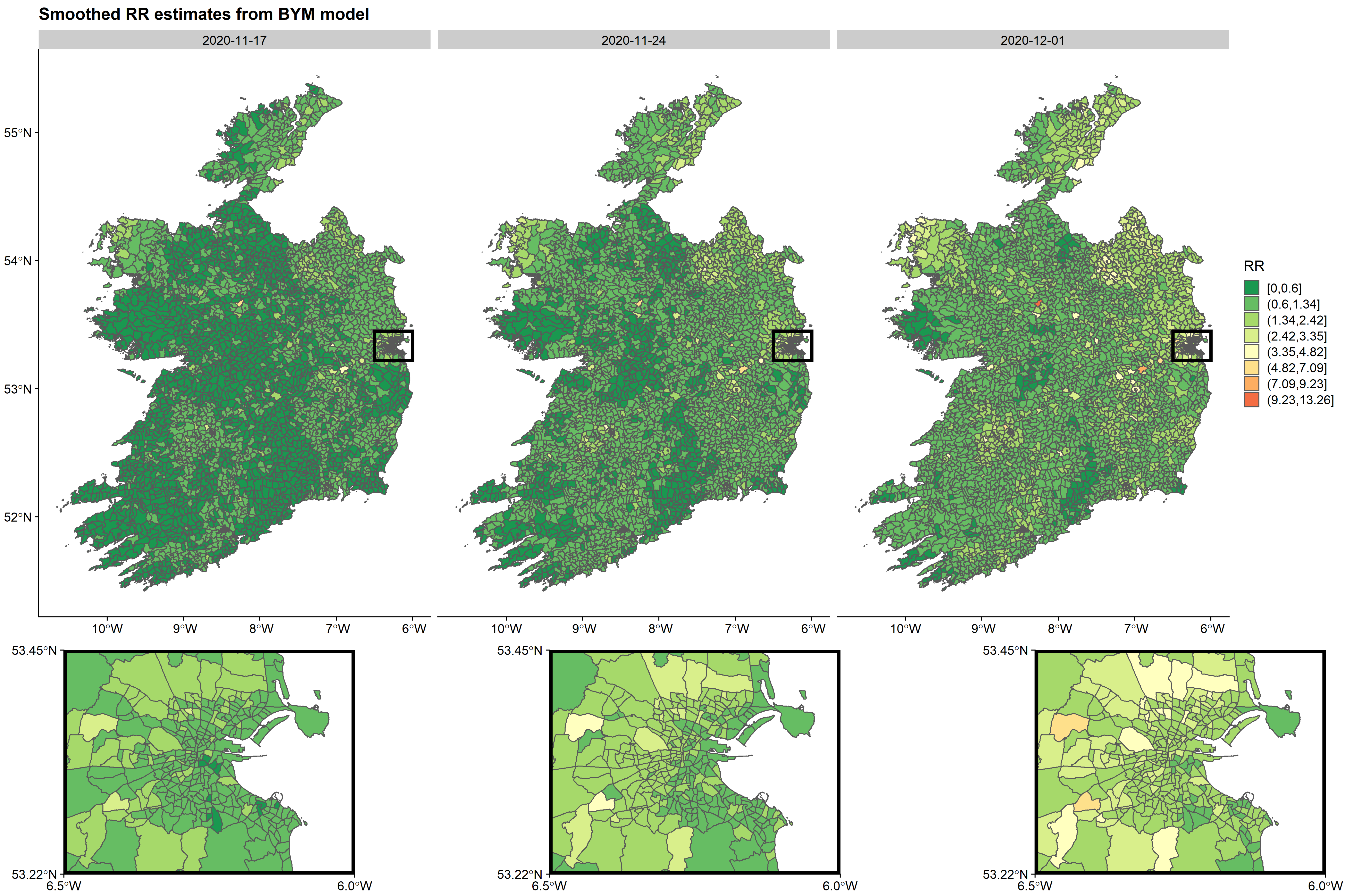

Smoothed RR estimates from BYM model
2020-11-17
2020-11-24
2020-12-01
55°N
54°N
53°N
52°N
10°W
9°W
8°W
7°W
6°W
RR
[0,0.6]
(0.6,1.34]
(1.34,2.42]
(2.42,3.35]
(3.35,4.82]
(4.82,7.09]
(7.09,9.23]
(9.23,13.26]
53.45°N
53.22°N
6.5°W
6.0°W

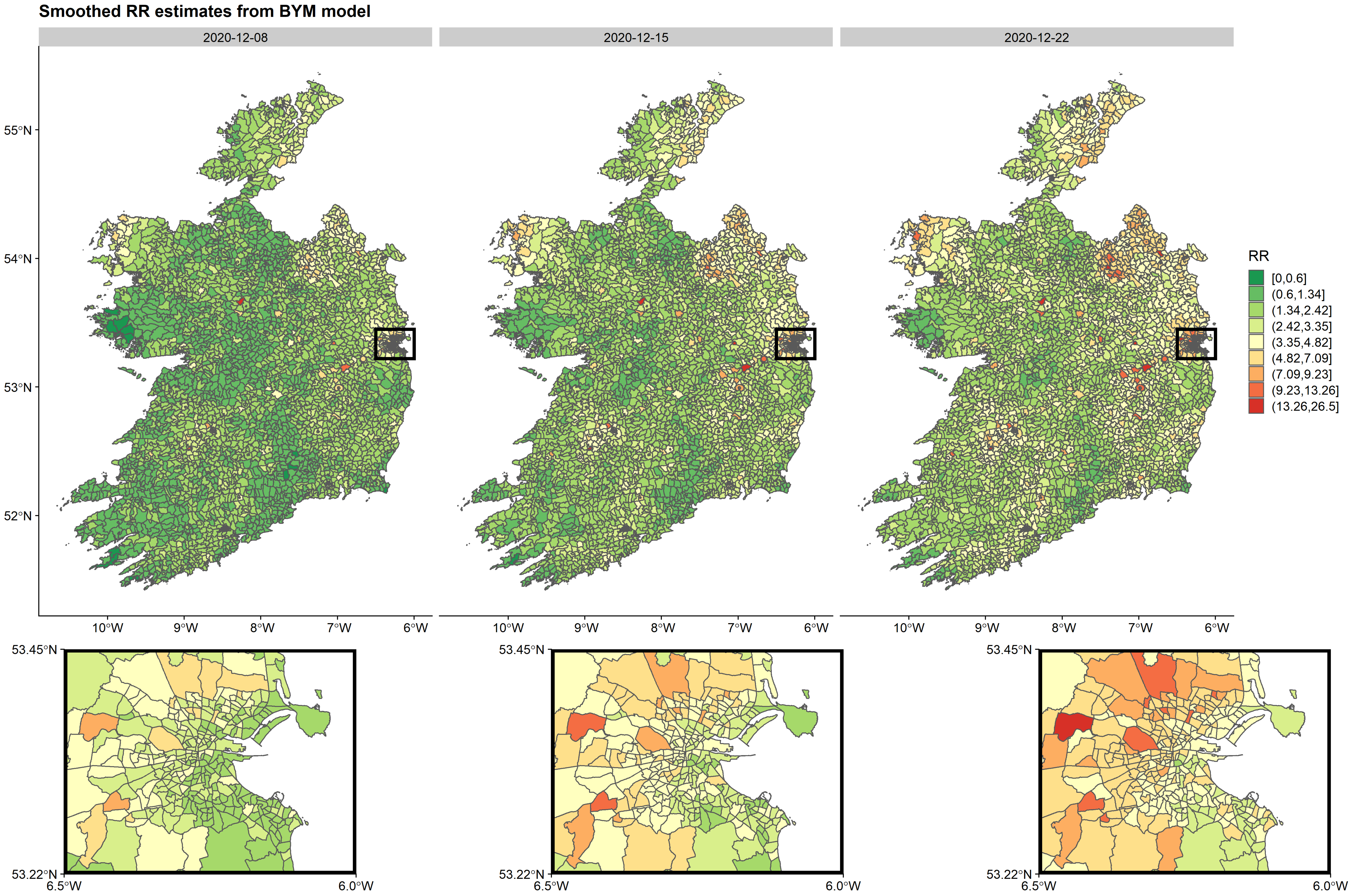

Smoothed RR estimates from BYM model
2020-12-08
2020-12-15
2020-12-22
55°N
54°N
53°N
52°N
10°W
9°W
8°W
7°W
6°W
RR
[0,0.6]
(0.6,1.34]
(1.34,2.42]
(2.42,3.35]
(3.35,4.82]
(4.82,7.09]
(7.09,9.23]
(9.23,13.26]
(13.26,26.5]
53.45°N
53.22°N
6.5°W
6.0°W

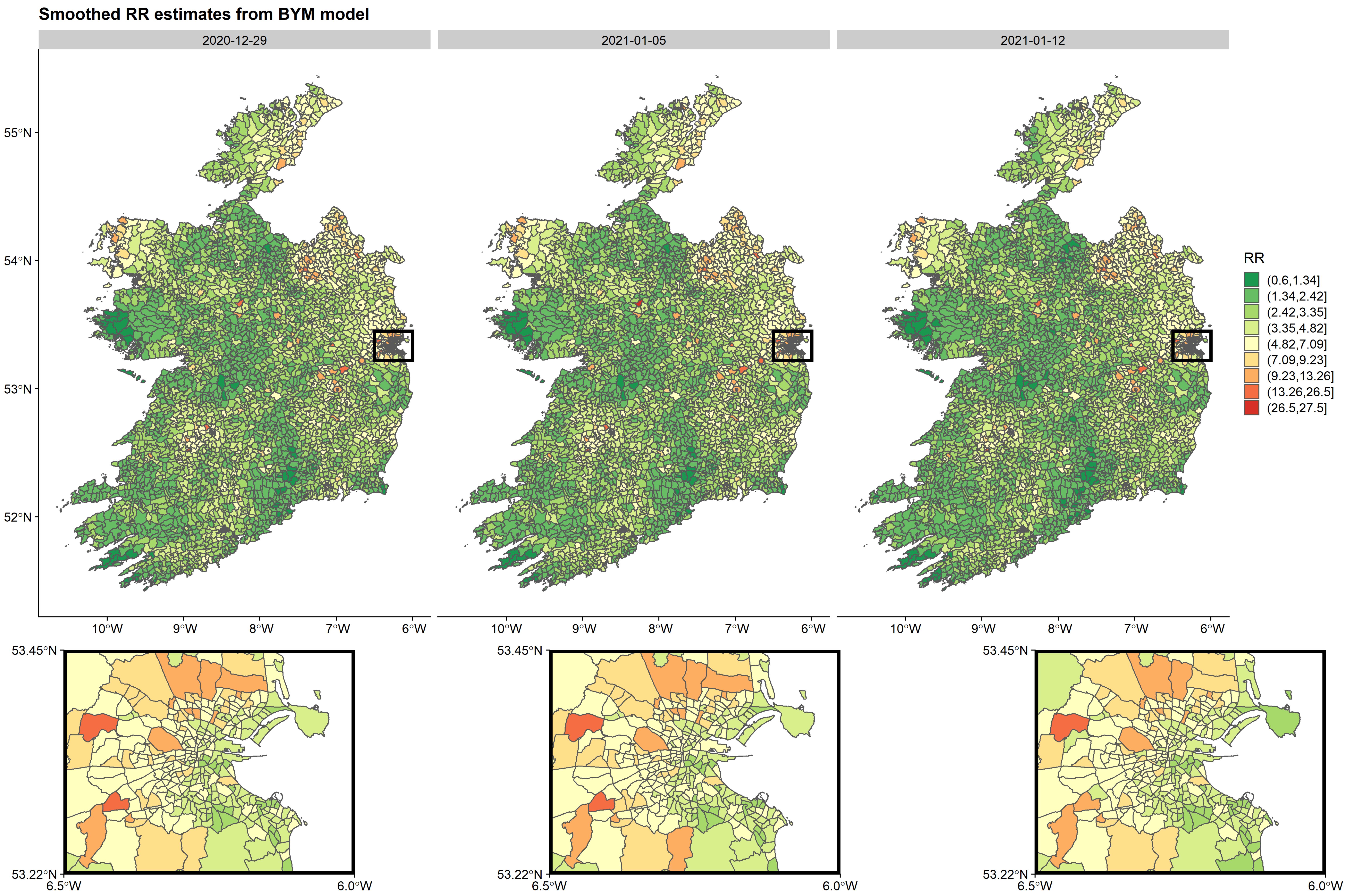
Smoothed RR estimates from BYM model
2020-12-29
2021-01-05
2021-01-12
55°N
54°N
53°N
52°N
10°W
9°W
8°W
7°W
6°W
RR
(0.6,1.34]
(1.34,2.42]
(2.42,3.35]
(3.35,4.82]
(4.82,7.09]
(7.09,9.23]
(9.23,13.26]
(13.26,26.5]
(26.5,27.5]
53.45°N
53.22°N
6.5°W
6.0°W

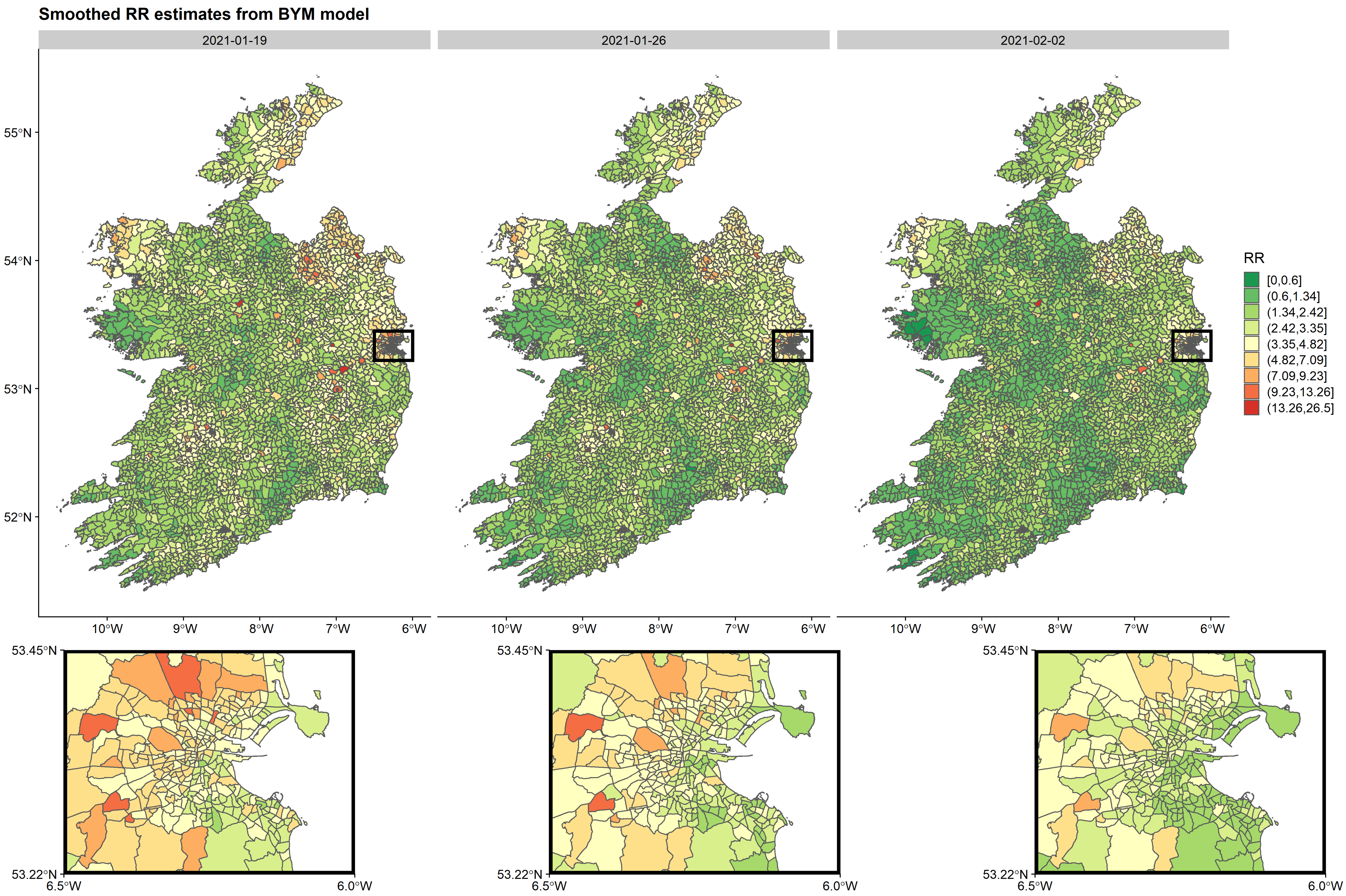

Smoothed RR estimates from BYM model
2021-01-19
2021-01-26
2021-02-02
55°N
54°N
53°N
52°N
10°W
9°W
8°W
7°W
6°W
RR
[0,0.6]
(0.6,1.34]
(1.34,2.42]
(2.42,3.35]
(3.35,4.82]
(4.82,7.09]
(7.09,9.23]
(9.23,13.26]
(13.26,26.5]
53.45°N
53.22°N
6.5°W
6.0°W

Smoothed RR estimates from BYM model

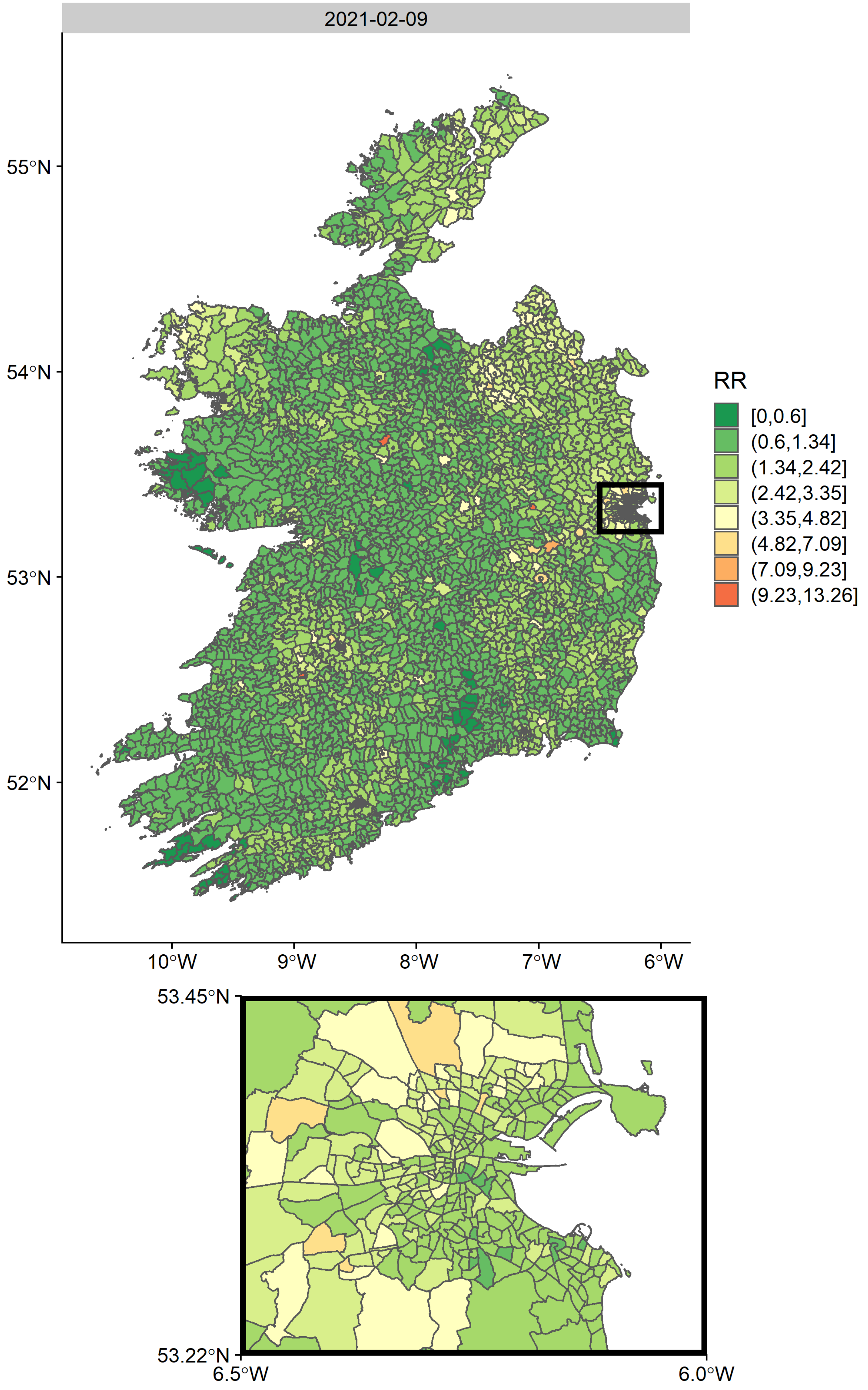

Supplement: Supplementary file 1 [file ijerph-18-06285-s001.zip › SM4_RR_per_week_inset_05_Mar_2021.pdf]
